# QbTest for ADHD assessment and medication management: a mixed-methods systematic review of impact on clinical outcomes and patient, carer and clinician experiences

**DOI:** 10.1136/bmjopen-2024-095479

**Published:** 2025-04-17

**Authors:** Eve Tomlinson, Amanda Owen-Smith, Melissa Benavente, Chris Cooper, Hayley E Jones, Mary Ward, Josephine G Walker, Hanyu Wang, Catalina Lopez Manzano, Dietmar Hank, Nicky J Welton, Mariska Leeflang, Penny Whiting

**Affiliations:** 1 Population Health Sciences, Bristol Medical School, University of Bristol, Bristol, UK; 2 Patient Representative, Bristol, UK; 3 Avon and Wiltshire Mental Health Partnership NHS Trust, Bath, UK; 4 Department of Epidemiology and Data Science, Amsterdam University Medical Centres, Amsterdam, The Netherlands

**Keywords:** Health Services, PSYCHIATRY, QUALITATIVE RESEARCH, Attention Deficit Disorder with Hyperactivity, Child & adolescent psychiatry, Review

## Abstract

**Abstract:**

**Objectives:**

To explore patient, carer and clinician experiences of the QbTest and its impact on patient outcomes for attention deficit hyperactivity disorder (ADHD) diagnosis and medication management.

**Design:**

Mixed-methods systematic review.

**Data sources:**

MEDLINE, EMBASE, PsycINFO, CINAHL, ClinicalTrials.gov and WHO ICTRP (from inception to September 2024).

**Study selection:**

Primary studies, of any design, that evaluated any version of the QbTest (QbMini <5 years, QbTest 6–12 or 12–60 years, QbCheck for remote assessment via webcam or QbMT smartphone version), for ADHD diagnosis and/or medication management and provided data on any of the following outcomes, were eligible: time to assessment/diagnostic decision, use of services, impact on clinical decision-making, healthcare professionals’ confidence in assessment, intervention use, morbidity, mortality, health-related quality of life, cost, ease of use, experience and acceptability of the test to patients, carers and clinicians.

**Data extraction and synthesis:**

Two reviewers independently screened titles and abstracts and assessed potentially relevant reports for inclusion. One reviewer conducted data extraction and risk of bias (RoB) assessment, checked by a second reviewer. Mixed-methods synthesis followed the convergent-integrated approach.

**Results:**

We identified 10 eligible studies (9 QbTest; 1 QbCheck), including 1 randomised controlled trial (RCT), 2 feasibility RCTs, 5 before-and-after studies, 1 mixed-methods study and 1 diagnostic study. Most studies enrolled children in the UK and included surveys or interviews with patients, carers or clinicians. The RCT and before-and-after studies were judged at high/serious RoB. Six survey components and two qualitative interview components were judged at some concerns of RoB. We identified one ongoing study of the QbMT and no studies for QbMini. We organised themes emerging from the qualitative synthesis into two broad conceptual categories: views around the helpfulness of the QbTest (contribution to ADHD diagnosis, treatment decision-making, communication with caregivers) and barriers to QbTest implementation (practical barriers and acceptability of the test to patients and caregivers). Findings suggested that the addition of the QbTest may reduce time to diagnosis, improve clinician confidence in the diagnostic decision, increase the proportion of patients with a diagnostic decision and reduce cost and number of clinic appointments. The QbTest appeared to be generally well received by clinicians, patients and carers. However, barriers to test implementation were reported. Clinicians cited staffing, room requirements and issues with technology, and patients highlighted the test length and repetitive nature. Little data exist on the use of the QbTest for medication management.

**Conclusions:**

The available evidence suggests the QbTest may be a useful addition to ADHD assessment in children and young people. Further well-designed RCTs with qualitative substudies are required to assess the impact of the QbTest on patient outcomes, user experience and cost, particularly for medication management and in adults, where evidence is scarce. Such RCTs should include economic analyses, direct comparisons to other continuous performance tests with motion trackers and subgroup analyses including age, sex, ethnicity and comorbidities.

**PROSPERO registration number:**

CRD42023482963.

**Strengths and limitations of this study**

- This systematic review integrates quantitative and qualitative evidence concerning the impact of the QbTest on attention deficit hyperactivity disorder diagnosis and medication management, and the acceptability of the test to patients, carers and clinicians.
- We followed published guidance on the conduct and reporting of systematic reviews and involved three patients and two clinicians in the review to maximise the usefulness of the evidence to decision-makers.
- Only 1 of the 10 included studies was a full randomised controlled trial, and many studies were judged at high risk of bias.
- Most studies were conducted in children (predominantly white males in the studies that reported sex and/or ethnicity) within the UK, so it is unclear if results would be similar in other groups.
- Very little evidence is available on the use of the QbTest for medication management.

## Introduction

 Attention deficit hyperactivity disorder (ADHD) is a common neurodevelopmental condition that can be prevalent throughout the lifespan.^1^ Untreated ADHD can substantially impact on functioning and quality of life and is associated with poor academic and occupational performance, relationship difficulties, low self-esteem and comorbidities such as substance use, anxiety and depression.^1^,^4^ Timely ADHD assessment is important to ensure access to treatment.

Individuals with suspected ADHD will often be referred by a primary care professional to a specialist in secondary care for further assessment. Referral pathways vary, but for children, referral will often come from a general practitioner (GP) or education professional to a paediatrician or to child and adolescent mental health services. For adults, referral will commonly come from a GP or psychiatric service to a mental health specialist. However, long waiting lists mean that it can be years before ADHD assessment takes place.^5 6^ ADHD diagnosis typically involves a clinician gathering information from multiple sources, including behavioural observations, patient history, interviews, questionnaires and subjective reports from informants, such as caregivers and teachers for children and young people, or partners/spouses and family members for adults.^1^ The diagnostic process can be challenging and time-consuming for a number of reasons. Informants often provide contradictory perspectives which can cause uncertainty. Additionally, ADHD often co-occurs with other conditions, such as autism spectrum disorder, learning disabilities and mood disorders, which can exhibit similar symptoms to ADHD, making it difficult to distinguish between conditions.^1^ The predominant ADHD symptoms also vary between individuals, which can lead to further inconsistency in diagnosis.^7^

Following an ADHD diagnosis, management of ADHD often requires a holistic approach that addresses psychological, behavioural and occupational (for adults) or educational (for children/ young people) needs. If medication is initiated, careful dose titration and medication management is required to achieve optimal treatment while limiting side effects.^8^ This also relies on the clinician gathering subjective reports from informants about whether the treatment is working.

Objective and computerised continuous performance tests (CPTs) have the potential to improve ADHD assessment and medication management, when used in addition to clinical assessment.^9^ Most CPTs only measure inattention and impulsivity and do not include a measure of hyperactivity.^10^ The QbTest is one of the most researched and used CPTs that measures all three of these core ADHD symptoms.^11^,^14^ During the QbTest, the participant is asked to press a button when a target stimulus appears on the computer screen and withhold from pressing a button when a non-target stimulus appears. The participant wears a headband with attached reflective marker, which is tracked by an infra-red camera to measure hyperactivity. Multiple versions of the QbTest exist^11^: QbMini (for children aged <5), QbTest 6–12 (for children aged 6–12 years), QbTest 12–60 (for people aged 12–60 years), QbCheck (remote version conducted via webcam) and QbMT (smartphone version in development). The National Institute for Health and Care Excellence (NICE) has recommended the use of the QbTest (6–12 or 12–60, depending on the age of the patient) alongside clinician judgement to support ADHD diagnosis in 6–17 years old in England and Wales.^15^

Previous systematic reviews of the QbTest have suggested that, when used in support of clinical assessment, the test may improve the efficiency of the ADHD diagnostic process^11^,^13^ and may aid in treatment monitoring.^14^ These reviews have summarised quantitative data only and primarily focus on the accuracy of the QbTest. The present mixed-methods review aims to explore patient, carer and clinician experiences of the QbTest and its impact on patient outcomes for ADHD diagnosis and medication management. This is the first systematic review to integrate qualitative and quantitative data concerning the QbTest, and it improves on previous reviews in this area by involving individuals with lived experience of ADHD in the review team.

## Methods

This review was conducted in line with published systematic review guidance and is reported according to Preferred Reporting Items for Systematic Reviews and Meta-Analyses (PRISMA-2020) (see online supplemental appendix 1).^16 17^ The protocol was registered on PROSPERO (CRD42023482963). This review formed part of a Diagnostic Assessment Report that had a broader scope and was conducted to inform guidance by NICE: an organisation that produces evidence-based guidance on new health technologies for the National Health Service in England and Wales.^18^ We focus on the QbTest because it is the CPT with motion tracker that is currently most commonly used in practice.

### Eligibility criteria

Primary studies that evaluated any version of the QbTest (QbMini <6 years, QbTest 6–12 years, QbTest 12–60 years, QbCheck for remote assessment via webcam, or QbMT smartphone version), alone or in combination with clinical assessment, in a secondary care or a remote setting, were eligible for inclusion. Studies were eligible for inclusion if they made comparisons with other tests or if they reported single-arm results. The comparator test (where used) could be any ADHD diagnostic assessment that did not include the QbTest. Conference abstracts were also eligible for inclusion.

We included studies that enrolled people (any age) who had been referred for the evaluation of suspected ADHD and/or people with an ADHD diagnosis who were undergoing dose titration and initial treatment decisions or being monitored for treatment effectiveness.

We included studies of any design that provided quantitative or qualitative data on any of the following outcomes: time to assessment/diagnostic decision, use of services, impact on clinical decision-making, healthcare professionals’ confidence in assessment, use of interventions, morbidity, mortality, health-related quality of life, cost, ease of use, acceptability to patients, clinicians and clinicians, and patient, carer and clinician experience of the test.

### Search strategy

Studies were identified through searches of MEDLINE, Embase, PsycINFO (Ovid), CINAHL (EBSCO), ClinicalTrials.gov and the WHO ICTRP, from inception to November 2023, with an update search in September 2024. The searches were drawn from our broader Diagnostic Assessment Report conducted for NICE. Searches were not limited by date of publication, language of publication or by study design or reference type. We also reviewed references of included reports, studies included in identified reviews, information submitted by Qbtech (test manufacturer)^18^ and the Qbtech website.^19^ The search strategy is reported in online supplemental appendix 2.

### Study selection, data extraction and quality assessment

Two reviewers independently screened titles and abstracts of records identified by the searches. All reports considered potentially relevant were obtained in full and assessed independently by two reviewers for inclusion. One reviewer extracted data prespecified in the protocol using piloted forms in Microsoft Access and Microsoft Word. We extracted data on Progress-Plus characteristics (factors understood to influence health opportunities and outcomes), where reported.^20^ All data items are provided in online supplemental material. One reviewer assessed study quality using Risk of Bias 2 tool (RoB2) for randomised controlled trials (RCTs),^21^ Risk of Bias in Non-randomised Studies of Interventions (ROBINS-I) for before-and-after studies,^22^ Q-SSP for studies that contributed survey data^23^ and an amended version of the CASP checklist for studies that contributed qualitative data (we excluded question 10 ‘how valuable is the research?’ as this is a critical appraisal judgement rather than an assessment of risk of bias).^24^ Important confounders for consideration in the ROBINS-I assessment were age at the point of seeking ADHD referral, sex, comorbidities, nature and severity of symptoms at presentation, socioeconomic status and ethnicity. A second reviewer checked data extraction and risk of bias assessments. Any disagreements were resolved through discussion or by consulting a third reviewer.

### Data synthesis

One reviewer extracted themes from the studies that had a qualitative component, organised them into conceptual categories, and extracted direct quotes to evidence the synthesised themes.^25^ Where conflicted information, or negative cases, were identified, these were pursued further to enhance methodological rigour. This was checked by a second reviewer.

In line with the convergent-integrated approach to mixed-methods synthesis,^17^ we then ‘qualitised’ the extracted quantitative data, that is, we converted it into textual descriptions to facilitate integration with the qualitative data. We integrated the qualitised data within the thematic structure.

We provide a narrative synthesis of study characteristics and risk of bias assessments, and a narrative synthesis of our integrated findings. We had planned to conduct subgroup analyses, where data were available, to determine whether results differed according to age, sex, ethnicity, people with mental health, behavioural and neurodevelopmental conditions, people with developmental trauma, people in the Youth Justice System or Adult Criminal Justice System, and looked-after children. However, no data were available stratified by these subgroups.

### Patient and public involvement

Five interest holders, including three patient representatives and two clinicians, were involved in this review. Their involvement is reported using the ACTIVE framework in online supplemental appendix 3.^26^ Two of the patient representatives have ADHD and one has a child with ADHD. One clinician works in adult ADHD services and one works in child and adolescent ADHD services. They were recruited using invitations from known contacts. They contributed to two 1.5-hour online team meetings, one during the data extraction stage and one in the analysis phase, to discuss the interpretation and presentation of results. One patient representative (AO-S) is a qualitative researcher, who supervised the qualitative synthesis. All five interest holders were in contact with the researchers throughout the review and responded to questions via email and during meetings. They were invited to review this paper as coauthors—three accepted and are included as coauthors.

### Differences between protocol and review

The protocol was developed for our original NIHR-funded review which had a broader scope and informed NICE guidance.^18^ In this review, we focus only on the QbTest as it is the most researched CPT with motion tracker, and it is most likely to be implemented in practice. We also focus only on patient, carer and clinician experiences of the QbTest and its impact on patient outcomes for ADHD diagnosis and medication management. Data pertaining to test accuracy and test failure rate will be published separately.

## Results

### Search results

**Figure 1:**
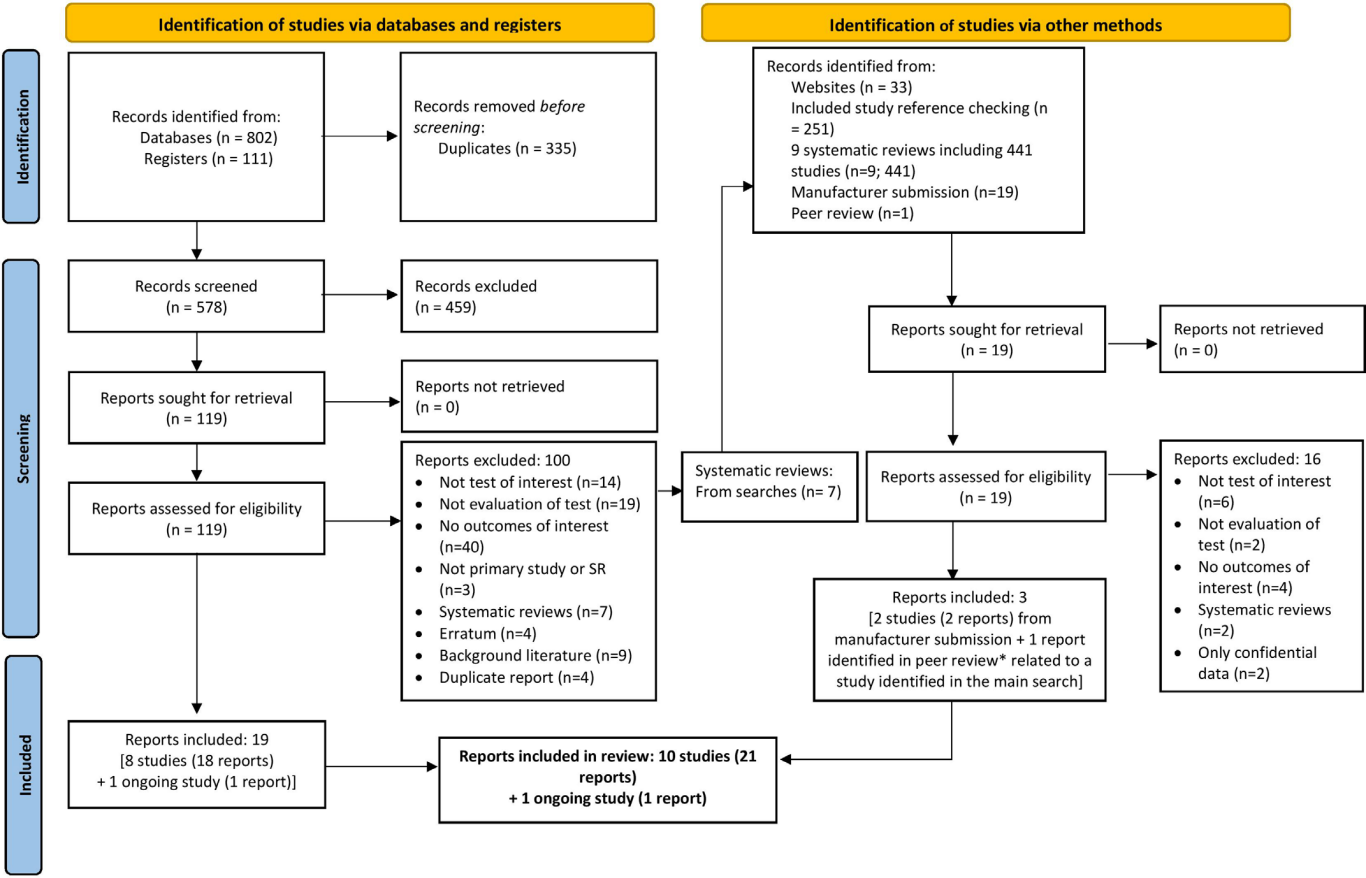
Study selection process. *One report of the Focus ADHD trial (published after review search date; no new trial data) was identified in peer review and incorporated into the review. ADHD, attention deficit hyperactivity disorder.

Searches of bibliographic databases and trial registries identified 578 unique reports. Additional methods of study identification identified 745 reports (figure 1). We included 10 studies (21 reports), in addition to 1 ongoing study (1 report), with no reported results. The ongoing study aims to explore the validity of the QbMT (smartphone version of the QbTest) and was reported only in a trial registry with limited information.^27^ Details of included studies, the ongoing study and studies excluded at full-text screening are provided in online supplemental appendix 4.

### Characteristics of included studies

Table 1 presents an overview of the characteristics of the 10 studies that provided data for this review. Nine studies evaluated the QbTest (6–12 or 12–60, depending on age) in addition to standard clinical assessment in children and adolescents (aged <18 years). One of these was conducted in Ireland,^28^ all others were conducted in the UK. The final study evaluated the QbCheck in adolescents and adults in Germany, Sweden and the USA.^29^ As outlined in table 1, six studies reported both quantitative and qualitative data^28^; the other four studies reported quantitative data. Seven studies enrolled participants with suspected ADHD,^2830^,^36^ two enrolled participants with an ADHD diagnosis^37 38^ and one study (survey of patients from a diagnostic test accuracy study who had used the QbCheck) enrolled 59 participants with ADHD and 69 ‘healthy’ controls.^29^ Further details, including definitions of standard clinical assessment from each study, are provided in online supplemental appendix 5.

**Table 1:** Characteristics of included studies

Author and year	Study design and location	Study component	Details
Chitsabesan *et al*^30^ (2022)	‘FACT’ single-centre feasibility RCT, with embedded qualitative and survey components; UK	RCT	Population: Boys aged 15–18 years from a young offenders institute who had any ADHD symptom from the Comprehensive Health Assessment ToolGroup 1: QbTest+standard assessment (n=30 randomised; 20 completed test)Group 2: Standard assessment (n=30)
		Survey	Population: Boys from FACT trial QbTest+standard assessment group (n=10)Data collection: 12-item ‘QbTest Opinion Questionnaire’ about experience of using QbTest
		Interview	Population: Boys from the QbTest+standard assessment group (n=6) and staff who used QbTest in FACT (n=6)Data collection: Semistructured interviews
Hall *et al*^38^ (2016)	Uncontrolled before-after implementation study; UK	Before-and-after study	Population: Children and adolescents aged 4.5–14.6 years with ADHD diagnosis from community paediatric clinicGroup 1: QbTest+standard assessment (n=40)Group 2: Standard assessment (n=40)
Hollis *et al*^31^ (2018) and Hall *et al*^32^ (2017)	‘AQUA’ RCT, with embedded qualitative and survey components; UK	RCT	Population: Children and adolescents aged 6–17 years referred for first ADHD assessment in child and adolescent mental health services (CAMHS) or community paediatric clinicsGroup 1: QbTest with results available to clinician+standard assessment (‘QbOpen’) (n=123)Group 2: Standard assessment plus QbTest with results withheld from clinician (‘QbBlind’) (n=127)
		Survey	Population: Clinical leads (n=10) from each site and families (n=76) who used QbTest in trialData collection: Survey with questions to clinicians about administering the QbTest and understanding and communicating results. Questions to families focused on the utility of QbTest in understanding symptoms and decisions, and experience of completing test
		Interview	Population: Clinical leads (n=10) from each site and families from ‘QbOpen’ group (n=20)Data collection: Semistructured interviews
Humphreys and Sitton-Kent^33^ (2018)	Uncontrolled before-and-after implementation study, with survey component; UK	Before-and-after study	Population: Children and adolescents aged 5–16 years referred for ADHD assessment in 8 community paediatric mental health settings in 3 National Health Service trustsGroup 1: QbTest+standard assessment (n=unclear)Group 2: Standard assessment (n=unclear)
		Survey	Population: Children/families (n=48) and staff (n=unclear) who used QbTest in studyData collection: Same survey used in the AQUA trial (outlined above)
Hall *et al*^34^ (2022)	‘Focus ADHD’ uncontrolled before-and-after implementation study, with qualitative and survey components; UK	Before-and-after study	Population: Children aged 6–18 years referred for ADHD assessment in 20 CAMHS and paediatric sitesGroup 1: QbTest+standard assessment (n=549)Group 2: Standard assessment (n=549)
		Survey	Population: Children (and parents) who had been assessed with the QbTest (n=22) and healthcare professionals from Focus ADHD (n=65)Data collection: Surveys based on those used in AQUA
		Interview	Population: Healthcare staff who used QbTest in Focus ADHD study (n=21)Data collection: Interviews
Pellegrini *et al*^28^ (2020)	Mixed-methods study of real-world impact of test implementation; Ireland	Survey	Population: Clinicians (n=17), young people (n=15) and their parents/guardians (n=18) who used QbTest in one of three CAMHS from studyData collection: Surveys based on those used in AQUA
		Focus groups	Population: Clinicians who used QbTest in one of three CAMHS teams from study (n=19)Data collection: Focus Groups
Sharma *et al*^35^ (2022)	Uncontrolled before-and-after implementation study; UK	Before-and-after study	Population: Children (mean age 11.7 years) referred for ADHD/non-specific behavioural problems/autism spectrum disorder, assessed for ADHD in hospital paediatric clinicGroup 1: QbTest+standard assessment (n=20)Group 2: Standard assessment (n=20)
Ulberstad *et al*^29^ (2020)	Two-gate test accuracy study with survey component; Germany, Sweden, USA	Survey	Population: Adolescents/adults who used QbCheck in the test accuracy study (n=125; age not reported; 59 ADHD, 69 healthy controls)Data collection: QbCheck user-friendliness survey— three questions answered on a scale from 0 to 10 that assessed the usability of the test; one yes/no question about problems with using the test
Vogt and Shameli^36^ (2011)	Uncontrolled before-and-after implementation study; UK	Before-and-after study	Population: Children and adolescents (QbTest Group mean age 9 years; non-QbTest group mean age 10.5 years) referred for ADHD assessment in CAMHSGroup 1: QbTest+standard assessment (n=62)Group 2: Standard assessment (n=46)
Williams *et al*^37^ (2021)	‘QUOTA’ parallel group, single-blind, multi-site, feasibility RCT, with embedded qualitative and survey components; UK	RCT	Population: Children aged 6–15 years with ADHD diagnosis, starting ADHD medication and referred to CAMHS/ community paediatric clinicsGroup 1: QbTest+usual care (n=21)Group 2: Standard assessment (n=23)
		Survey	Population: Survey to clinicians (n=unclear) who used QbTest in QUOTAData collection: Clinician pro forma to record data on diagnoses and clinical decision-making along with resources used to reach decisions.
		Interview	Population: Parents of eight children from QUOTA trial (n=unclear) and clinicians from QUOTA trial (n=5)Data collection: Interviews

ADHD, attention deficit hyperactivity disorder; AQUA, assessing QbTest utility in ADHD ; CAMHS, child and adolescent mental health services; QUOTA, QbTest utility for optimising treatment in ADHD; RCT, randomised controlled trial.

Three studies were RCTs with embedded survey and interview components. The Assessing QbTest Utility in ADHD (AQUA) trial was a full RCT, in which both groups received the QbTest in addition to standard assessment. However, in one group the QbTest results were available to the clinician, while in the other group, these were withheld.^31^ Surveys and qualitative interviews were conducted with clinical leads and families who used the QbTest in the AQUA trial.^32^ The other two studies were small feasibility RCTs.^30 37^ One assessed the feasibility of the QbTest for the assessment of ADHD in boys in prison (named "FACT"). Boys who had used the QbTest were surveyed and both boys and staff were interviewed. In the QbTest Utility for Optimising Treatment in ADHD (QUOTA) feasibility trial, clinicians were surveyed and both clinicians and parents were interviewed.

Five before-and-after studies analysed clinical records from before and after the implementation of the QbTest as part of the diagnostic workup for children—one was restricted to children with a diagnosis of ADHD^38^ and the others selected children with suspected ADHD.^33^,^36^ One of these studies included a survey of children and families,^33^ and one (Focus ADHD) included both a survey of children and families and interviews with healthcare staff.^34^

The final two studies were: a study of real-world QbTest implementation that involved a survey to clinicians, young people and their carers and focus groups with clinicians^28^ and a diagnostic test accuracy study that included a brief survey of those who used the QbCheck to assess user-friendliness.^29^

Studies reported on few Progress-Plus characteristics.^20^ Three studies reported sex: participants were predominantly male and one study (FACT) only enrolled boys.^30 31 38^ Three studies reported ethnicity: most participants were white.^30 31 37^ One study (FACT) reported on education and time-dependent relationships: most participants reported no education and all were in a young offenders institute.^30^ Three studies reported on neurodevelopmental/learning disorders: comorbidities were common.^31 37 38^ Eight studies reported age: participants were aged <18. No studies reported on the other Progress-Plus characteristics (place of residence, religion, socioeconomic status, social capital or occupation). Little demographic information was reported for the samples surveyed and interviewed (see online supplemental appendix 5), and results were not stratified by Progress-Plus characteristics.

### Quality assessment

#### Randomised controlled trials

The AQUA trial was judged at high risk of bias for time-to-event outcomes: number of days to diagnostic decision, number of clinic appointments until diagnosis, number of consultations to diagnostic decision, number of minutes spent at clinic appointments until diagnosis. This was due to concerns regarding missing outcome data (see online supplemental appendix 5).^31^ The outcome cost of appointments was judged at an unclear risk of bias as it was not clear how costs were calculated. Other outcomes were judged at low risk of bias: diagnostic confidence and stability of diagnosis, proportion of participants with a diagnostic decision, diagnostic status. Risk of bias was not formally assessed for the FACT^30^ and QUOTA^37^ feasibility trials because they were designed and powered to assess the feasibility of conducting a full trial rather than to formally evaluate impact on outcomes.^39^

#### Before-and-after studies

All five implementation studies were judged at serious risk of bias. In four of these, this was because important confounders (ie, age at the point of seeking ADHD referral, sex, comorbidities, nature and severity of symptoms at presentation, socioeconomic status and ethnicity) were not controlled for (online supplemental appendix 5).^33 34 36 38^ This included the Focus ADHD study, for which an additional important potential confounder was the COVID-19 pandemic. The remaining study was reported only as a conference abstract, with limited detail.^35^ This study was judged as ‘no information’ for the confounding domain, but at serious risk of bias due to selection of participants into the study, as participants were excluded if they had an inconclusive diagnosis or no diagnosis within the study time frame.

#### Survey study components

Based on assessment with the Q-SSP checklist, the survey component of the AQUA sub-study^32^ was judged as having few concerns with quality (online supplemental appendix 5). The other studies were judged to have some concerns due to limited information reported on methodology, analysis and participants.^28^,^3033 34 37^

#### Qualitative study components

Based on assessment with the CASP checklist, the AQUA substudy qualitative component^32^ and the study undertaken in Ireland^28^ were judged as having no concerns regarding study quality (online supplemental appendix 5). The qualitative component of the QUOTA trial also yielded few quality concerns.^37^ The other two studies appeared to have used appropriate methodology, but they reported insufficient detail which made it difficult to assess some CASP checklist items.^30 34^

### Thematic findings

We organised themes emerging from the qualitative synthesis into two broad conceptual categories: (1) views around the helpfulness of the QbTest (contribution to ADHD diagnosis, treatment decision-making and communication with caregivers) and (2) barriers to the implementation of the QbTest (practical barriers and the acceptability of the QbTest to patients and caregivers) (see table 2).

**Table 2:** Overview of themes and the number of studies providing data for each theme

Conceptual category	Theme	Subtheme	No. of studies providing qualitative data	No. of studies providing quantitative data
Views around the helpfulness of the QbTest	Contribution to ADHD diagnosis	Time to diagnostic decision	4	5
		Proportion of patients with a diagnostic decision	0	2
		Clinician confidence in the diagnostic decision	3	1
		Diagnosis in complex cases	3	1
		Cost	0	2
	Treatment decision-making	No subthemes identified	5	3
	Communication with caregivers	No subthemes identified	3	4
Barriers to the implementation of the QbTest	Practical barriers	Scheduling	1	0
		Space	4	0
		Staffing	4	0
		Technology	4	0
	Acceptability of the QbTest to patients and caregivers	No subthemes identified	3	4

ADHD, attention deficit hyperactivity disorder.

We identified quantitative data pertaining to the following pre-specified outcomes: time to assessment/diagnostic decision, use of services, impact on clinical decision-making, healthcare professionals’ confidence in assessment, cost, ease of use, acceptability to patients and clinicians, and patient, carer or clinician experience of the test (see online supplemental appendix 5). We identified no quantitative data concerning use of interventions, morbidity, mortality or health-related quality of life. We do not include quantitative patient outcome data from the FACT and QUOTA feasibility RCT studies, because, as noted, they were not designed or powered to evaluate impact on outcomes.^39^

Our integrated synthesis of the qualitative data and the ‘qualitised’ quantitative data is presented below. Findings should be interpreted with caution due to the aforementioned concerns with risk of bias. Findings should also be interpreted within the context of the study characteristics outlined in table 1. Nine of the 10 included studies enrolled children and young people, and studies did not stratify by key subgroups (eg, age, sex, ethnicity and comorbidities). Illustrative quotes are included throughout and all extracted data are provided in online supplemental appendix 5.

### Views around helpfulness of the test

#### Contribution to ADHD diagnosis

Within this theme, we identified evidence concerning the impact of the QbTest on the time to diagnostic decision, the proportion of patients with a diagnostic decision, clinician confidence in the diagnostic decision, diagnosis in complex cases and cost.

Qualitative (four studies) and quantitative evidence (five studies) suggested that the QbTest can reduce the time it takes to reach an ADHD diagnostic decision. In qualitative interviews, healthcare professionals reported that the QbTest reduced the time to diagnosis and it can streamline the diagnostic process,^28 30 32 34^ although they stated a need to determine where it should take place on the diagnostic pathway.^32 34^ Families agreed that the QbTest might help to shorten the emotionally overwhelming diagnostic process and to facilitate treatment initiation but emphasised that the process should not be rushed, and that a child should not be quickly ‘labelled’.^32^ Some healthcare professionals felt that time and cost savings could happen by replacing school observations with the QbTest, and that its use may help to improve waiting times.^32^ One before-and-after study did replace school observations with the QbTest in some cases, and healthcare professionals reported that this led to children having fewer appointments and getting educational support more quickly.^34^

“*I see it as a way of reducing the amount of time children are waiting to be seen. And thus, reducing the number of follow-ups, thus reducing the number of times they have to come back to the hospital so it’s an opportunity to save the patients and parents time*.”^34^ *- Healthcare professiona*l

“*I just wished it were more like I say I was in and out, just wished it were more appointments and a bit more time.”*^32^ *– Parent*

Quantitative evidence from the AQUA RCT and four before-and-after studies supported these findings. The QbTest reduced the time^31 33 35^ and the number of consultations^3133^,^35 38^ required to reach a diagnostic decision (see online supplemental appendix 5). Survey data from one before-and-after study also suggested that some patients and carers felt that the QbTest helped to speed up the assessment process and to get a diagnosis.^34^

Quantitative evidence from two studies was mixed as to whether the QbTest increases the proportion of patients with a diagnostic decision. The AQUA RCT reported that, at 6 months, the proportion of patients with a diagnostic decision was higher, and a diagnosis of ADHD was ruled out in more people, in the QbTest group than the control group. There was no difference in the stability of the diagnosis over time. The Focus ADHD study reported that fewer children were diagnosed with ADHD post-QbTest implementation than pre-QbTest implementation. However, the post-QbTest phase coincided with the COVID-19 pandemic, which is likely to have confounded the results.

Qualitative (three studies) and quantitative (one study) evidence suggested that the QbTest may increase clinician confidence in the diagnostic decision. Qualitative interview data from clinicians and healthcare staff in three studies suggested increased clinician confidence in the diagnostic decision when using the QbTest.^28 32 34^ In one of these studies (Focus ADHD), healthcare staff commented that increased confidence was derived from the perceived objectivity of the test,^34^ a factor that was also raised as a positive feature of the test in three other studies.^28 30 32^ Quantitative data from the AQUA RCT supported these findings, as clinician confidence in the diagnostic decision was increased in the QbTest group, compared with the control group.

*“I would move to the diagnosis more confidently and more quickly having evidence that something was wrong, you know objective evidence…reduced the amount of the anxiety of uncertainty.”* ^32^ *– Healthcare professional*

Qualitative (three studies) and quantitative evidence (one study) suggested healthcare staff value the role of the QbTest for the diagnosis of ADHD in complex cases. In qualitative interviews, healthcare staff noted that the QbTest supported them to identify individuals with subtle presentation (eg, girls who often internalise symptoms)^32 34^ and helped the diagnostic process when patients have comorbidities,^30 32^ or when there is contradictory information reported between home and school settings.^34^ Survey data from one of these studies also suggested that healthcare professionals felt that the QbTest was most useful for decision making in certain groups, for example, in cases where the parent or school does not agree with the clinician’s decision, in identifying patients for autism spectrum disorder assessment by being able to rule out ADHD, and in those with subtle presentations.^34^

*“I think it works well with subtle presentations. Presentations maybe where there’s a disagreement between school and home. Cases where there are parental disagreements. Cases where young people themselves are unsure.”* ^34^ *– Healthcare professional*

Quantitative evidence (two studies) suggested that the use of the QbTest may reduce the cost of clinic appointments. The AQUA trial reported that the cost of clinic appointments was slightly less in the QbTest group compared with the control group (£87.62 vs £90.06) but did not provide information on how these costs were estimated or account for the cost of QbTest itself. Additionally, one before-and-after study reported that the average cost per patient for a diagnosis was £329.40 pre-QbTest implementation and £265.90 post-QbTest implementation. These studies did not report a formal between-group comparison.

#### Treatment decision-making

This theme identified evidence concerning the impact of the QbTest on the ADHD treatment decision-making process. Qualitative (five studies) and quantitative (three studies) evidence mostly suggested that the QbTest can support treatment decision-making. In qualitative interviews, healthcare staff and families appeared to value the role of the QbTest for dose titration, checking medication utility and improving medication adherence.^3032^,^34 37^ Clinicians reported that they appreciated the objectivity of the QbTest in comparison to traditional measures for medication monitoring, particularly for complex patients.^37^ Clinicians also reported that being able to directly observe medication effects with the QbTest led to greater parental acceptance regarding treatment recommendations and initiating medication,^37^ greater adherence to medication^32^ and increased parental confidence in treatment decision making.^37^ Families interviewed in the AQUA trial reported that seeing the QbTest results made them more confident that the medication would help their child and helped them to better understand treatment impact.^32^

*“It’s a big decision to allow your children to have these drugs, as it were. So, again, seeing those results made me more confident that yes the medication would help him”* ^32^ *– Parent*

*“They can see there is a difficulty there and that the medication can improve that, I think it does really improve adherence and understanding of what the difficulties are for the kids more than anybody else”* ^32^ *- Healthcare professional*

Survey data mostly supported these findings. In two studies, most healthcare professionals felt that the QbTest increased their confidence in decision making about treatment^34 37^ and helped them to measure medication effectiveness.^34^ However, in a survey of clinicians in CAMHS (sample size not reported), only 30% of respondents agreed that the QbTest results influenced treatment decisions (60% of respondents remained neutral and 10% strongly disagreed).^33^ Additionally, survey data from the AQUA and FACT trials suggested that patients and caregivers were not convinced that the results of the QbTest helped them to understand medication decisions, contrasting with the qualitative interviews from the AQUA trial.^30 32^ It is possible that this contrast may be because the FACT trial was conducted in the specific population of boys in a young offenders institute, and in the AQUA trial, most of the survey respondents did not commence medication, making the results difficult to interpret.

One before-and-after implementation study reported no difference between groups in the proportion of children in each of the following categories at 1-year follow-up: trialling medication, continuing on medication, discontinued medication, ADHD diagnosis changed and lost to follow-up. It was reported that 37% of the group assessed without the QbTest, compared with none in the group assessed with the QbTest, were given a revised diagnosis of ADHD at 1-year follow-up, after having been diagnosed as not having ADHD initially.^36^

#### Communication with caregivers

This theme reports the impact of the QbTest on communication between healthcare professionals and caregivers concerning ADHD diagnosis. Qualitative (three studies) and quantitative (four studies) evidence suggested that clinicians felt the QbTest had a positive impact on communication with caregivers, while some patients and carers seemed less convinced. Clinicians reported in qualitative interviews that the QbTest has the potential to improve communication between clinicians and patients and their families,^28 32 34^ between clinicians and schools,^32^ between clinical colleagues,^28^ and between patients and families.^32^ Some clinicians appreciated that they could show families a comparison of the child’s performance to a normative sample and felt this helped them to communicate the diagnostic decision and helped families to accept it.^32^ However, some clinicians felt that families can still struggle to accept the decision.^34^

*“A lot of parents who previously would have probably shouted and screamed at you for not saying their child had ADHD will accept it if the computer is not showing the evidence”* ^32^ *– Healthcare professional*

Only one study (AQUA) included interviews with families on this topic, and most families reported that they felt that clinicians explained the QbTest results well. However, some felt that it was unclear how the QbTest report was being used to inform decision making.^32^

*“I don’t know if she explained, it felt like the QbTest had said it so that’s what we’re going with”*^32^ – Parent

In line with qualitative findings, survey data also suggested that healthcare professionals felt that the QbTest helped to improve communication of a diagnostic decision with the patient.^2832^,^34^ However, patients and carers were less convinced that the results of the QbTest helped them to understand diagnostic decisions, medication decisions or to understand their symptoms better.^30 32 34^ This may be due to poor communication, as some survey respondents reported that they did not find the test helpful because the results were not properly explained to them.^34^ In two studies, the majority of survey respondents reported that the clinician talking through the test results helped them to understand how their diagnosis had been made.^28 33^

### Barriers to the implementation of the test

#### Practical barriers

This theme focuses on practical barriers to QbTest implementation, including scheduling, space, staffing and technology. Qualitative evidence (five studies) reported barriers to QbTest implementation.^28 30 32 34 37^ One study highlighted potential scheduling issues caused by the need for more appointments for medication management.^37^ Four studies reported concerns with room requirements for conducting the QbTest. For example, it was reported that a room is required to be able to administer the QbTest, and sometimes this is hard to arrange, which means the equipment may need to be moved between rooms.^30 32 34^ Focus groups with clinicians in CAMHS also highlighted concerns about managing environmental factors influencing the QbTest, as the test needs to be conducted in a quiet environment.^28^

*“The main [challenges] were just the practical side, like the room space and things. It’s really competitive to get rooms here so making sure it was booked well in advance.”* ^34^ *– Healthcare professional*

Four studies highlighted staffing as a potential barrier, as they noted that a trained staff member must be available to conduct the QbTest.^28 30 32 34^ Additionally, four studies reported technology as a barrier, including issues with accessing laptops, sharing passwords, internet connection and printer access.^28 30 32 34^

*“There were a lot of IT [Information Technology] governance issues to get it set up”* ^32^ *- Healthcare professional*

#### Acceptability of the QbTest to patients and caregivers

This theme included evidence concerning the acceptability of the QbTest to patients and caregivers. Qualitative (three studies) and quantitative (four studies) evidence was mixed concerning the acceptability of the QbTest to patients and caregivers. Some patients reported in qualitative interviews that the test was boring, long and repetitive (no quotes published).^30 37^ Healthcare staff interviewed in the Focus ADHD study also noted that some people (particularly young people and individuals with autism spectrum disorder) experienced sensory discomfort during the test, and they struggled with the need to wear the tight headband that measures hyperactivity.^34^ The staff highlighted that some found it difficult to follow the instructions and experienced anxiety during the test. They noted this could be due to the content of the test, the use of the word ‘test’ and/or because they were separated from their caregivers. A lack of representation of different ethnicities in the test explanation video and the need to specify biological sex before starting the test were also flagged as concerns by staff. Patients were not interviewed about their perception of the test in this study.

*“A lot of our young people that come in for both an autism and an ADHD assessment can experience difficulty with the plastic covering of the headband, because it’s quite a sensory thing on the head and that can be quite uncomfortable.”* ^34^ *– Healthcare professional*

In surveys, some participants reported that the QbTest was difficult to complete, as it was too long and stressful.^30^ However, others did not report issues with it.^28 33^ The one study that briefly surveyed patients about the user friendliness of the QbCheck reported that they found it easy to use, including test preparation and understanding and following test rules.^29^

## Discussion

### Summary of main findings

This mixed-methods systematic review identified 10 studies that evaluated the QbTest (9 QbTest; 1 QbCheck; none QbMini) for ADHD diagnosis or medication management. We also identified one ongoing study, reported in a trial registry with limited information and no results, that aims to validate the QbMT (smartphone version of the test in development). The included studies were mostly conducted in children/adolescents in the UK, and those that reported sex and ethnicity mainly enrolled white males. Many of the included studies were judged at high risk of bias, due to issues with missing outcome data, confounding or insufficient reporting, so results should be interpreted with caution. Qualitative and quantitative findings largely supported each other and suggested that, in children and young people, the addition of the QbTest may reduce time to diagnosis, increase the proportion of patients with a diagnostic decision, improve clinician confidence in the diagnostic decision, support the diagnosis of complex cases and treatment decision-making, reduce cost of clinic appointments and improve communication with caregivers. Overall, the QbTest appeared to be generally well received by clinicians, patients and carers. However, reported barriers to its use included staffing, technology, the length and repetitive nature of the test, and potential sensory discomfort and anxiety for some individuals. We identified little data on the impact of the QbTest on patient outcomes, or regarding its use in medication management.

### Implications

Our findings are in line with those of previous quantitative reviews concerning the clinical utility of the QbTest for ADHD assessment^12 13^ and have informed NICE’s recent decision to recommend the use of the QbTest in addition to clinician judgement to support ADHD diagnosis in 6–17 years old in England and Wales.^15^ This has important implications for clinicians, patients and carers. Clinicians require infrastructure to be in place before the QbTest can be implemented, for example, a quiet room with a computer, time to be able to administer the test and training to support the effective completion of the test and communication of test results. The positioning of the QbTest on the diagnostic pathway also needs to be carefully considered and agreed, to reduce variation in the potential impact of the test across healthcare services. The use of the QbTest may free up some clinician time, by aiding the corroboration of accounts from informants and reducing time-consuming appeals. Our patient representatives are hopeful that this would, in turn, have positive implications for patients, by improving waiting lists, reducing the difficult and lengthy diagnostic process and contributing to more timely treatment. While medication is not the only treatment option for those with ADHD, it is worth noting that shortages of ADHD medication have been reported, due to increased global demand and issues with manufacturing.^40^ The implementation of the QbTest may further impact on the availability of ADHD medication and other ADHD management strategies (eg, behavioural interventions, educational and psychosocial support) by exposing unmet need for treatment.

### Strengths and limitations

Strengths of this systematic review include that it is the first to integrate quantitative and qualitative evidence concerning the impact of the QbTest on ADHD diagnosis and medication management, and the acceptability of the test to patients, carers and clinicians. We used a mixed-methods approach to add greater depth beyond previous reviews that have focused solely on quantitative data (mostly concerning test accuracy)^12^,^14^ and to maximise the helpfulness of the evidence to decision-makers. We included the perspective of patients, both within the findings of the synthesis and from involving individuals with lived experience of ADHD in the review team. We also considered factors that contribute to health inequity, by extracting available data on Progress-Plus characteristics.^20^ We used robust and transparent methodology and reported the review as per PRISMA-2020.^16^ Limitations mainly relate to the evidence base. We only identified one full RCT (AQUA) and overall many of the included studies were judged at high risk of bias. Additionally, most studies were conducted in children (predominantly white males in the studies that reported sex and/or ethnicity) within the UK. It is, therefore, not clear whether results would be similar in adults, in other countries, or in subgroups, such as people with comorbidities or females who tend to mask symptoms.^41^ Additionally, good quality evidence is lacking on the use of the QbTest for medication selection, dose titration, long-term treatment monitoring and in people for whom current ADHD assessment cannot reach a diagnosis.

### Suggestions for future research

There are a number of areas where further research is needed. Well-designed RCTs with qualitative substudies are required to evaluate the use of the QbTest (all versions) for medication management, as there is currently very limited evidence in this area. RCTs to explore the impact of the QbTest on ADHD diagnosis and medication management in adults is also a priority. Future RCTs should be powered to explore the impact of the QbTest in subgroups of patients including age, sex, ethnicity and people with comorbidities. Studies should measure patient outcomes and patient, carer and clinician experience of using the test. They should prioritise outcomes that were shown to be important to patients in the qualitative evaluations (eg, communication, understanding and acceptance of diagnostic decision, acceptability of the test to patients). Ideally, the studies would also include a direct comparison to other CPTs with motion trackers, such as Nesplora Aquarium, Nesplora AULA and the EFSim Test.^42 43^ These CPTs use a virtual reality headset and, therefore, may be more fun to complete; however, there is currently very little evidence available on them.^18^ Further costs data are also needed. A recent review found that the use of the QbTest in addition to clinical assessment is likely to be cost effective; however, it was restricted by data limitations.^18^ Therefore, future studies should also conduct economic analyses, with long-term cost data, that includes costs for education and healthcare settings.

## Conclusions

In conclusion, the available evidence suggests that the QbTest may improve the ADHD diagnostic process, when used alongside clinical assessment in children and young people. However, well-designed RCTs with qualitative substudies are required to assess the impact of the QbTest on patient outcomes, user experience and cost. RCTs that focus on medication management and enrol adults are particularly needed. Such RCTs should include economic analyses and explore the impact of the test in subgroups including age, sex, ethnicity and comorbidities.

## Supplementary material

10.1136/bmjopen-2024-095479 online supplemental appendix 1

## Data Availability

All data relevant to the study are included in the article or uploaded as supplementary information.
