# Supplementary material for: QbTest for ADHD assessment and medication management: a mixed-methods systematic review of impact on clinical outcomes and patient, carer and clinician experiences
Source: BMJ Open. 2025 Apr 17;15(4):e095479. doi: 10.1136/bmjopen-2024-095479 (PMC12007030; doi:10.1136/bmjopen-2024-095479)
Supplement: online supplemental appendix 1 [file bmjopen-15-4-s001.docx]

Supplementary material

# Appendix 1: PRISMA 2020 Checklist

| **Section and Topic** | **Item #** | **Checklist item** | **Location where item is reported** |
| --- | --- | --- | --- |
| **TITLE** | | |  |
| Title | 1 | Identify the report as a systematic review. | Title page |
| **ABSTRACT** | | |  |
| Abstract | 2 | See the PRISMA 2020 for Abstracts checklist. | P1 |
| **INTRODUCTION** | | |  |
| Rationale | 3 | Describe the rationale for the review in the context of existing knowledge. | P3 |
| Objectives | 4 | Provide an explicit statement of the objective(s) or question(s) the review addresses. | P3 |
| **METHODS** | | |  |
| Eligibility criteria | 5 | Specify the inclusion and exclusion criteria for the review and how studies were grouped for the syntheses. | P4 |
| Information sources | 6 | Specify all databases, registers, websites, organisations, reference lists and other sources searched or consulted to identify studies. Specify the date when each source was last searched or consulted. | P4 |
| Search strategy | 7 | Present the full search strategies for all databases, registers and websites, including any filters and limits used. | Appendix 2 |
| Selection process | 8 | Specify the methods used to decide whether a study met the inclusion criteria of the review, including how many reviewers screened each record and each report retrieved, whether they worked independently, and if applicable, details of automation tools used in the process. | P4 |
| Data collection process | 9 | Specify the methods used to collect data from reports, including how many reviewers collected data from each report, whether they worked independently, any processes for obtaining or confirming data from study investigators, and if applicable, details of automation tools used in the process. | P4 |
| Data items | 10a | List and define all outcomes for which data were sought. Specify whether all results that were compatible with each outcome domain in each study were sought (e.g. for all measures, time points, analyses), and if not, the methods used to decide which results to collect. | P4 |
|  | 10b | List and define all other variables for which data were sought (e.g. participant and intervention characteristics, funding sources). Describe any assumptions made about any missing or unclear information. | P4/Appendix 5 |
| Study risk of bias assessment | 11 | Specify the methods used to assess risk of bias in the included studies, including details of the tool(s) used, how many reviewers assessed each study and whether they worked independently, and if applicable, details of automation tools used in the process. | P4 |
| Effect measures | 12 | Specify for each outcome the effect measure(s) (e.g. risk ratio, mean difference) used in the synthesis or presentation of results. | Protocol |
| Synthesis methods | 13a | Describe the processes used to decide which studies were eligible for each synthesis (e.g. tabulating the study intervention characteristics and comparing against the planned groups for each synthesis (item #5)). | NA |
|  | 13b | Describe any methods required to prepare the data for presentation or synthesis, such as handling of missing summary statistics, or data conversions. | NA |
|  | 13c | Describe any methods used to tabulate or visually display results of individual studies and syntheses. | P5 |
|  | 13d | Describe any methods used to synthesize results and provide a rationale for the choice(s). If meta-analysis was performed, describe the model(s), method(s) to identify the presence and extent of statistical heterogeneity, and software package(s) used. | P5 |
|  | 13e | Describe any methods used to explore possible causes of heterogeneity among study results (e.g. subgroup analysis, meta-regression). | NA |
|  | 13f | Describe any sensitivity analyses conducted to assess robustness of the synthesized results. | NA |
| Reporting bias assessment | 14 | Describe any methods used to assess risk of bias due to missing results in a synthesis (arising from reporting biases). | NA |
| Certainty assessment | 15 | Describe any methods used to assess certainty (or confidence) in the body of evidence for an outcome. | NA |
| **RESULTS** | | |  |
| Study selection | 16a | Describe the results of the search and selection process, from the number of records identified in the search to the number of studies included in the review, ideally using a flow diagram. | P5/P6 |
|  | 16b | Cite studies that might appear to meet the inclusion criteria, but which were excluded, and explain why they were excluded. | Appendix 4 |
| Study characteristics | 17 | Cite each included study and present its characteristics. | P6-8 |
| Risk of bias in studies | 18 | Present assessments of risk of bias for each included study. | P9-10 |
| Results of individual studies | 19 | For all outcomes, present, for each study: (a) summary statistics for each group (where appropriate) and (b) an effect estimate and its precision (e.g. confidence/credible interval), ideally using structured tables or plots. | NA |
| Results of syntheses | 20a | For each synthesis, briefly summarise the characteristics and risk of bias among contributing studies. | P6-P10 |
|  | 20b | Present results of all statistical syntheses conducted. If meta-analysis was done, present for each the summary estimate and its precision (e.g. confidence/credible interval) and measures of statistical heterogeneity. If comparing groups, describe the direction of the effect. | P10-P14 |
|  | 20c | Present results of all investigations of possible causes of heterogeneity among study results. | NA |
|  | 20d | Present results of all sensitivity analyses conducted to assess the robustness of the synthesized results. | NA |
| Reporting biases | 21 | Present assessments of risk of bias due to missing results (arising from reporting biases) for each synthesis assessed. | NA |
| Certainty of evidence | 22 | Present assessments of certainty (or confidence) in the body of evidence for each outcome assessed. | NA |
| **DISCUSSION** | | |  |
| Discussion | 23a | Provide a general interpretation of the results in the context of other evidence. | P15-16 |
|  | 23b | Discuss any limitations of the evidence included in the review. | P15-16 |
|  | 23c | Discuss any limitations of the review processes used. | P15-16 |
|  | 23d | Discuss implications of the results for practice, policy, and future research. | P15-16 |
| **OTHER INFORMATION** | | |  |
| Registration and protocol | 24a | Provide registration information for the review, including register name and registration number, or state that the review was not registered. | P1 |
|  | 24b | Indicate where the review protocol can be accessed, or state that a protocol was not prepared. | P1/P3 |
|  | 24c | Describe and explain any amendments to information provided at registration or in the protocol. | P5 |
| Support | 25 | Describe sources of financial or non-financial support for the review, and the role of the funders or sponsors in the review. | P17 |
| Competing interests | 26 | Declare any competing interests of review authors. | P17 |
| Availability of data, code and other materials | 27 | Report which of the following are publicly available and where they can be found: template data collection forms; data extracted from included studies; data used for all analyses; analytic code; any other materials used in the review. | P17 |

# Appendix 2: Search strategy

The focus of this manuscript specifically is the Qb Test, but the search strategy is drawn from a systematic review that had a broader scope and included other tests in addition to the QbTest. Studies relating to the other tests identified by this search have been excluded from this manuscript. The search takes a multistranded approach to identify studies reporting by test name or known study IDs. The lines pertinent to Qb Test are Lines 1-2 and known study IDs, lines 9-10. The search is not limited by date of publication, language of publication, or by study design or reference type. This search approach is highly sensitive and fulfils the aims of a mixed-methods review. Below, we report our update search (10 September 2024). Our main search was identical to this update.

| **Resource** | **N** |
| --- | --- |
| MEDLINE (MEDALL) | 27 |
| Embase | 25 |
| PscyINFO | 86 |
| CINAHL | 44 |
| ClinicalTrials.gov | 40 |
| ICTRP | 29 |
| Total | 211 |
| -duplicates | -140 |
| To screen | 71 |

Database: MEDLINE (MEDALL)

Host: Ovid

Data Parameters: 1946 to September 09, 2024

Date of Search: 10 Sep. 24

1 (QbTest* or "Qb Test*" or "(Qb) Test*" or "Qb Mini*" or "QbMini*" or (("Quantified Behavior*" or "Quantified

Behaviour*") adj5 test*) or QbTech).af. (77)

2 (QbCheck* or "Qb Check*" or "(Qb) Check*").af. (1)

3 (Nesplora* or "Giunti psychometrics").af. (22)

4 (ARVO* or EFSim* or "EF Sim*" or EPELI or "Peili Vision Company").af. (1594)

5 Attention Deficit Disorder with Hyperactivity/ or ADHD.af. (46605)

6 4 and 5 (5)

7 ((motion* adj5 senso*) and (hyperactivity or ADHD)).ti,ab,kf. (6)

8 1 or 2 or 3 or 6 or 7 (109)

9 NCT03368573.af. or (QUOTA and adhd).ti,kf. [QB test] (3)

10 NCT02209116.af. or ((AQUA and ADHD) or AQUA2).ti,kf. [QB test] (6)

11 NCT02473185.af. [QB test] (1)

12 NCT02477280.af. [QB test] (0)

13 NCT05846815.af. [ARVO Test] (0)

14 9 or 10 or 11 or 12 or 13 (10)

15 8 or 14 (111)

16 (2023* or 2024*).dt,dp,ed,ep,yr.

17 15 and 16 (27)

Database: Embase

Host: Ovid

Data Parameters: 1974 to 2024 September 09

Date of Search: 10 Sep. 24

# Searches Results

1 (QbTest* or "Qb Test*" or "(Qb) Test*" or "Qb Mini*" or "QbMini*" or (("Quantified Behavior*" or "Quantified Behaviour*") adj5 test*) or QbTech).af. 96

2 (QbCheck* or "Qb Check*" or "(Qb) Check*").af. 3

3 (Nesplora* or "Giunti psychometrics").af. 25

4 (ARVO* or EFSim* or "EF Sim*" or EPELI or "Peili Vision Company").af. 62936

5 attention deficit hyperactivity disorder/ or ADHD.af. 58054

6 4 and 5 21

7 ((motion* adj5 senso*) and (hyperactivity or ADHD)).ti,ab,kf. 10

8 1 or 2 or 3 or 6 or 7 151

9 NCT03368573.af. or (QUOTA and adhd).ti,kf. [QB test] 3

10 NCT02209116.af. or ((AQUA and ADHD) or AQUA2).ti,kf. [QB test] 7

11 NCT02473185.af. [QB test] 1

12 NCT02477280.af. [QB test] 0

13 NCT05846815.af. [ARVO Test] 0

14 9 or 10 or 11 or 12 or 13 11

15 8 or 14 152

16 (2023* or 2024*).yr. 3125110

17 15 and 16 25

Database: PsycINFO

Host: Ovid

Data Parameters: 1806 to September 2024 Week 1

Date of Search: 10 Sep. 24

# Searches Results

1 (QbTest* or "Qb Test*" or "(Qb) Test*" or "Qb Mini*" or "QbMini*" or (("Quantified Behavior*" or "Quantified Behaviour*") adj5 test*) or QbTech).af. 142

2 (QbCheck* or "Qb Check*" or "(Qb) Check*").af. 7

3 (Nesplora* or "Giunti psychometrics").af. 842

4 (ARVO* or EFSim* or "EF Sim*" or EPELI or "Peili Vision Company").af. 5577

5 Attention Deficit Disorder with Hyperactivity/ or ADHD.af. 97719

6 4 and 5 55

7 ((motion* adj5 senso*) and (hyperactivity or ADHD)).ti,ab. 3

8 1 or 2 or 3 or 6 or 7 1043

9 NCT03368573.af. or (QUOTA and adhd).ti. [QB test] 0

10 NCT02209116.af. or ((AQUA and ADHD) or AQUA2).ti. [QB test] 2

11 NCT02473185.af. [QB test] 0

12 NCT02477280.af. [QB test] 0

13 NCT05846815.af. [ARVO Test] 0

14 9 or 10 or 11 or 12 or 13 2

15 8 or 14 1043

16 (2023* or 2024*).yr. 255925

17 15 and 16 86

Database: CINAHL

Host: EBSCOhost

Data Parameters: 1908-current

Date of Search: 10 Sep. 24

| **#** | **Query** | **Results** |
| --- | --- | --- |
| S9 | S1 OR S2 OR S3 OR S6 OR S7 | 4 |
| S8 | S1 OR S2 OR S3 OR S6 OR S7 | 27 |
| S7 | TI ( ((motion* N4 senso*) and (hyperactivity or ADHD)) ) OR AB ( ((motion* N4 senso*) and (hyperactivity or ADHD)) ) | 1 |
| S6 | S4 AND S5 | 1 |
| S5 | TI ( ("Attention Deficit Disorder" or ADHD) ) OR AB ( ("Attention Deficit Disorder" or ADHD) ) | 15,898 |
| S4 | TI ( (ARVO* or EFSim* or "EF Sim*" or EPELI or "Peili Vision Company") ) OR AB ( (ARVO* or EFSim* or "EF Sim*" or EPELI or "Peili Vision Company") ) | 169 |
| S3 | TI ( (Nesplora* or "Giunti psychometrics") ) OR AB ( (Nesplora* or "Giunti psychometrics") ) | 2 |
| S2 | TI ( (QbCheck* or "Qb Check*" or "(Qb) Check*") ) OR AB ( (QbCheck* or "Qb Check*" or "(Qb) Check*") ) | 1 |
| S1 | TI ( (QbTest* or "Qb Test*" or "(Qb) Test*" or "Qb Mini*" or "QbMini*" or (("Quantified Behavior*" or "Quantified Behaviour*") N4 test*) or QbTech) ) OR AB ( (QbTest* or "Qb Test*" or "(Qb) Test*" or "Qb Mini*" or "QbMini*" or (("Quantified Behavior*" or "Quantified Behaviour*") N4 test*) or QbTech) ) | 22 |

Resource: CTG

Host: <https://clinicaltrials.gov/>

Location: London, UK

Date of Search: 10 Sep. 24

((QbTest OR "Qb Test" OR "(Qb) Test" OR (("Quantified Behavior*" or "Quantified Behaviour*") AND test) or QbTech) OR (QbCheck OR "Qb Check" OR "(Qb) Check") OR (Nesplora OR "Giunti psychometrics") OR (ARVO OR EFSim OR "Peili Vision Company"))

Resource: ICTRP

Host: <https://trialsearch.who.int/>

Location: London, UK

Date of Search: 10 Sep. 24

((QbTest OR "Qb Test" OR "(Qb) Test" OR (("Quantified Behavior*" or "Quantified Behaviour*") AND test) or QbTech) OR (QbCheck OR "Qb Check" OR "(Qb) Check") OR (Nesplora OR "Giunti psychometrics") OR (ARVO OR EFSim OR "Peili Vision Company"))

# Appendix 3: ACTIVE framework of involvement in a systematic review

| **Framework constructs** | **Categories** | **Comments** |
| --- | --- | --- |
| Who was involved? | Patients, carers and/or their families |  |
|  | **Patients, carers and/or their families and other stakeholders** | Three patient representatives (two have ADHD, and one is the parent of a child with ADHD) and two clinicians were involved in this review |
|  | Other stakeholders only |  |
| How were stakeholders recruited? | Open – Fixed |  |
|  | Open – Flexible |  |
|  | **Closed – Invitation** | The three patient representatives and two clinicians were invited to participate through the use of known contacts |
|  | Closed – Existing group |  |
|  | Closed – Purposive sampling |  |
| What was the mode of involvement? (Approach) | One time |  |
|  | **Continuous** | Involvement of interest holders followed the “multiple-time closed event approach” in which they contributed to two 1.5hr online team meetings, one in the data extraction stage and one in the analysis phase, to discuss the results and how they should be interpreted and presented. One of the patient representatives supervised the qualitative synthesis, as she is a qualitative researcher. All five interest holders were invited to review this paper as co-authors – three accepted this invitation and are included as co-authors. The researchers asked the clinicians questions throughout the project. |
|  | Combined (both one-time and continuous) |  |
| What was the mode of involvement? (Methods) | **Direct interaction** | Direct interaction via Microsoft Teams meetings and email. |
|  | No direct interaction |  |
| At what stage in the review process did involvement occur? | **Develop question** | Patients and clinicians were involved by NICE in the development of the question for the original broader review conducted for NICE, therefore we know this is a topic of importance to patients. |
|  | Plan methods |  |
|  | Write and publish protocol |  |
|  | Develop search |  |
|  | Run search |  |
|  | Select studies |  |
|  | Collect data |  |
|  | Assess risk of bias |  |
|  | Analyse data |  |
|  | **Interpret findings** | Patient representatives and clinicians supported with the interpretation of findings and three reviewed the manuscript and provided feedback. |
|  | **Write and publish review** |  |
|  | Knowledge translation and impact |  |
| What was the level of involvement at each stage? | Leading |  |
|  | Controlling |  |
|  | **Influencing** | Patient representatives and clinicians provided information that directly and indirectly influenced the review process (e.g. provided their views on the importance of the QbTest for patients and clinicians, and how the results should be presented). All five interest holders were invited to review this paper as co-authors – three accepted this invitation and are included as co-authors. |
|  | **Contributing** |  |
|  | Receiving |  |

From: Pollock A, Campbell P, Struthers C, Synnot A, Nunn J, Hill S, Goodare H, Morris J, Watts C, Morley R. Development of the ACTIVE framework to describe stakeholder involvement in systematic reviews. Journal of health services research & policy. 2019 Oct;24(4):245-55.

# Appendix 4: Included, ongoing, and excluded studies

## Included studies

Table 1 Included studies

| **Study name** | **Primary Report** | **Secondary reports** | **Identified from** |
| --- | --- | --- | --- |
| FACT | Chitsabesan PH, C. L. Carter, L. A. Reeves, M. Mohammed, V. Beresford, B. Young, S. Kraam, A. Trowse, S. Wilkinson-Cunningham, L. Lennox, C. Using an objective computer task (QbTest) to aid the identification of attention deficit hyperactivity disorder (ADHD) in the Children and Young People Secure Estate (CYPSE): a feasibility randomised controlled trial. BMJ Open. 2022;12(12):e064951. | ISRCTN17402196. 2019. Feasibility trial to assess Attention Deficit Hyperactivity Disorder (ADHD) in the Criminal Justice System by using QbTest (a computer task). http://isrctn.com/ISRCTN17402196 (Accessed November 2023).  Lennox CH, C. L. Carter, L. A. Beresford, B. Young, S. Kraam, A. Brown, N. Wilkinson-Cunningham, L. Reeves, M. Chitsabesan, P. FACT: a randomised controlled trial to assess the feasibility of QbTest in the assessment process of attention deficit hyperactivity disorder (ADHD) for young people in prison -a feasibility trial protocol. BMJ Open. 2020;10(1):035519. | Main searches |
| NR | Hall Charlotte L, Selby Kim, Guo Boliang, Valentine Althea Z, Walker Gemma M, Hollis Chris,. Innovations in Practice: an objective measure of attention, impulsivity and activity reduces time to confirm attention deficit/hyperactivity disorder diagnosis in children - a completed audit cycle. Child and adolescent mental health. 2016;21(3):175-8. | None | Main searches |
| AQUA | Hollis CH, Hall Charlotte L., Guo Boliang, James Marilyn, Boadu Janet, Groom Madeleine J., Brown Nikki, Kaylor-Hughes Catherine, Moldavsky Maria, Valentine Althea Z., Walker Gemma M,. Daley David, Sayal Kapil, Morriss Richard. The impact of a computerised test of attention and activity (QbTest) on diagnostic decision-making in children and young people with suspected attention deficit hyperactivity disorder: single-blind randomised controlled trial. Journal of child psychology and psychiatry, and allied disciplines. 2018;59(12):1298-308. | Hall CLV, Althea Z. Walker, Gemma M. Ball, Harriet M. Cogger, Heather Daley, David Groom Madeleine J,. Sayal Kapil, Hollis Chris. Study of user experience of an objective test (QbTest) to aid ADHD assessment and medication management: a multi-methods approach. BMC psychiatry. 2017;17(1):66.  Hall CLW, Walker Gemma M,. Valentine Althea Z., Guo Boliang, Kaylor-Hughes Catherine, James Marilyn, Daley David, Sayal Kapil, Hollis Chris. Protocol investigating the clinical utility of an objective measure of activity and attention (QbTest) on diagnostic and treatment decision-making in children and young people with ADHD-'Assessing QbTest Utility in ADHD' (AQUA): a randomised controlled trial. BMJ open. 2014;4(12):e006838.  ISRCTN11727351. 2016. Comparing the effects of providing clinicians and patients with the results of an objective measure of activity and attention (QbTest) versus usual care on diagnostic and treatment decision making in children and young people with ADHD. https://www.isrctn.com/ISRCTN11727351 (Accessed November 2023).  NCT02209116. 2014. Assessing QbTest Utility in ADHD: A Randomised Controlled Trial. https://clinicaltrials.gov/show/NCT02209116 (Accessed November 2023). | Main searches |
| NR | Catriona Humphreys, Lucy Sitton-Kent. Transforming ADHD Care Across the East Midlands: An evaluation Report. East Midlands Academic Health Network. 2018. URL: https://healthinnovation-em.org.uk/component/rsfiles/download-file/files?path=our-work%252Four-innovations%252FTransforming-ADHD-Care%252FFinal_Overall_Evaluation_Report_31May18.pdf&Itemid=1457 (Accessed March 2024). | None | QbTest Manufacturer Submission |
| Focus ADHD | Hall SS, McKenzie C, Thomson L, Ingall BR, Groom MJ, McGlennon N, Dines-Allen M, Hall CL. A national evaluation of QbTest to support ADHD assessment: a real-world, mixed methods approach. BMC Health Services Research. 2024 Oct 8;24(1):1201. | Caitlin McKenzie, Benjamin-Rose Ingall, [Dr] Charlotte Hall. Focus ADHD National Programme Evaluation. 2022. URL: https://healthinnovation-em.org.uk/component/rsfiles/download-file/files?path=our-work%252Four-innovations%252FADHD%2BFOCUS%2Bevaluation%2Breport%2B-%2BFINAL%2Bv.1.0%2B18.10.22.pdf&Itemid=1457 (Accessed March 2024). | Peer review (updated report for trial published after main search and identified in peer review- no new data).  Secondary report: QbTest Manufacturer Submission |
| NR | Pellegrini SM, Mike Lovett, Ella. The QbTest for ADHD assessment: Impact and implementation in Child and Adolescent Mental Health Services. Children & Youth Services Review 2020;114.n.r. | None | Main searches |
| NR | Sharma RW, A. Lacey, S. Spiewakowski, D. IMPLEMENTING QB TESTING FOR ADHD: EVALUATING VALUE IN A DGH SETTING. Archives of Disease in Childhood. 2022;107(Supplement 2):A70. | None | Main searches |
| NR | Ulberstad FB, Hans Chavanon, Mira-Lynn Knollmann, Martin Wiley, James Christiansen, Hanna Thorell, Lisa B. Objective measurement of attention deficit hyperactivity disorder symptoms outside the clinic using the QbCheck: Reliability and validity. International journal of methods in psychiatric research. 2020;29(2):e1822. | None | Main searches |
| NR | Vogt CS, A. Assessments for attention-deficit hyperactivity disorder: Use of objective measurements. Psychiatrist. 2011;35(10):380-3. | None | Main searches |
| QUOTA | Williams LH, Charlotte L. Brown, Susan Guo, Boliang James, Marilyn Franceschini, Matilde Clarke, Julie Selby, Kim Vijayan, Hena Kulkarni, Neeta Brown, Nikki Sayal, Kapil Hollis, Chris Groom, Madeleine J. Optimising medication management in children and young people with ADHD using a computerised test (QbTest): a feasibility randomised controlled trial. Pilot and feasibility studies. 2021;7(1):68. | Hall CLB, Susan James, Marilyn Martin, Jennifer L. Brown, Nikki Selby, Kim Clarke, Julie Williams, Laura Sayal, Kapil Hollis, Chris Groom, Madeleine J. Consensus workshops on the development of an ADHD medication management protocol using QbTest: developing a clinical trial protocol with multidisciplinary stakeholders. BMC medical research methodology. 2019;19(1):126.  Hall CLJ, Marilyn Brown, Sue Martin, Jennifer L. Brown, Nikki Selby, Kim Clarke, Julie Vijayan, Hena Guo, Boliang Sayal, Kapil Hollis, Chris Groom, Madeleine J. Protocol investigating the clinical utility of an objective measure of attention, impulsivity and activity (QbTest) for optimising medication management in children and young people with ADHD 'QbTest Utility for Optimising Treatment in ADHD' (QUOTA): a feasibility randomised controlled trial. BMJ open. 2018;8(2):e021104.  ISRCTN69461593. 2018. QbTest Utility for Optimising Treatment in ADHD (QUOTA). https://www.isrctn.com/ISRCTN69461593 (Accessed November 2023).  NCT03368573. 2017. QbTest Utility for Optimising Treatment in ADHD (QUOTA). https://clinicaltrials.gov/show/NCT03368573 (Accessed November 2023). | Main searches |

## Ongoing studies

| **Report** | **Details** |
| --- | --- |
| Qbtech ongoing study trial registry: <https://onderzoekmetmensen.nl/en/trial/53743> | Validity study for the QbTest on smartphone (QbMT) in development, unclear if study will report relevant data |

## Excluded studies

Table 2 Studies excluded at full-text screening from the identification of studies via databases and registers

| **Report** | **Reason for exclusion** |
| --- | --- |
| 2014-001488-11. Effects of expectations, medication and placebo during the Quantified Behavior Test in patients with untreated ADHD and Substance Use Disorder. 2014. URL: https://www.clinicaltrialsregister.eu/ctr-search/search?query=eudract_number:2014-001488-11 (Accessed October 2023) | Not an evaluation of the test |
| Areces DD, Julie Garcia, Trinidad Gonzalez-Castro, Paloma Rodriguez, Celestino. Analysis of cognitive and attentional profiles in children with and without ADHD using an innovative virtual reality tool. PLOS ONE 2018;13(8): e0201039 | Not an evaluation of the test |
| Areces DG, Trinidad Cueli, Marisol Rodriguez, Celestino. Is a Virtual Reality Test Able to Predict Current and Retrospective ADHD Symptoms in Adulthood and Adolescence? Brain sciences 2019;9(10): .n.r. | Does not report on one of the outcomes of interest |
| Areces DR, Celestino Garcia, Trinidad Cueli, Marisol Gonzalez-Castro, Paloma. Efficacy of a Continuous Performance Test Based on Virtual Reality in the Diagnosis of ADHD and Its Clinical Presentations. Journal of attention disorders 2018;22(11): 1081-1091 | Does not report on one of the outcomes of interest |
| Baader AK, B. Brunkhorst-Kanaan, N. Kittel-Schneider, S. Reif, A. Grimm, O. A within-sample comparison of two innovative neuropsychological tests for assessing adhd. Brain Sciences 2021;11(1): 1-21 | Does not report on one of the outcomes of interest |
| Baader AK, B. Brunkhorst-Kanaan, N. Kittel-Schneider, S. Reif, A. Grimm, O. A within-sample comparison of two innovative neuropsychological tests for assessing adhd. Brain Sciences 2021;11(1): 1-21 | Duplicate report |
| Baader AK, B. Brunkhorst-Kanaan, N. Kittel-Schneider, S. Reif, A. Grimm, O. P.632 A within-sample comparison of two innovative neuropsychological tests for diagnosing ADHD. European neuropsychopharmacology 2020;40(Supplement 1): S355-S356 | Does not report on one of the outcomes of interest |
| Bellato AH, Charlotte L. Groom, Madeleine J. Simonoff, Emily Thapar, Anita Hollis, Chris Cortese, Samuele. Practitioner Review: Clinical utility of the QbTest for the assessment and diagnosis of attention-deficit/hyperactivity disorder - a systematic review and meta-analysis. *Journal of child psychology and psychiatry, and allied disciplines* 2023;.n.r. | SR |
| Berger I, Slobodin O, Cassuto H. Usefulness and validity of continuous performance tests in the diagnosis of attention-deficit hyperactivity disorder children. Archives of Clinical Neuropsychology 2017;32(1): 81-93 | Did not report on test of interest |
| Bhattacharyya NS, S. Banerjee, A. Ghosh, R. Sinha, O. Das, N. Gayen, R. Pal, S. S. Ganguly, S. Dasgupta, T. Mondal, P. Adhikari, A. Sarkar, S. Bhattacharyya, D. Mallick, A. K. Singh, O. P. Pal, S. K. Integration of electroencephalogram (EEG) and motion tracking sensors for objective measure of attention-deficit hyperactivity disorder (MAHD) in pre-schoolers. The Review of scientific instruments 2022;93(5): 054101 | Did not report on test of interest |
| Bijlenga DJ, M. Gehlhaar, S. K. Sandra Kooij, J. J. Objective QbTest and subjective evaluation of stimulant treatment in adult attention deficit-hyperactivity disorder. European psychiatry : the journal of the Association of European Psychiatrists 2015;30(1): 179-185 | Does not report on one of the outcomes of interest |
| Brancaccio RK, J. Ayearst, L. E. Using wearables and artificial intelligence to improve diagnostic decisions and treatment in youth with attention-deficit hyperactivity disorder. Innovations in Clinical Neuroscience 2021;18(10-12 SUPPL): S2-S3 | Did not report on test of interest |
| Brocki KCT, Carin M. Bohlin, Gunilla. CPT performance, motor activity, and continuous relations to ADHD symptom domains: A developmental study. European Journal of Developmental Psychology 2010;7(2): 178-197 | Not an evaluation of the test |
| Camacho-Conde JAC, Gema. Attentional profile of adolescents with ADHD in virtual-reality dual execution tasks: A pilot study. Applied Neuropsychology: Child 2022;11(1): 81-90 | Not an evaluation of the test |
| Cedergren K, Östlund S, Åsberg Johnels J, Billstedt E, Johnson M. Monitoring medication response in ADHD: What can continuous performance tests tell us? European Archives of Psychiatry and Clinical Neuroscience 2022;272(2): 291-299 | Duplicate report |
| Cedergren K, Östlund S, Åsberg Johnels J, Billstedt E, Johnson M. Monitoring medication response in ADHD: What can continuous performance tests tell us? European Archives of Psychiatry and Clinical Neuroscience 2022;272(2): 291-299 | Not an evaluation of the test |
| Climent GR, Celestino Garcia, Trinidad Areces, Debora Mejias, Miguel Aierbe, Amaia Moreno, Marta Cueto, Eduardo Castella, Judit Feli Gonzalez, Mari. New virtual reality tool (Nesplora Aquarium) for assessing attention and working memory in adults: A normative study. Applied neuropsychology Adult 2021;28(4): 403-415 | Does not report on one of the outcomes of interest |
| Climent GR, Celestino Garcia, Trinidad Areces, Debora Mejias, Miguel Aierbe, Amaia Moreno, Marta Cueto, Eduardo Castella, Judit Feli Gonzalez, Mari. New virtual reality tool (Nesplora Aquarium) for assessing attention and working memory in adults: A normative study. Applied neuropsychology Adult 2021;28(4): 403-415 | Duplicate report |
| Cole E. Qb test improves diagnosis of attention deficit disorder. Nursing children and young people 2015;27(2): 10-11 | Not a primary study or SR |
| Diaz-Orueta U. Advances in neuropsychological assessment of attention: From initial computerized continuous performance tests to AULA. The role of technology in clinical neuropsychology 2017;103.n.r. | Not a primary study or SR |
| Diaz-Orueta UF-F, M. A. Morillo-Rojas, M. D. Climent, G. [Efficacy of lisdexamphetamine to improve the behavioural and cognitive symptoms of attention deficit hyperactivity disorder: treatment monitored by means of the AULA Nesplora virtual reality test]. Eficacia de la lisdexanfetamina en la mejora sintomatica conductual y cognitiva del trastorno por deficit de atencion/ hiperactividad: tratamiento monitorizado mediante el test AULA Nesplora de realidad virtual 2016;63(1): 19-27 | Not an evaluation of the test |
| Diaz-Orueta UG-L, Cristina Crespo-Eguilaz, Nerea Sanchez-Carpintero, Rocio Climent, Gema Narbona, Juan. AULA virtual reality test as an attention measure: convergent validity with Conners' Continuous Performance Test. Child neuropsychology : a journal on normal and abnormal development in childhood and adolescence 2014;20(3): 328-342 | Does not report on one of the outcomes of interest |
| [DRKS00030766. Identification of objective markers for the evaluation and prediction of the treatment of children and adolescents with ADHD. 2022. URL: http://drks.de/search/en/trial/DRKS00030766 (Accessed October 2024).](http://drks.de/search/en/trial/DRKS00030766) | Not an evaluation of the test |
| Faraone SV, Banaschewski T, Coghill D, Zheng Y, Biederman J, Bellgrove MA, et al. The World Federation of ADHD International Consensus Statement: 208 Evidence-based conclusions about the disorder. Neuroscience and Biobehavioral Reviews 2021;128789-818 | Background |
| Fernandez-Martin PL, J. J. Rodriguez-Herrera, R. Canovas, R. Martinez De Salazar, A. Cobos-Sanchez, L. Sanchez-Santed, F. Flores, P. Dimensional analysis of adolescent attention-deficit/ hyperactivity disorder. European Psychiatry 2020;63(Supplement 1): S677 | Not an evaluation of the test |
| Fernandez-Martin PR-H, Rocio Canovas, Rosa Diaz-Orueta, Unai Martinez de Salazar, Alma Flores, Pilar. Data-driven profiles of attention-deficit/hyperactivity disorder using objective and ecological measures of attention, distractibility, and hyperactivity. European child & adolescent psychiatry 2023[Epub ahead of print] | Does not report on one of the outcomes of interest |
| Fischer SK, M. Lehfeld, H. Niklewski, G. Brandl, C. Influence of depressive symptoms on Qb test performance in adult ADHD patients. ADHD Attention Deficit and Hyperactivity Disorders 2015;7(SUPPL. 1): S77 | Does not report on one of the outcomes of interest |
| Garcia Murillo LC, S. Anderson, D. Di Martino, A. Castellanos, F. Meta-analysis of locomotor activity measures in attention-deficit/hyperactivity disorder. European Child and Adolescent Psychiatry 2015;24(1 SUPPL. 1): S154 | Did not report on test of interest |
| Hager LAO, Geir Danielsen, Maria Billstedt, Eva Gillberg, Christopher Johnels, Jakob Asberg. Indexing executive functions with test scores, parent ratings and ERPs: How do the measures relate in children versus adolescents with ADHD? [References].DP - Feb 17, 2020. Neuropsychiatric Disease and Treatment 2020;16465-477 | Not an evaluation of the test |
| Hall CLB, A. Kirk, J. D. Hollis, C. The clinical utility of QbTest in supporting the assessment and monitoring of attention-deficit/hyperactivity disorder (ADHD): what do paediatricians need to know? Paediatrics and Child Health (United Kingdom) 2023;33(9): 259-264 | Background |
| Hall CLV, Althea Z. Groom, Madeleine J. Walker, Gemma M. Sayal, Kapil Daley, David Hollis, Chris. The clinical utility of the continuous performance test and objective measures of activity for diagnosing and monitoring ADHD in children: A systematic review. European child & adolescent psychiatry 2016;25(7): 677-699 | SR |
| Hall CLW, G. M. Valentine, A. Z. Correction. Protocol investigating the clinical utility of an objective measure of activity and attention (QbTest) on diagnostic and treatment decision-making in children and young people with ADHD - 'Assessing QbTest Utility in ADHD' (AQUA): a randomised controlled trial. BMJ open 2015;5(5): e006838corr006831 | Erratum |
| Hall CLW, G. M. Valentine, A. Z. Erratum: Protocol investigating the clinical utility of an objective measure of activity and attention (QbTest) on diagnostic and treatment decision-making in children and young people with ADHD - 'Assessing QbTest Utility in ADHD' (AQUA): A randomised controlled trial (BMJ Open (2014) 4 (e006838)). BMJ Open 2015;5(5): 006838corr006831 | Erratum |
| Hall CLW, G. M. Valentine, A. Z. Erratum: Protocol investigating the clinical utility of an objective measure of activity and attention (QbTest) on diagnostic and treatment decision-making in children and young people with ADHD-'Assessing QbTest Utility in ADHD'(AQUA): A randomised controlled trial (BMJ Open (2014) 4 (e006838)). BMJ Open 2016;6(1): e006838 | Erratum |
| Hamadache SH, Kathrin Labarga, Sara Zaplana Gunther, Thomas. Is the QbMini a valid instrument for ADHD assessment? [References].DP - Aug 2021. Journal of attention disorders 2021;25(10): 1384-1394 | Duplicate report |
| Hirsch OC, Hanna. Factorial Structure and Validity of the Quantified Behavior Test Plus (Qb+©). Assessment 2017;24(8): 1037-1049 | Does not report on one of the outcomes of interest |
| Iriarte YD-O, Unai Cueto, Eduardo Irazustabarrena, Paula Banterla, Flavio Climent, Gema. AULA-Advanced virtual reality tool for the assessment of attention: Normative study in Spain. Journal of attention disorders 2016;20(6): 542-568 | Does not report on one of the outcomes of interest |
| Jansson LL, Monica Ostlund, Mona Domingo, Blanca. Effects of one single-dose methylphenidate compared to one single-dose placebo on QbTest performance in adults with untreated ADHD: a randomized controlled trial. BMC Psychiatry 2023;23(1): 762 | Not an evaluation of the test |
| Jylkka JR, Liisa Merzon, Liya Kangas, Suvi Kliegel, Matthias Zuber, Sascha Hering, Alexandra Laine, Matti Salmi, Juha. Assessment of goal-directed behavior and prospective memory in adult ADHD with an online 3D videogame simulating everyday tasks. Scientific reports 2023;13(1): 9299 | Did not report on test of interest |
| Knez RS, Dejan Nasic, Salmir Doric, Ana Wentz, Elisabet. The Impact of Methylphenidate on QbTest Performance of Children with ADHD: A Retrospective Clinical Study. Neuropsychiatric disease and treatment 2021;1719-32 | Not an evaluation of the test |
| Kooij JJS, Bijlenga D, Salerno L, Jaeschke R, Bitter I, Balázs J, et al. Updated European Consensus Statement on diagnosis and treatment of adult ADHD. European Psychiatry 2019;5614-34 | Background |
| Kuhle H. J., Lefering R. Video-assisted behavior observation as a tool for methylphenidate dose finding in ADHD: Longer term outcome. Neuropediatrics 2013;44(2): PS20-1146 | Did not report on test of interest |
| Kvitland LRJ, K. Achkhan, H. Berg, T. Dahlen, N. R. Kirkholt, G. M. Koren, K. N. Naess, M. F. The CPT-3 versus the QB-test: A task-oriented computerized assessment of attention-related problems in out-patient children: Will diagnosis predict the atypical attention scores? ADHD Attention Deficit and Hyperactivity Disorders 2019;11(1 Supplement): S18-S19 | Does not report on one of the outcomes of interest |
| Lindhiem OG, Mayank Shaaban, Sam Mak, Kristie J. Chikersal, Prerna Feldman, Jamie Harris, Jordan L. Objective Measurement of Hyperactivity Using Mobile Sensing and Machine Learning: Pilot Study. JMIR formative research 2022;6(4): e35803 | Did not report on test of interest |
| Lohman MD, Blanca Ostlund, Mona Jansson, Lennart. Contrasting expectancy effects with objective measures in adults with untreated ADHD during QbTest. Scandinavian journal of psychology 2023;64(4): 461-469 | Not an evaluation of the test |
| Luderer MS, Johanna Gerhardt, Sarah Hoffmann, Sabine Vollstadt-Klein, Sabine Reif, Andreas Sobanski, Esther. Drinking alcohol to cope with hyperactive ADHD? Self-reports vs. continuous performance test in patients with ADHD and/or alcohol use disorder. Frontiers in psychiatry 2023;141112843 | Does not report on one of the outcomes of interest |
| Manning D, Olety S. Qb technology - evaluating its use in adhd diagnosis within a child and adolescent mental health service. European Psychiatry 2021;64(Supplement 1): S225 | Does not report on one of the outcomes of interest |
| Marshall P, Hoelzle J, Nikolas M. Diagnosing attention-deficit/hyperactivity disorder (ADHD) in young adults: A qualitative review of the utility of assessment measures and recommendations for improving the diagnostic process. The Clinical Neuropsychologist 2021;35(1): 165-198 | SR |
| Martin-Key NA, Stevenson A, Roy P. Investigating the Clinical Utility of the Combined Use of Objective and Subjective Measures of ADHD During Treatment Optimization. Journal of clinical psychopharmacology 2022;42(2): 146-153 | Does not report on one of the outcomes of interest |
| [NCT02473185. Effects of Expectation, Medication and Placebo on Objective and Self-rated Performance During the QbTest. 2015. URL: https://clinicaltrials.gov/show/NCT02473185 (Accessed October 2023).](https://clinicaltrials.gov/show/NCT02473185) | Not an evaluation of the test |
| [NCT02477280. Effects of Expectation, Medication and Placebo on Objective and Self-rated Performance. 2015. URL: https://clinicaltrials.gov/show/NCT02477280 (Accessed October 2023).](https://clinicaltrials.gov/show/NCT02477280) | Not an evaluation of the test |
| Nylander Elin, Sparding Timea, Floros Orestis, Ryden Eleonore, Landen Mikael, Hansen Stefan. The quantified behavioural test plus (qbtest+) in adult adhd. *Nordic Psychology* 2022;75(1):20-34. | Does not report on one of the outcomes of interest |
| Peñuelas-Calvo I, Jiang-Lin LK, Girela-Serrano B, Delgado-Gomez D, Navarro-Jimenez R, Baca-Garcia E, et al. Video games for the assessment and treatment of attention-deficit/hyperactivity disorder: a systematic review. European Child and Adolescent Psychiatry 2022;31(1): 5-20 | SR |
| Prasad V, Rezel-Potts E, White P, Downs J, Boddy N, Sayal K, et al. Use of healthcare services before diagnosis of attention-deficit/hyperactivity disorder: a population-based matched case-control study. Archives of disease in childhood 2023;109(1): 46-51 | Background |
| Puzzo IS, Ottilie Kelly, Rachel Greer, Ben Kumari, Veena Gujonsson, Gisli Young, Susan. Attention problems predict risk of violence and rehabilitative engagement in mentally disordered offenders. Frontiers in Psychiatry 2019;10279 | Not an evaluation of the test |
| Ramtvedt B E, Sundet K. Relationships between computer-based testing and behavioral ratings in the assessment of attention and activity in a pediatric ADHD stimulant crossover trial. The Clinical Neuropsychologist 2014;28(7): 1146-1161 | Does not report on one of the outcomes of interest |
| Reh VS, Martin Lam, Le Schimmelmann, Benno G. Hebebrand, Johannes Rief, Winfried Christiansen, Hanna. Behavioral Assessment of Core ADHD Symptoms Using the QbTest. Journal of attention disorders 2015;19(12): 1034-1045 | Does not report on one of the outcomes of interest |
| Rodriguez CA, Debora Garcia, Trinidad Cueli, Marisol Gonzalez-Castro, Paloma. Comparison between two continuous performance tests for identifying ADHD: Traditional vs. virtual reality. International journal of clinical and health psychology 2018;18(3): 254-263 | Does not report on one of the outcomes of interest |
| Santosh P, Cortese S, Hollis C, Bölte S, Daley D, Coghill D, et al. Remote assessment of adhd in children and adolescents: Recommendations from the european adhd guidelines group following the clinical experience during the covid-19 pandemic. European child & adolescent psychiatry 2023;32(6): 921-935 | Background |
| Sanwo O, Huzair H. What's new in attention-deficit/hyperactivity disorder: updates on assessment and management. Paediatrics and Child Health (United Kingdom) 2022;32(8): 282-289 | Background |
| Schworer M, Jascenoka J, Nitkowski D, Petermann F, Vasileva M, Petermann U. Deficits in executive functions of children with ADHD: Clinical validity of a diagnostic instrument for ADHD in children and adolescents (ADHS-KJ). Kindheit und Entwicklung: Zeitschrift fur Klinische Kinderpsychologie 2019;28(2): 96-105 | Did not report on test of interest |
| Selaskowski BA, Laura Marie Wiebe, Annika Kannen, Kyra Aslan, Behrem Gerding, Thiago Morano Sanchez, Dario Ettinger, Ulrich Kolle, Markus Lux, Silke Philipsen, Alexandra Braun, Niclas. Gaze-based attention refocusing training in virtual reality for adult attention-deficit/hyperactivity disorder. BMC Psychiatry 2023;2374 | Did not report on test of interest |
| Slobodin O, Davidovitch M. Gender differences in objective and subjective measures of ADHD among clinic-referred children. Frontiers in Human Neuroscience 2019;13441 | Did not report on test of interest |
| Stevanovic DW, Elisabet Nasic, Salmir Knez, Rajna. ASD with ADHD vs. ASD and ADHD alone: a study of the QbTest performance and single-dose methylphenidate responding in children and adolescents. BMC Psychiatry 2022;22(1): 282 | Does not report on one of the outcomes of interest |
| Stuart E, Torres S, Gutierrez B. B - 04 Evaluating the Efficacy of a Virtual Reality Neuropsychological Assessment in Detecting ADHD Subtypes. Archives of clinical neuropsychology : the official journal of the National Academy of Neuropsychologists 2023;38(7): 1368 | Does not report on one of the outcomes of interest |
| Valentine AZ, Brown BJ, Groom MJ, Young E, Hollis C, Hall CL. A systematic review evaluating the implementation of technologies to assess, monitor and treat neurodevelopmental disorders: A map of the current evidence. Clinical Psychology Review 2020;80101870 | SR |
| Vogt C. Clinical Conundrums When Integrating the QbTest into a Standard ADHD Assessment of Children and Young People. Neuropediatrics 2021;52(3): 155-162 | Background |
| Wang XQ, Albitos PJ, Hao YF, Zhang H, Yuan LX, Zang YF. A review of objective assessments for hyperactivity in attention deficit hyperactivity disorder. Journal of neuroscience methods 2022;370109479 | Not a primary study or SR |
| Wehmeier P, Bender M. ADHD core symptom assessment in adults with ADHD, depression, addiction or borderline personality disorder using the Qb test. ADHD Attention Deficit and Hyperactivity Disorders 2017;9(1 Supplement): S13 | Does not report on one of the outcomes of interest |
| Wehmeier P, Wolff J, Cabanas N, Bender M. ADHD core symptom assessment in adults with ADHD compared to adults with ADHD and comorbid borderline personality disorder using a computer-based continuous performance test (cb-CPT) combined with an infra-red motion-tracking device. ADHD Attention Deficit and Hyperactivity Disorders 2019;11(1 Supplement): S22 | Does not report on one of the outcomes of interest |
| Wehmeier PM, Dittmann RW, Banaschewski T, Schacht A. Does stimulant pretreatment modify atomoxetine effects on core symptoms of ADHD in children assessed by quantitative measurement technology? Journal of attention disorders 2014;18(2): 105-116 | Not an evaluation of the test |
| Wehmeier PM, Schacht A, Ulberstad F, Lehmann M, Schneider-Fresenius C, Lehmkuhl G, et al. Does atomoxetine improve executive function, inhibitory control, and hyperactivity? Results from a placebo-controlled trial using quantitative measurement technology. Journal of Clinical Psychopharmacology 2012;32(5): 653-660 | Not an evaluation of the test |
| Wehmeier PMK, Laura Banaschewski, Tobias Dittmann, Ralf W. Schacht, Alexander. Does comorbid disruptive behavior modify the effects of atomoxetine on ADHD symptoms as measured by a continuous performance test and a motion tracking device? [References].DP - Jul 2015. Journal of attention disorders 2015;19(7): 591-602 | Not an evaluation of the test |
| Wehrmann T, Jorg M. An objective measure of hyperactivity aspects with compressed webcam video. Child and adolescent psychiatry and mental health 2015;945 | Did not report on test of interest |
| Williams LH, Charlotte L. Brown, Susan Guo, Boliang James, Marilyn Franceschini, Matilde Clarke, Julie Selby, Kim Vijayan, Hena Kulkarni, Neeta Brown, Nikki Sayal, Kapil Hollis, Chris Groom, Madeleine J. Correction to: Optimising medication management in children and young people with ADHD using a computerised test (QbTest): a feasibility randomised controlled trial. Pilot and feasibility studies 2021;7(1): 94 | Erratum |
| Young SA, Nicoletta Asgeirsdottir, Bryndis Bjork Branney, Polly Beckett, Michelle Colley, William Cubbin, Sally Deeley, Quinton Farrag, Emad Gudjonsson, Gisli Hill, Peter Hollingdale, Jack Kilic, Ozge Lloyd, Tony Mason, Peter Paliokosta, Eleni Perecherla, Sri Sedgwick, Jane Skirrow, Caroline Tierney, Kevin van Rensburg, Kobus Woodhouse, Emma. Females with ADHD: An expert consensus statement taking a lifespan approach providing guidance for the identification and treatment of attention-deficit/ hyperactivity disorder in girls and women. BMC Psychiatry 2020;20404 | Background |
| Young SA, Philip Lloyd, Tony Absoud, Michael Arif, Muhammad Colley, William Andrew Cortese, Samuele Cubbin, Sally Doyle, Nancy Morua, Susan Dunn Ferreira-Lay, Philip Gudjonsson, Gisli Ivens, Valerie Jarvis, Christine Lewis, Alexandra Mason, Peter Newlove-Delgado, Tamsin Pitts, Mark Read, Helen van Rensburg, Kobus Zoritch, Bozhena Skirrow, Caroline. Failure of Healthcare Provision for Attention-Deficit/Hyperactivity Disorder in the United Kingdom: A Consensus Statement. Frontiers in psychiatry 2021;12649399 | Background |
| Hamadache SH, Kathrin Labarga, Sara Zaplana Gunther, Thomas. Is the QbMini a valid instrument for ADHD assessment? [References].DP - Aug 2021. Journal of Attention Disorders 2021;25(10): 1384-94 | Does not report on one of the outcomes of interest |
| Labarga SZH, Kathrin Hamadache, Salsabil Gunther, Thomas. Validation of the QbMini Test to diagnose Attention Deficit and Hyperactivity Disorder (ADHD) in 5-year-old children. Zeitschrift fur Neuropsychologie 2019;30(3): 149-56 | Does not report on one of the outcomes of interest |
| Gunther TL, S. V. N. Z. Hoberg, K. First validation of the QbMini to measure symptoms of ADHD in 5-year old children. ADHD Attention Deficit and Hyperactivity Disorders 2017;9(1 Supplement): S15 | Does not report on one of the outcomes of interest |
| Hult NK, Josefin Kadesjo, Bjorn Gillberg, Christopher Billstedt, Eva. ADHD and the QbTest: Diagnostic Validity of QbTest. Journal of attention disorders. 2018;22(11):1074-80. | Does not report on one of the outcomes of interest |
| Adamou MJ, Sarah L. Marks, Laura Lowe, Deborah. Efficacy of Continuous Performance Testing in Adult ADHD in a Clinical Sample Using QbTest. Journal of attention disorders. 2022;26(11):1483-91. | Does not report on one of the outcomes of interest |
| Brunkhorst-Kanaan NV, Moritz Kittel-Schneider, Sarah Vainieri, Isabella Reif, Andreas Grimm, Oliver. The Quantified Behavioral Test-A Confirmatory Test in the Diagnostic Process of Adult ADHD? Frontiers in psychiatry. 2020;11:216. | Does not report on one of the outcomes of interest |
| Edebol HH, Lars Norlander, Torsten. Measuring adult Attention Deficit Hyperactivity Disorder using the Quantified Behavior Test Plus. Psychology journal 2013;2(1): 48-62 | Does not report on one of the outcomes of interest |
| Edebol HH, Lars Norlander, Torsten. Objective Measures of Behavior Manifestations in Adult ADHD and Differentiation from Participants with Bipolar II Disorder, Borderline Personality Disorder, Participants with Disconfirmed ADHD as Well as Normative Participants. Clinical Practice and Epidemiology in Mental Health 2012;8134-43 | Does not report on one of the outcomes of interest |
| Johansson VNS, Eva Kuja-Halkola, Ralf Lundstrom, Sebastian Durbeej, Natalie Anckarsater, Henrik Lichtenstein, Paul Hellner, Clara. The Quantified Behavioral Test Failed to Differentiate ADHD in Adolescents With Neurodevelopmental Problems. Journal of attention disorders. 2021;25(3):312-21. | Does not report on one of the outcomes of interest |
| Pettersson RS, Staffan Nilsson, Kent W. Diagnosing ADHD in adults: An examination of the discriminative validity of neuropsychological tests and diagnostic assessment instruments. Journal of Attention Disorders. 2018;22(11):1019-31. | Does not report on one of the outcomes of interest |
| Soderstrom SP, Richard Nilsson, Kent W. Quantitative and subjective behavioural aspects in the assessment of attention-deficit hyperactivity disorder (ADHD) in adults. Nordic journal of psychiatry. 2014;68(1):30-7. | Does not report on one of the outcomes of interest |
| Stevanovic DN, Salmir Doric, Ana Wentz, Elisabet Knez, Rajna. The Structure and Diagnostic Accuracy of the QbTest in Pediatric ADHD: A Retrospective Clinical Study. Journal of attention disorders. 2023;27(11):1296-305. | Does not report on one of the outcomes of interest |
| Tallberg PR, Maria Wenhov, Lena Eliasson, Glen Gustafsson, Peik. Incremental clinical utility of continuous performance tests in childhood ADHD - an evidence-based assessment approach. Scandinavian journal of psychology. 2019;60(1):26-35. | Does not report on one of the outcomes of interest |
| Gustafsson PT, P. Towards evidence-based assessments: Clinical utility of rating scales and cognitive test methods in diagnostic assessment and treatment evaluations in children and adolescents with Attention-Deficit/Hyperactivity Disorder. ADHD Attention Deficit and Hyperactivity Disorders. 2017;9(1 Supplement):S15. | Does not report on one of the outcomes of interest |
| Seesjarvi EP, Jasmin Aronen, Eeva T. Lipsanen, Jari Mannerkoski, Minna Hering, Alexandra Zuber, Sascha Kliegel, Matthias Laine, Matti Salmi, Juha. Quantifying ADHD Symptoms in Open-Ended Everyday Life Contexts With a New Virtual Reality Task. Journal of attention disorders. 2022;26(11):1394-411. | Did not report on test of interest |
| Zulueta AD-O, Unai Crespo-Eguilaz, Nerea Torrano, Fermin. Virtual reality-based assessment and rating scales in ADHD diagnosis. Psicologia Educativa. 2019;25(1):13-22. | Did not report on test of interest |
| Emser TSJ, Blair A. Steele, J. Douglas Kooij, Sandra Thorell, Lisa Christiansen, Hanna. Assessing ADHD symptoms in children and adults: Evaluating the role of objective measures. Behavioral and Brain Functions. 2018;14:11. | Does not report on one of the outcomes of interest |
| NCT05846815 (Sponsors: Peili Vision) | Did not report on test of interest |
| Bijlenga DU, Fredrik Thorell, Lisa B. Christiansen, Hanna Hirsch, Oliver Kooij, J. J. Sandra. Objective assessment of attention-deficit/hyperactivity disorder in older adults compared with controls using the QbTest. International journal of geriatric psychiatry. 2019;34(10):1526-33. | Does not report on one of the outcomes of interest |
| Groom MJY, Zoe Hall, Charlotte L. Gillott, Alinda Hollis, Chris. The incremental validity of a computerised assessment added to clinical rating scales to differentiate adult ADHD from autism spectrum disorder. Psychiatry Research. 2016;243:168-73. | Does not report on one of the outcomes of interest |
| Wettstein R, Navarro Ovando V, Pirgon E, Kroesen J, Wettstein K, Kroesen H, Mathôt R, Dumont G. Absent or Hidden? Hyperactivity in Females With ADHD. Journal of Attention Disorders. 2024 Aug 19:10870547241273152. | Not an evaluation of the test; Does not report on one of the outcomes of interest |
| Arrondo G, Mulraney M, Iturmendi-Sabater I, Musullulu H, Gambra L, Niculcea T, Banaschewski T, Simonoff E, Döpfner M, Hinshaw SP, Coghill D. Systematic review and Meta-analysis: clinical utility of continuous performance tests for the identification of attention-deficit/hyperactivity disorder. Journal of the American Academy of Child & Adolescent Psychiatry. 2024 Feb 1;63(2):154-71. | SR |
| Peterson BS, Trampush J, Brown M, Maglione M, Bolshakova M, Rozelle M, Miles J, Pakdaman S, Yagyu S, Motala A, Hempel S. Tools for the diagnosis of ADHD in children and adolescents: a systematic review. Pediatrics. 2024 Apr 1;153(4):e2024065854. | SR |

Table 3 Studies excluded at full text screening from checking manufacturer websites

| **Study details** | **Manufacturer’s website** | **Reason for exclusion** |
| --- | --- | --- |
| Lis S, Baer N, Stein‐en‐Nosse C, Gallhofer B, Sammer G, Kirsch P. Objective measurement of motor activity during cognitive performance in adults with attention‐deficit/hyperactivity disorder. Acta Psychiatrica Scandinavica. 2010 Oct;122(4):285-94. | QbTech | Does not report on one of the outcomes of interest |

Table 4 Studies excluded at full text screening from checking the studies included in systematic reviews

| **Study details** | **Reason for exclusion** |
| --- | --- |
| Delgado-Gomez D, Peñuelas-Calvo I, Masó-Besga AE, VallejoOñate S, Tello IB, Duarte EA et al (2017) Microsoft kinect-based continuous performance test: an objective attention defcit hyperactivity disorder assessment. J Med Internet Res 19(3):e79 | Did not report on test of interest |
| Faraone SV, Newcorn JH, Antshel KM, Adler L, Roots K, Heller M (2016) The groundskeeper gaming platform as a diagnostic tool for attention-defcit/hyperactivity disorder: sensitivity, specifcity, and relation to other measures. J Child Adolesc Psychopharmacol 26(8):672–685 |  |
| Heller MD, Roots K, Srivastava S, Schumann J, Srivastava J, Hale TS (2013) A machine learning-based analysis of game data for attention defcit hyperactivity disorder assessment. Games Health J 2(5):291–298 |  |
| Pollak Y, Weiss PL, Rizzo AA, Weizer M, Shriki L, Shalev RS et al (2009) The utility of a continuous performance test embedded in virtual reality in measuring ADHD-related defcits. J Dev Behav Pediatr 30(1):2–6 |  |
| Shaw R, Grayson A, Lewis V (2005) Inhibition, ADHD, and computer games: the inhibitory performance of children with ADHD on computerized tasks and games. J Atten Disord 8(4):160–168 |  |
| Wehmeier PM, Schacht A, Wolff C, Otto WR, Dittmann RW, Banaschewski T. Neuropsychological outcomes across the day in children with attention-deficit/hyperactivity disorder treated with atomoxetine: results from a placebo-controlled study using a computer-based continuous performance test combined with an infra-red motion-tracking device. Journal of child and adolescent psychopharmacology. 2011 Oct 1;21(5):433-44. | Not an evaluation of the test |
| Reh V, Schmidt M, Lam L, Schimmelmann BG, Hebebrand J, Rief W, Christiansen H (2013) Behavioral assessment of core ADHD symptoms using the QbTest. J Atten Disord. doi:10.1177/1087054712472981 | Does not report on one of the outcomes of interest |
| Edebol HH, Lars Holmberg, Ebba Gustafsson, Stig-Arne Norlander, Torsten. In search for objective measures of hyperactivity, impulsivity and inattention in adult attention deficit hyperactivity disorder using the Quantified Behavior Test Plus. Europe’s Journal of Psychology. 2011;7(3):443-57. | Does not report on one of the outcomes of interest |

Table 5 Studies excluded at full text screening from checking the QbTech Manufacturer Submission

| **Study Details** | **Reason for exclusion** |
| --- | --- |
| Ulberstadt et al, the 6th World Congress on ADHD, April 20 - April 23, 2017, Vancouver, Canada | Does not report on one of the outcomes of interest |
| Wehmeier PM, Schacht A, Wolff C, Otto WR, Dittmann RW, Banaschewski T. Neuropsychological outcomes across the day in children with attention-deficit/hyperactivity disorder treated with atomoxetine: results from a placebo-controlled study using a computer-based continuous performance test combined with an infra-red motion-tracking device. J Child Adolesc Psychopharmacol 2011;21:433–44. https://doi.org/10.1089/cap.2010.0142 | Not an evaluation of the test |
| Roughan LA, Stafford J. Demand and capacity in an ADHD team: reducing the wait times for an ADHD assessment to 12 weeks. BMJ Open Qual. 2019 Oct 30;8(4):e000653. doi: 10.1136/bmjoq-2019-000653. PMID: 31750403; PMCID: PMC6830462 | Did not report on test of interest |
| Gustafsson U, Hansen M. QbTest in the clinical assessment of attention deficit hyperactivity disorder: A review of the evidence. Mental Health Science. 2023. | Systematic review (we screened the studies) |
| Gustafsson U, Hansen M. QbTest for Monitoring Medication Treatment Response in ADHD: A Systematic Review. Clinical Practice & Epidemiology in Mental Health. 2023. | Systematic review (we screened the studies) |
| Confidential study | Confidential and provided by company |
| Confidential study | Confidential and provided by company |

# Appendix 5: Data extraction

## Baseline data

| **Author and year** | **Study component** | **Study Details** | **Participants** | **Interventions and confounders** |
| --- | --- | --- | --- | --- |
| Chitsabesan (2022)(1) | RCT | **Study Name**  FACT  **Country**  England  **Language**  English  **Setting**  Young Offenders Institution (YOI)  **Study design**  Single-centre feasibility RCT with embedded qualitative and survey components  **Funding**  Non-industry | **Population:** ADHD diagnosis in boys aged 15-18 years  **Inclusion Criteria:** Boys aged 15-18 years from a YOI who had any ADHD symptom from the Comprehensive Health Assessment Tool.  **Exclusion Criteria:** Being on remand; not speaking English; previous/ current ADHD diagnosis; risk to researcher/ staff; unable to give informed consent (16yr+) or no guardian consent (under 16yr).  **Number participants included (analysed):** 60 (47 at 3m, 19 at 6m)  **Age**  QbTest - age 16: 20%; 17: 26.7%; 18: 50%; missing: 3.3%.  Control - age 16: 10%; 17: 36.7%; 18: 53.3%; missing: 0%.  **Sex (% male)**  100 | **Group 1: QbTest and usual care (n=30 randomised; 20 completed test):**  QbTest completed prior to first assessment by neurodevelopmental lead. Information from QbTest, plus clinical information, used to inform diagnostic decision.  **Group 2: Usual care (n=30):**  Assessed by neurodevelopmental lead. If potential ADHD symptoms present, then also assessed by assistant mental health practitioner (questionnaires, developmental history and observation). Third assessment by neurodevelopmental lead for diagnostic decision. |
|  | Survey |  | **Participants:** 10 adolescent boys in a YOI who participated in the FACT trial in the QbTest group. Other than sex, no demographic characteristics are provided for this sample.  **Sampling strategy:** All 20 young people who completed QbTest in FACT trial invited to complete survey; 10 responded.  **Data collection:** “Qb Opinion Questionnaire” completed at 3 months. The survey contains 12 items e.g. “the QbTest results were difficult to understand” and the young person rates each item on a 5-point scale.  **Analysis:** Descriptive analysis | |
|  | Interviews with young people |  | **Participants:** 6 adolescent boys in a YOI who participated in the FACT trial in the QbTest group. Other demographic characteristics not reported for this sample.  **Sampling strategy:** Purposive sampling used to select people considering age, completion of QbTest and scores on the “Qb Opinion Questionnaire”. Unclear how many people were invited to participate in the interviews.  **Data collection:** Semi-structured interviews completed 3 months into the FACT trial, about acceptability of QbTest. At the time of interview, not all people had received the result of the test/ ADHD assessment.  **Analysis:** Thematic analysis, using inductive approach. | |
|  | Interviews with staff |  | **Participants:** 1 research assistant and 5 staff members from the YOI who used QbTest in the FACT trial. Demographic characteristics not reported for this sample.  **Sampling strategy:** All staff and the one researcher who used the QbTest in the trial were invited to participate.  **Data collection:** Semi-structured interviews completed at the end of the FACT trial, about the acceptability and feasibility of administering and implementing QbTest within usual practice, barriers and facilitators to use, and reasons for non-completion.  **Analysis:** Thematic analysis, using inductive approach. | |
| Hall (2016)(2) | Before-after study | **Study Location**  Kent, UK  **Language**  English  **Setting**  Community paediatric ADHD clinic  **Study design**  Uncontrolled before-after implementation study  **Funding**  Non-industry | **Population**  Children and adolescents diagnosed with ADHD in community paediatric clinic  **Sample selection and inclusion criteria**  Patient files selected using random number generator. Case notes included if case had received primary diagnosis of ADHD; for the post-test implementation evaluation cases were only included if they had received a QbTest was part of diagnostic assessment. If a file was excluded, next available file was selected.  **Exclusion Criteria**  Not reported  **Number participants included (analysed)**  80 (80)  **Age**  Pre-QbTest group: Mean 8.1; SD 2.4; Range 4.5-14.6  QbTest group: Mean 9.2; SD 2.3; Range 6.2-13.10  **Sex (% male)**  Pre-QbTest group: 80%; QbTest group: 70% | **Group 1 (pre-test implementation):** Standard ADHD assessment (n=40)  **Group 2 (post-test implementation):** QbTest (6-12) or QbTest (12-60) + standard ADHD assessment (n=40)  **Confounders**: authors state that “During this time period, there was no change to the assessment process, except the QbTest. Methods of acquiring  parent and teacher information, and the quantity and quality of information, remained unchanged, as did members of the clinical and administration team.” |
| Hollis (2018)(3, 4) | RCT | **Study Name**  AQUA trial  **Country**  England  **Language**  English  **Setting**  Secondary care/ community: 10 child and adolescent mental health services (CAMHS) or community paediatric clinics  **Study design**  RCT with embedded qualitative and survey components  **Funding**  Non-industry | **Population**  ADHD diagnosis in children aged 6-17 years  **Inclusion Criteria**  Children aged 6-17 years referred for their first ADHD assessment  **Exclusion Criteria**  Previous or current ADHD diagnosis; non-fluent in English; suspected moderate/ severe intellectual disability  **Number participants included (analysed)**  267 (250)  **Age**  QbOpen(n=123): Mean 9.5; range 6.0-17.4; SD 2.8  QbBlind (n=127): Mean 9.4; range 5.9-16.2; SD 2.8  **Sex (% male)**  **QbOpen:** 77%; **QbBlind:** 80%. | **Group 1: QbOpen (n=123):**  Usual care, in addition to QbTest (7-12 years) or QbTestPlus (12+ years), with Qb results shared with clinician to inform diagnostic decision, alongside clinical assessment.  Usual care varied between sites but typically included interview with child and their family, and one standardised informant-based behavioural assessment measure.  **Group 2: QbBlind (n =127):**  Same as Group 1, but QbTest/ QbTestPlus results were withheld from clinician. |
|  | Survey |  | **Participants:** 10 clinician leads (20% male) from each site in the AQUA trial, and 76 families from the AQUA trial. The following details were reported for the families only:   - Child mean age 10.2 years (SD 2.9; Range 7-18). - 79% male - Confirmed primary diagnosis - ADHD 46%; not ADHD 14%, unconfirmed 39%. Comorbidities – ASD 5%; Conduct Disorder and Oppositional Defiance Disorder 4%; Tourette’s/Tics 1%; Attachment Disorder 1%; Learning Difficulties 3%; Anxiety and Depression 1%.   **Sampling strategy:** All participants and the 10 lead clinicians from the trial invited to participate; 10 clinicians and 76 families responded.  **Data collection:** Quantitative online survey. Clinician questions centred on how best to administer QbTest, understanding results and communicating with families. Family questions focused on utility of QbTest in understanding symptoms and decisions, and experience of completing test.  **Analysis:** Descriptive analysis. | |
|  | Interviews with clinicians |  | **Participants:** 10 clinician leads (20% male) from each of the 10 sites involved in the AQUA trial.  **Sampling strategy:** The clinical lead for the AQUA trial at each of the 10 sites was invited to interview (all accepted).  **Data collection:** Semi-structured interviews conducted by a trained researcher regarding opinions of QbTest.  **Analysis:** Thematic analysis, using an inductive, reflexive approach. | |
|  | Interviews with families |  | **Participants:** 20 families from the AQUA trial (the main care-giver was the primary interviewee but where possible the young person was encouraged to participate with their parent – all young people had been in the “QbOpen” group). Sample characteristics:   - Child mean age 10.7 years (SD 2.9; Range 9-18). - 75% male - Confirmed primary diagnosis - ADHD 55%; not ADHD 25%, unconfirmed 25%. Comorbidities – ASD 5%; Conduct Disorder and Oppositional Defiance Disorder 0%; Tourette’s/Tics 5%; Attachment Disorder 0%; Learning Difficulties 0%; Anxiety and Depression 0%.   **Sampling strategy:** Two families per site who had participated in the AQUA trial “QbOpen” group were invited to interview. Thirty-eight families were invited to interview and 18 declined to participate. Refusing families were replaced with the next family until two families from each site were enrolled.  **Data collection:** Semi-structured interviews conducted by a trained researcher regarding opinions of QbTest.  **Analysis:** Thematic analysis using an inductive, reflexive approach. | |
| Humphreys (2018)(5) | Before-after study | **Study Location**  East Midlands, UK  **Language**  English  **Setting**  Community paediatric mental health settings in 3 NHS Trusts  **Study design**  Uncontrolled before-after implementation study, with survey component  **Funding**  Industry and non-industry: QbTech and East Midlands Academic Health Science Network | **Population**  Children and adolescents referred for ADHD assessment in community paediatric mental health settings  **Sample selection Inclusion Criteria**  Selection of children (method of selection not reported) referred for ADHD assessment in community paediatric mental health settings, before and after implementation of QbTest  **Exclusion Criteria**  Not reported  **Number participants included (analysed)**  Unclear - 20-30 cases before QbTest implementation and 20-30 cases after test implementation, from each of the three Trusts.  **Age**  5-16 years  **Sex (% male)**  Not reported | **Group 1 (pre-test implementation):** Standard ADHD assessment (60-90)  **Group 2 (post-test implementation):** QbTest (6-12) or QbTest (12-60) + standard ADHD assessment (n=60-90)  **Confounding factors**: none reported; authors note that the post-implementation group is after introduction of the QbTest and pathway re-design in two sites. |
|  | Survey to children and their families |  | **Participants:** 48 patients (children who had ADHD assessment using QbTest in CAMHS in the before-after study) and their families. Demographic characteristics not reported for this sample.  **Sampling strategy:** Surveys were distributed by clinic staff as paper version - 90 questionnaires distributed, 43% response rate (48 respondents).  **Data collection:** Survey on their experience of using QbTest (the same survey used in the AQUA trial.(4))  **Analysis:** Descriptive analysis. | |
|  | Survey to staff |  | **Participants:** Staff who had used QbTest (n= unknown). Demographic characteristics not reported for this sample.  **Sampling strategy:** Sampling strategy not reported. Number distributed not reported, 76% response rate.  **Data collection:** Survey on their experience of using QbTest (the same survey used in the AQUA trial.(4))  **Analysis:** Descriptive analysis. | |
| McKenzie (2022)(6) | Before-after study | **Study Name**  Focus ADHD  **Study Location**  England (sites throughout the country)  **Language**  English  **Setting**  CAMHS and paediatric sites (total of 20 sites)  **Study design**  Uncontrolled before-after implementation study, with qualitative and survey components  **Funding**  Industry and non-industry: QbTech and Academic Health Science Networks in England | **Population**  Children and adolescents referred for ADHD in CAMHS and paediatric sites  **Inclusion Criteria**  Selection of children referred for ADHD assessment in in CAMHS and paediatric sites across England, before and after implementation of QbTest. 61 potential sites identified; usable data obtained from 21 sites. Unclear how each site selected cases to report on. One site used test only for complex cases.  **Exclusion Criteria**  Not reported  **Number participants included (analysed)**  1098 cases - this consists of 549 (10-30 cases per site) before QbTest implementation and 549 (10-30 cases) after test implementation, from each of the 21 included sites.  **Age**  6-18 years  **Sex (% male)**  Not reported | **Group 1 (pre-test implementation):** Standard ADHD assessment (n=549)  **Group 2 (post-test implementation):** QbTest (6-12) or QbTest (12-60) + standard ADHD assessment (n=549)  **Subgroups**:  CAMHS vs Paediatric sites  Also report stratified data based on number of cases referred per site, and large vs small test volume – stratified data not extracted for these  **Confounding factors**: QbTest implementation occurred from April 2019 to March 2022 and so overlaps with COVID-19 pandemic (from March 2020) |
|  | Survey to HCPs |  | **Participants:** 65 HCPs who attended audit training in the Focus ADHD study. Demographic characteristics not reported for this sample.  **Sampling strategy:** All HCPs who attended audit training in the Focus ADHD study invited (n=unknown), 65 responded.  **Data collection:** Online survey about how best to administer the QbTest, understanding the results and communicating with families.  **Analysis:** Descriptive analysis. | |
|  | Survey to patients |  | **Participants:** 22 patients who had been assessed with QbTest (and their parents) in the Focus ADHD study. Demographic characteristics not reported for this sample.  **Sampling strategy:** Survey distributed to all patient families via text/ email and clinicians/ key stakeholders asked to pass it on (n=unknown). 22 patients/ families responded.  **Data collection:** Online survey about the utility of the QbTest in understanding symptoms and diagnostic decisions and the experience of completing the test.  **Analysis:** Descriptive analysis. | |
|  | Interview |  | **Participants:** 21 healthcare staff involved in implementation of QbTest at their site, or conducting the test/ interpreting test results, in the Focus ADHD study. Demographic characteristics not reported for this sample.  **Sampling strategy:** All sites were invited to participate - they aimed to include participants with different roles in the test implementation process, including those who delivered the test, interpreted the test and managers who were responsible for implementing the test at their site.  **Data collection:** Semi-structured interviews conducted to explore experience of using test, adoption of test at their site and sustainability of its use.  **Analysis:** Thematic analysis, analysed using the non-adoption, abandonment, scale-up, spread, sustainability (NASS) framework. | |
| Pellegrini (2020)(7) | Survey | **Study Name**  Not reported  **Study Location**  Ireland  **Language**  English  **Setting**  Irish Child and Adolescent Mental Health Services (CAMHS) – 3 CAMHS teams  **Study design**  Mixed methods study of real-world impact of test implementation  **Funding**  Not funded  **CPT**  QbTest + standard ADHD assessment | **Participants:** 50 participants: 17 clinicians who had used QbTest in one of the three CAMHS involved in the study, 15 young people who had completed QbTest as part of ADHD assessment in one of the three CAMHS teams involved in the study, and their parent/guardians (n=18). Demographic characteristics not reported for this sample.  **Sampling strategy:** Young people and their parents/guardians were recruited during ADHD assessment – the clinician made the family aware of the survey study. Clinicians were sent the survey via email by research staff. Number of people invited to participate not reported.  **Data collection:** Quantitative survey on experience of using QbTest. The survey was based on a template provided by QbTech that had been used in the AQUA qualitative sub-study.(4)  **Analysis:** Descriptive analysis. | |
|  | Focus groups |  | **Participants**: 19 clinicians who were working in one of the three CAMHS teams selected for this research in Ireland, and who were involved in using the Qbtest as part of an ADHD assessment process. Professional disciplines included: administration, occupational therapy, nurses, psychology, psychiatry, social work and speech and language therapy. Demographic characteristics not reported for this sample.  **Sampling strategy:** All clinicians in the study who were using QbTest were invited (n=unknown).  **Data collection:** Three semi-structured focus groups were conducted (n=6; n=6; n=7), gathering information on their experiences with the QbTest.  **Analysis:** Thematic analysis, using a six-step, reflexive process. | |
| Sharma (2022)(8) | Before-after study | **Study Location**  Swindon, UK  **Language**  English  **Setting**  Hospital paediatric clinic  **Study design**  Uncontrolled before-after implementation study  **Funding**  Not reported | **Population**  Children and adolescents referred for ADHD assessment in hospital paediatric clinic  **Sample selection and inclusion Criteria**  Patients assessed for ADHD between Jul 2020-Jan 2022 in hospital paediatric clinic, who had been referred for ADHD/ non-specific behavioural problems/ ASD. Unclear how patients were selected  **Exclusion Criteria**  Any patient who had not completed an ADHD assessment in the timeframe or whose assessment resulted in inconclusive determination.  **Number participants included (analysed)**  40 (40)  **Age**  All participants: Mean 11.7 (SD 2.4)  **Sex (% male)**  Not reported | **Group 1 (pre-test implementation):** Standard ADHD assessment (n=20)  **Group 2 (post-test implementation):** QbTest (6-12) or QbTest (12-60) + standard ADHD assessment (n=20)  **Subgroups:**  ADHD cases – those referred for ADHD  Complex cases – those originally referred for non-specific behavioural difficulties or ASD  **Confounding factors**: none reported |
| Ulberstadt (2020)(9) | Survey | **Study Name**  Not reported  **Study Location**  Germany, Sweden, USA  **Language**  English  **Setting**  Secondary care  **Study design**  Survey data from two-gate DTA study  **Funding**  Industry - authors employed by QbTech  **CPT**  QbCheck | **Participants:** Patients (adolescents/ adults) who used QbCheck in the DTA study and who completed the survey (n=125; 59 ADHD and 69 healthy controls). Demographic characteristics not reported for this sample.  **Sampling strategy:** All patients (142) from DTA study given survey, 125 completed it.  **Data collection:** Survey about experience of using QbCheck – three questions assessed on a scale from 0 to 10 that assessed the usability of the test; one yes/no question about problems with using the test.  **Analysis:** T tests (the dimensional variables) or chi-square test (the categorical variable) to compare the group with ADHD to the controls. | |
| Vogt (2011)(10) | Before-after study | **Study Location**  Berkshire, UK  **Language**  English  **Setting**  Child and adolescent mental health services (CAMHS)  **Study design**  Uncontrolled before-after implementation study  **Funding**  Not reported | **Population**  Children and adolescents referred for ADHD assessment in CAMHS  **Sample selection and inclusion criteria**  Notes of 108 patients referred for ADHD to CAMHS clinic over 2 year period – 1 year before (2006-7) and 1 year after implementation of QbTest (2007-2008). Unclear whether all children assessed during eligible time periods enrolled or selected sub-sample.  **Exclusion Criteria**  Not reported  **Number participants included (analysed)**  108 (108)  **Age**  Pre-QbTest group: Mean 9; mode 10; median 9  QbTest group: Mean 10.5; mode 8; median 10  **Sex (% male)**  Not reported | **Group 1 (pre-test implementation):** Standard ADHD assessment (n=46)  **Group 2 (post-test implementation):** QbTest (6-12) or QbTest (12-60) + standard ADHD assessment (n=62)  **Confounders**: same child and adolescent psychiatrists conducted the assessments for both groups using the same protocol. |
| Williams (2021)(11) | RCT | **Study Name**  QUOTA  **Country**  England  **Language**  English  **Setting**  Secondary care/ community: 5 CAMHS or community paediatric clinics  **Study design**  Parallel group, single-blind, feasibility multi-site RCT with embedded qualitative and survey components  **Funding**  Non-industry | **Population**  ADHD medication management for people aged 6-15  **Inclusion Criteria**  6-17 years; referred to CAMHS/ community paediatric; clinical ADHD diagnosis; about to commence ADHD medication (methylphenidate/ lisdexamfetamine)  **Exclusion Criteria**  Non-fluent English; unable to provide written consent; suspected severe learning disability.  **Number participants included (analysed)**  44 (44)  **Age**  Mean (SD): QbTest: 9.29 (2.81); Control: 9.22 (2.19). Full sample range: 6-15 years.  **Sex (% male)**  **QbTest -** 95.24%. **Control -** 82.61% | **Group 1: QbTest + treatment as usual (n=21):**  Treatment as usual, in addition to QbTest completed at baseline, 2-4 weeks later (follow-up 1) and 8-12 weeks later (follow-up 2). At each time point, the clinician reviewed QbTest results with other clinical tools to monitor medication.  Treatment as usual varied between sites. Participants received their site’s standard usual care, but all sites were asked to contact participant twice by the end of the 12 weeks (to ensure level of contact consistent between groups).  **Group 2: Treatment as usual (n=23):**  Treatment as usual was as listed for Group 1 (usual care, without QbTest). |
|  | Survey |  | **Participants:** Clinicians who took part in the feasibility RCT (n=unclear). Demographic characteristics not reported for this sample.  **Sampling strategy:** All clinicians invited to take part.  **Data collection:** Clinician pro forma to record data on diagnoses and clinical decision-making along with resources used to reach decisions.  **Analysis:** Descriptive analysis. | |
|  | Interviews with parents |  | **Participants:** Parents of 8 children aged 6-15 years (diagnosed with ADHD and referred to CAMHS/ community pediatric clinic in the UK to commence ADHD medication), who had participated in the feasibility RCT. Demographic characteristics not reported for this sample.  **Sampling strategy:** All participants invited to take part in interviews.  **Data collection:** Interviews conducted to explore the experience of the study and the acceptability of the QbTest.  **Analysis:** Thematic analysis using inductive and deductive approaches. | |
|  | Interviews with clinicians |  | **Participants:** 5 clinicians who took part in the feasibility RCT. Demographic characteristics not reported for this sample.  **Sampling strategy:** All clinicians invited to take part in interviews.  **Data collection:** Interviews conducted to explore the experience of the study, the acceptability of the QbTest, and the potential future implementation of the QbTest.  **Analysis:** Thematic analysis using inductive and deductive approaches. | |

## Progress-Plus characteristics for studies that reported at least one Progress-Plus item

| **Study Details*** | **Progress Plus Item** | **Details for main study** | **Details for survey component** | **Details for interview component** |
| --- | --- | --- | --- | --- |
| **Chitsabesan (2022)(1)** | Age - Percentages in each age category | **QbTest (n=30)**: age 16: 20%; a17: 26.7%; age 18: 50%; missing: 3.3%. **Usual care (n=30)**: age 16: 10%; age 17: 36.7%; age 18: 53.3%; missing: 0%. | NR | NR |
|  | Sex (% male) | 100% | **Survey to adolescents:** 100% | **Interviews with adolescents:** 100% |
|  | Ethnicity | **QbTest (n=30)**: White 76.7%; Other 20%; Missing 3.3%. **Usual care (n=30)**: White 80%; Other 20%; Missing 0%. | NR | NR |
|  | Education | **QbTest (n=30):** Mainstream 20%; Pupil referral unit 10%; None 66.7%; Other 0%; Missing 3.3%. **Usual care (n=30)**: Mainstream 20%; Pupil referral unit 16.7%; None 56.7%; Other 6.7%; Missing 0%. | NR | NR |
|  | Time-Dependent Relationships | All participants in youth justice system | NR | NR |
| **Hollis (2018)(3)** | Age - Mean (SD; range) | **QbOpen:** 9.5 (2.8; 6.0-17.4); **QbBlind:** 9.4 (2.8; 5.9-16.2) | **Survey to clinician leads:** Not reported  **Survey to families:** 10.2 years (2.9; 7-18 years) | **Interviews with clinician leads:** Not reported  **Interviews with families:** 10.7 years (2.9; 9-18 years) |
|  | Sex (% male) | **QbOpen:** 77%; **QbBlind:** 80%. | **Survey to clinician leads:** 20%  **Survey to families:** 79% | **Interviews with clinician leads:** 20%  **Interviews with families:** 75% |
|  | Ethnicity (% white, mixed, other) | **QbOpen** (data from 83/123 participants): White 88%; Mixed and other 12%. **QbBlind** (89/127 participants): White 90%; Mixed and other 10%. | NR | NR |
|  | Neurodevelopmental/learning disorders | **Diagnoses (n=241; allows more than one diagnosis per patient):** 71% ADHD; 35% oppositional defiant disorder/ conduct disorder; 20% any anxiety disorder; 17% chronic tic disorder/ Tourette syndrome; 9% autism spectrum disorder; 3% depressive disorder; 11% learning difficulties; 0.4% attachment disorder; 19% no psychiatric diagnoses. | **Survey to clinician leads:** Not reported  **Survey to families:** ASD 5%; Conduct Disorder and Oppositional Defiance Disorder 4%; Tourette’s/Tics 1%; Attachment Disorder 1%; Learning Difficulties 3% | **Interviews with clinician leads:** Not reported  **Interviews with families:** ASD 5%; Conduct Disorder and Oppositional Defiance Disorder 0%; Tourette’s/Tics 5%; Attachment Disorder 0%; Learning Difficulties 0%; |
|  | Mental health conditions | NR | **Survey to clinician leads:** Not reported  **Survey to families:** Anxiety and Depression 1% | **Interviews with clinician leads:** Not reported  **Interviews with families:** Anxiety and Depression 0%. |
| **Hall (2016)(2)** | Age – Mean (SD; range) | **Pre-QbTest group:** Mean 8.1; SD 2.4; Range 4.5-14.6. **QbTest group:** Mean 9.2; SD 2.3; Range 6.2-13.10 | NA | NA |
|  | Sex (% male) | **Pre-QbTest group:** 80%; **QbTest group:** 70%; | NA | NA |
|  | Neurodevelopmental/learning disorders | **No. participants with secondary diagnosis - Pre-QbTest group:** ASD 6; ASD and tic disorder 2; ASD and dyspraxia 2; ASD and OCD 1; oppositional defiance disorder 1; sensorineural deafness 1; mild epilepsy 1; Tourette’s syndrome 1. **QbTest group:** ASD 7, Tourette’s syndrome 1, sensory processing disorder 1, mild speech and language disorder 1, emotional difficulties1, dyslexia 1, learning difficulties 1. | NA | NA |
| **Vogt (2011)(10)** | Age – Mean, mode, median | **Pre-QbTest group:** Mean 9; mode 10; median 9. **QbTest group:** Mean 10.5; mode 8; median 10 | NA | NA |
| **Humphreys (2018)(5)** | Age – Range | **Full sample:** 5-16 years | NR | NA |
| **McKenzie (2022)(6)** | Age – Range | **Full sample:** 6-18 years | NR | NR |
| **Williams (2021)(11)** | Age – Mean (SD); full sample range | **QbTest:** 9.29 (2.81); **Control:** 9.22 (2.19). **Full sample range:** 6-15 years. | NR | NR |
|  | Ethnicity | **QbTest -** White 76.19%; Bangladeshi 4.76%; Dual heritage 4.76%; Not given 4.76%; Other 4.76%; Pakistani 4.76%. **Control -** White 91.30%; Bangladeshi 0%; Dual heritage 0%; Not given 0%; Other 4.35%; Pakistani 4.35% | NR | NR |
|  | Neuro-developmental | **QbTest** – ASD/ social communication/ speech/ speech difficulties 14.28%; Attachment disorder 0%; Conduct disorder 0%; Tic and neurological disorders 9.52%; Mood disorders 4.76%. **Control -** ASD/ social communication/ speech/ speech difficulties 21.75%; Attachment disorder; 4.35% Conduct disorder 8.70%; Tic and neurological disorders 0%; Mood disorders 0%. | NR | NR |
| **Sharma (2022)(8)** | Age – Mean (SD) | **All participants:** Mean 11.7 (SD 2.4) | NA | NA |

*Two studies reported no Progress-Plus criteria (Pellegrini 2020 and Ulberstadt 2020). NR = not reported. NA = not applicable.

## Quality assessments

### ROBINS-I assessment for before-after studies

| **Study Details** | **Domain*** | | | | | | | | **Rationale** |
| --- | --- | --- | --- | --- | --- | --- | --- | --- | --- |
|  | **1** | **2** | **3** | **4** | **5** | **6** | **7** | **Overall** |  |
| Hall(2016)(2) | ☹ | ☺ | ☺ | ☺ | 😐 | 😐 | 😐 | ☹ | Confounders not controlled for and potential for confounding of the effect of the intervention; only people who had final diagnosis within timeframe selected; outcome measure could have been influenced by knowledge of intervention received; no protocol. Note: selection of participants was random, hence exclusion of participants was covered under the missing data domain. |
| Sharma(2022)(8) | ? | ☹ | ☺ | ? | ☺ | 😐 | 😐 | ☹ | Conference abstract with no information about whether confounders were controlled for, or about bias due to deviations from intended interventions; participants excluded if assessment inconclusive or did not receive diagnosis in timeframe; outcome measure could have been influenced by knowledge of intervention received; no protocol. |
| Humphreys(2018)(5) | ☹ | ? | ☺ | 😐 | ? | 😐 | 😐 | ☹ | Confounders not controlled for and potential for confounding of the effect of the intervention; no information about participant selection; potential for bias due to deviations from intended interventions due to two sites having a pathway redesign after introduction of QbTest; no information about missing data (authors confirmed not only ADHD+ cases selected); outcome measure could have been influenced by knowledge of intervention received; no protocol. |
| McKenzie(2022)(6) | ☹ | ? | ☺ | ☺ | ☺ | 😐 | 😐 | ☹ | Confounders not controlled for and potential for confounding of the effect of the intervention (COVID-19 only confounder mentioned which the authors say would have impacted on the analysis); little information on participant selection; outcome measure could have been influenced by knowledge of intervention received; no protocol. |
| Vogt(2011)(10) | ☹ | ? | ☺ | ☺ | ☺ | 😐 | 😐 | ☹ | Confounders not controlled for and potential for confounding of the effect of the intervention; no information about participant selection; outcome measure could have been influenced by knowledge of intervention received; no protocol. |

*1: Confounding (potential confounders for all studies: Age at the point of seeking referral for ADHD; Sex; Comorbidities - e.g. Autism, anxiety; Nature and severity of symptoms at presentation – e.g. predominantly inattentive or hyperactive; Socioeconomic status; Ethnicity and for McKenzie (2022) also COVID-19 pandemic); 2: Selection of participants; 3: Classification of interventions; 4: Deviations from intended interventions; 5: Missing data; 6: Measurement of the outcome; 7: Selection of the reported result.

Note: Sad face= serious risk of bias; smiling face= low risk of bias, question mark= no information.

### RoB2 assessment for randomised controlled trials

| **Study Details** | **Outcome** | **Domain** | | | | | | **Rationale** |
| --- | --- | --- | --- | --- | --- | --- | --- | --- |
|  |  | **1** | **2** | **3** | **4** | **5** | **Overall** |  |
| Hollis(2018)(3) | Diagnostic decision (confirming or excluding ADHD diagnosis) made  OR 2.43 (1.34-4.39) | ☺ | ☺ | ☺ | ☺ | ☺ | ☺ | Appropriate randomisation and allocation concealment; participants blinded to allocation, clinicians not blinded, but it seems unlikely deviations took place due to trial context; appropriate measurement of the outcomes; pre-registered protocol, however potential for selective reporting due to HRQoL pre-specified but data not reported.  Outcome not impacted by censoring/withdrawals |
|  | Diagnostic status | ☺ | ☺ | ☺ | ☺ | ☺ | ☺ | Outcome not impacted by censoring/withdrawals |
|  | Diagnostic confidence | ☺ | ☺ | ☺ | ☺ | ☺ | ☺ | Outcome not impacted by censoring/withdrawals |
|  | Stability of diagnosis | ☺ | ☺ | ☺ | ☺ | ☺ | ☺ | Outcome not impacted by censoring/withdrawals |
|  | No. consultations to diagnostic decision | ☺ | ☺ | ☹ | ☺ | ☺ | ☹ | Large proportion of participants (80/250) were censored from the analysis as they dropped out or were discharged from the clinic and so did not have a diagnosis at 6 months. This was a particular problem for time-to-event outcome data where the analysis assumed that participants were uninformatively censored and so had equivalent outcomes to those for whom full follow-up data were available. |
|  | Number of minutes spent at clinic appointments until diagnosis | ☺ | ☺ | ☹ | ☺ | ☺ | ☹ |  |
|  | Number of clinic appointments until diagnosis | ☺ | ☺ | ☹ | ☺ | ☺ | ☹ |  |
|  | Number of days to diagnostic decision | ☺ | ☺ | ☹ | ☺ | ☺ | ☹ |  |
|  | Cost of clinic appointments | ☺ | ☺ | ? | ☺ | ☺ | ? | Unclear how costs calculated and so not clear how censored individuals contributed to this outcome |

1: Randomisation process; 2: deviation from intended intervention; 3: missing outcome data; 4: measurement of the outcome; 5: selective outcome reporting

### QSSP quality assessment for studies that reported survey data

| **Questions (n=20)** | **Quality assessment answers per study (answer options: yes, no, not stated clearly)** | | | | | | |
| --- | --- | --- | --- | --- | --- | --- | --- |
|  | **Chitsabesan (2022)(1)** | **Hollis (2018)(4)** | **McKenzie (2022)(6)** | **Pellegrini (2020)(7)** | **Humphreys (2018)(5)** | **Ulberstadt (2020)(9)** | **Williams (2021)(11)** |
| Was the problem or phenomenon under investigation defined, described, and justified? | Yes | Yes | Yes | Yes | Yes | Yes | Yes |
| Was the population under investigation defined, described, and justified? | Yes | Yes | Yes | Yes | Yes | Yes | Yes |
| Were specific research questions and/or hypotheses stated? | Yes | Yes | Yes | Yes | Yes | Yes | Yes |
| Were operational definitions of all study variables provided? | Yes | Yes | Yes | Yes | Yes | Yes | Yes |
| Were participant inclusion criteria stated? | Yes | Yes | Yes | Yes | Yes | Yes | Yes |
| Was the participant recruitment strategy described? | Yes | Yes | Yes | Yes | Yes | Yes | Yes |
| Was a justification/ rationale for the sample size provided? | No | No | No | No | No | No | No |
| Was the attrition rate provided?  (applies to cross-sectional and prospective studies) | Yes | Yes | Yes | Yes | No | Yes | Yes |
| Was a method of treating attrition provided? (applies to cross-sectional and prospective studies) | No | Yes | No | No | No | No | Yes |
| Were the data analysis techniques justified (i.e., was the link between hypotheses/ aims / research questions and data analyses explained)? | Yes | Yes | Yes | Yes | Yes | Yes | Yes |
| Were the measures provided in the report (or in a supplement) in full? | Yes | Yes | Yes | Yes | Yes | Yes | No |
| Was evidence provided for the validity of all the measures (or instrument) used? | No | No | No | No | No | No | No |
| Was information provided about the person(s) who collected the data (e.g., training, expertise, other demographic characteristics)? | Yes | Yes | Yes | Yes | Yes | Yes | Yes |
| Was information provided about the context (e.g., place) of data collection? | Yes | Yes | Yes | Yes | Yes | Yes | Yes |
| Was information provided about the duration (or start and end date) of data collection? | No | Yes | No | No | No | No | No |
| Was the study sample described in terms of key demographic characteristics? | No | Yes | No | No | No | No | Yes |
| Was discussion of findings confined to the population from which the sample was drawn? | Yes | Yes | Yes | Yes | Yes | Yes | Yes |
| Were participants asked to provide (informed) consent or assent? | Yes | Yes | Yes | Yes | Not stated clearly | Not stated clearly | Yes |
| Were participants debriefed at the end of data collection? | Not stated clearly | Not stated clearly | Not stated clearly | Yes | Not stated clearly | Not stated clearly | Not stated clearly |
| Were funding sources or conflicts of interest disclosed? | Yes | Yes | Yes | Yes | Yes | Yes | Yes |

### CASP checklist quality assessment of studies that reported qualitative data

| **CASP Checklist Questions** | **Quality assessment answers per study (answer options: yes, no, can’t tell)** | | | | |
| --- | --- | --- | --- | --- | --- |
|  | **Chitsabesan (2022)(1)** | **Hall(2017)(4)** | **McKenzie (2022)(6)** | **Pellegrini (2020)(7)** | **Williams (2021)(11)** |
| Was there a clear statement of the aims of the research? | Yes | Yes | Yes | Yes | Yes |
| Is a qualitative methodology appropriate? | Yes | Yes | Yes | Yes | Yes |
| Was the research design appropriate to address the aims of the research? | Yes | Yes | Yes | Yes | Yes |
| Was the recruitment strategy appropriate to the aims of the research? | Yes | Yes | Yes | Yes | Yes |
| Was the data collected in a way that addressed the research issue? | Yes | Yes | Yes | Yes | Yes |
| Has the relationship between researcher and participants been adequately considered? | Can’t tell | Yes | Can’t tell | Yes | Can’t tell |
| Have ethical issues been taken into consideration? | Yes | Yes | Yes | Yes | Yes |
| Was the data analysis sufficiently rigorous? | Can’t tell | Yes | Can’t tell | Yes | Yes |
| Is there a clear statement of findings? | Yes | Yes | Yes | Yes | Yes |

## Results

### Interview and survey data

#### Views around the helpfulness of the QbTest

Themes identified regarding views around the helpfulness of the QbTest included contribution to ADHD diagnosis, treatment decision making, communication with caregivers, and understanding of subjective experience.

##### Contribution to ADHD diagnosis

###### Findings from qualitative data

Clinicians interviewed in the qualitative sub-study of the AQUA RCT reported that use of the QbTest led them to feel more confident in their diagnostic decision making.(4)

*“I would move to the diagnosis more confidently and more quickly having evidence that something was wrong, you know objective evidence. …reduced the amount of the anxiety of uncertainty” - Healthcare professional on the use of QbTest(4)*

Increased confidence in the diagnostic decision was also reported in interviews with healthcare staff in the Focus ADHD study, who commented that the increased confidence was derived from the fact that the data provided by the test is objective, rather than scales and surveys that give subjective data.(6) Focus groups with clinicians in CAMHS also revealed that the QbTest gave them increased confidence in their decisions.(7)

*“I think it gives all clinicians a bit more confidence around making diagnosis, and I think for nurses, that’s where its particularly helpful. Especially if they’re nurse prescribers, because they have that responsibility of making the diagnosis and providing medication. So, they want it to be… they want to feel very, very sure that this is ADHD, that nothing is being missed.” – Healthcare professional on using QbTest(6)*

Despite the suggestion from studies that the QbTest could contribute positively to the ADHD diagnostic process, clinicians reported in focus groups that there is a need to establish where the QbTest falls on the ADHD assessment pathway.(7) Staff interviewed in the Focus ADHD study felt that the QbTest should be implemented early in the assessment pathway, and when this was done, the clinicians felt they had a clearer understanding of whether the young person had a profile indicative of ADHD.(6) In line with this, most clinicians and families interviewed in the AQUA sub-study felt that the QbTest should be conducted before the initial appointment with clinician. One family suggested doing QbTest in GP surgery as initial screen and clinicians were supportive of this. Whilst, some clinicians suggested using it only for complex cases.(4)

*“I would then also even put a QbTest in as a precursor to the initial consultation so that at the time you see the child, they’ve had all the relevant questionnaires completed from home and school and a QbTest and you could probably make a diagnosis on the first appointment” - Healthcare professional on the use of QbTest(4)*

Some clinicians and families interviewed in the AQUA sub-study questioned the validity of the clinical setting of the test and wondered if it is not representative of what happens e.g. in school.

*“He behaves differently at home and school to what he would do in a clinical office sort of thing… And of course for that twenty minutes that he was seen he was on his best behaviour” – Healthcare professional on the use of QbTest(4)*

###### Findings from survey data

Some respondents to the patient/ carer survey in the Focus ADHD study said that they think the QbTest should have been offered sooner.(6)

##### Diagnosis in complex cases

###### Findings from qualitative data

Interview findings from two studies suggested that the QbTest can be helpful in the diagnosis of individuals with comorbidities.(1, 4) Clinicians interviewed in the AQUA RCT sub-study reported that the tests helped to discriminate ADHD from Autism Spectrum Disorder (ASD), anxiety, depression, and learning difficulties. Clinicians with more prior experience of using the QbTest were more positive in its abilities to help in the diagnosis of cases with co-morbidities than those with less experience.(12) In the FACT RCT, one staff member interviewed also said that the QbTest was helpful in the assessment of young people where there might be concerns about co-morbid diagnosis.(1)

*“I very often use it for children that I suspect have got ASD comorbidity. I think it’s very clear that there’s a group of children with just pure ADHD who do a QbTest in one way, and then the group that’s got some degree of autism or autistic traits do it very differently, and I think that’s really helpful”- Healthcare professional on the use of QbTest(4)*

In the Focus ADHD study, interviews with staff also found that the QbTest can be helpful in cases where there is contradictory information between home and school settings, or cases where the young person has limited corroborating information due to being home schooled or a ‘looked after child’.(6)

*“I think it works well with subtle presentations. Presentations maybe where there’s a disagreement between school and home. Cases where there are parental disagreements. Cases where young people themselves are unsure.” – Healthcare professional on the use of QbTest(6)*

Clinicians interviewed in the AQUA RCT sub-study reported that the QbTest was useful in differentiating ADHD subtypes, but there was no consensus as to which symptom domain was particularly valuable. Some clinicians specifically commented on the utility of the attention measure for girls with the inattentive subtype who can be hard to diagnose.(4) This was also highlighted in the Focus ADHD study - healthcare staff commented that the addition of the QbTest into the assessment process helped to identify individuals with subtle presentation of ADHD (e.g. girls or older adolescents) and those who mask their difficulties.(6)

*“I think it can be helpful for picking out cases where there might be more subtle presentations, for example in girls or older adolescents.” – Healthcare professional on the use of QbTest(6)*

###### Findings from survey data

In two studies that surveyed healthcare professionals, there was no consensus as to whether the QbTest should be reserved for use in cases where there is a diagnostic uncertainty.(5, 6) However, in one of the studies,(6) some healthcare professionals did report that the test was most useful in certain patient groups including female patients, older children, cases where the parent or school does not agree with the clinician’s decision, and in identifying patients for ASD assessment by being able to rule out ADHD. Survey data from healthcare staff in the Focus ADHD study concurred with the interview findings from this study; healthcare staff reported that the QbTest is useful in those with subtle presentations who mask their symptoms.(6)

##### Time to diagnostic decision

###### Findings from qualitative data

Qualitative data (mostly from healthcare professionals) from four studies suggested that the QbTest could be helpful in improving the time to diagnostic decision. Clinicians interviewed in the AQUA sub-study reported that the QbTest may help to reduce delays in diagnosis and treatment onset. They also noted that time and cost savings may be made by replacing the lengthy and difficult to access school observations with the QbTest.(4)

*“What we did was because of QbTest results, I then stopped the school observations, so then we could confirm the diagnosis and go ahead with the medication”- Healthcare professional on the use of QbTest(4)*

Families interviewed in the AQUA trial commented that there is a need for a quick decision to facilitate treatment initiation, particularly for children who were struggling in education, and to not prolong the emotionally overwhelming process. However, they also emphasized that they did not want the process to be rushed, and their child should not be “labelled” quickly.(4)

*“I just wished it were more like I say I was in and out, just wished it were more appointments and a bit more time” - Parent of child who had used QbTest(4)*

Staff interviewed in the FACT RCT also felt that the QbTest could help to improve waiting times.(1) Focus groups with 19 clinicians who had used the QbTest in CAMHS highlighted that the QbTest was perceived to have resulted in time savings and felt that it has the potential to streamline and improve the service.(7)

*“…so on the ground level it’s helping us with our picture of the child, but in the bigger picture of things, if we are dealing more efficiently and more correctly with each child, that’s going to make the service more efficient and better for the next child coming in the door, so there’s a bigger picture knock on effect happening with a tool like this…” - Healthcare professional on the use of QbTest(7)*

These views were shared by some healthcare staff interviewed in the Focus ADHD study, who felt that the addition of the QbTest into the assessment process led to a faster and more efficient process, which in turn reduces cost.(6) Most sites in the Focus ADHD study found that QbTest implementation had resulted in fewer appointments by replacing the school observation, and that the quicker assessment pathway supported the young person in getting educational support quickly. Some sites also reported a reduction in re-referrals from caregivers who disagreed with a non-diagnosis decision.

*“I see it as a way of reducing the amount of time children are waiting to be seen. And thus, reducing the number of follow-ups, thus reducing the number of times they have to come back to the hospital so it’s an opportunity to save the patients and parents time.” - Healthcare professional on the use of QbTest(6)*

###### Findings from survey data

Some patients/ carers (n=not reported) surveyed in the Focus ADHD study reported that the QbTest helped to speed up the assessment process and to get a diagnosis.(6)

##### Treatment decision making

###### Findings from qualitative data

Clinicians reported that they appreciated the objectivity of the QbTest in comparison to informant measures traditionally used to monitor medication, particularly in complex cases (no quote provided).(11)

Clinicians interviewed in the AQUA RCT qualitative sub-study reported greater support from parents on initiating and continuing medication, and greater adherence to medication, as a result of being able to directly observe the effect of medication with a QbTest.(4)

*“They can see there is a difficulty there and that the medication can improve that, I think it does really improve adherence and understanding of what the difficulties are for the kids more than anybody else” - Healthcare professional on the use of QbTest(4)*

Clinicians in the QUOTA trial also reported that being able to directly observe medication effect with the QbTest led to greater support from parents on accepting treatment recommendations.(11) They felt that the QbTest increases parental confidence in treatment decision making and helps communication around treatment.(11) In line with this, families interviewed in the AQUA RCT reported that seeing the QbTest results made them more confident that the medication would help their child.(4)

*“It’s a big decision to allow your children to have these drugs, as it were. So, again, seeing those results made me more confident that yes the medication would help him” – Parent of child who had used QbTest(4)*

Interviews with healthcare staff in the Focus ADHD study identified that the QbTest could be helpful in dose titration and checking medication utility, and the staff felt that the QbTest helped young people/ caregivers to understand medication decisions and the effects of the medication. This study only involved interviews with staff, not patients/ carers.(6)

*“We get a lot of people of adolescent age that say ‘oh I don’t want my meds’ and have come off the meds and then deteriorated, and actually if you can do those baseline results with them, I think it will just give us something evidence-base to start having those conversations of ‘actually do you need medication/do you not need medication/what are the benefits that medication bring?” -Healthcare professional on the use of QbTest(6)*

###### Findings from survey data

Survey data from two studies suggested that patients/ caregivers were not convinced that the results of the QbTest helped them to understand medication decisions.(1, 4) Less than half (20/52) of families surveyed in the AQUA RCT felt that it helped them to understand the decisions made about medication, although it is notable that most participants did not commence medication, so the results are difficult to interpret.(4) Likewise, in the FACT RCT, there was no consensus among 10 adolescent boys assessed for ADHD as to whether the QbTest results helped them to understand how the decisions about medication had been made (the majority voted “neither agree/ disagree”.(1)

Some healthcare professionals in the Focus ADHD felt that the QbTest helped them to decide how effective the medication is, and has increased their confidence in decision making about treatment.(6) Most clinicians surveyed in the QUOTA trial felt that the QbTest was helpful in determining treatment (more-so at follow-up 1 than follow-up 2).(11) In contrast, in a survey to clinicians in CAMHS (n=not reported), However, in a survey of clinicians in CAMHS (sample size not reported), only 30% of respondents agreed that the QbTest results influenced treatment decisions (60% of respondents remained neutral and 10% strongly disagreed).(5)

##### Communication with caregivers

###### Findings from qualitative data

Interview findings suggested that the QbTest helped to improve communication between clinicians and patients/ families. Clinicians and families interviewed in the AQUA RCT sub-study felt that the output of the QbTest helped them to communicate to families information around diagnosis and medication effect. Specifically, clinicians reported that being able to show a comparison of the child’s performance to a normative sample helped them to communicate the diagnostic decision to families, and they thought that this helped families to accept the decision.

*“A lot of parents who previously would have probably shouted and screamed at you for not saying their child had ADHD will accept it if the computer is not showing the evidence” – Healthcare professional on using QbTest(4)*

Mostly, families in the AQUA sub-study felt that clinicians explained the QbTest reports well and they were easy to understand, however some families felt that it was unclear how the report was being used to inform decision making.(4)

*“I don’t know if she explained, it felt like the QbTest had said it so that’s what we’re going with” – Parent of child who had used QbTest(4)*

In two other studies, clinicians also felt the QbTest helped to improve communication with young people and their families, through improving clarity,(7) and through providing an objective and visual aspect to use to evidence and justify diagnostic decisions.(6) However, some clinicians in the latter study (Focus ADHD) commented that families could still struggle to accept a diagnostic decision.(6) This study did not interview parents/ carers.

*“I think they offer a very visual result for the parents, […] especially the little chart that shows hyperactivity and stillness and the wild swinging round. So, I think that sort of aspect to it is really good to be able to communicate the diagnosis.” – Healthcare professional on use of QbTest(6)*

Findings also suggested that the implementation of the QbTest can help to improve communication between the clinician and school,(4) between clinical colleagues,(7) and between the person with ADHD and their family(4).

###### Findings from survey data

Survey data suggested that clinicians valued the QbTest for improving communication with the patient/ family. In line with the results from the AQUA sub-study interviews, all 10 clinicians surveyed reported that the QbTest helped to improve communication with patients and they all valued the QbTest in helping to explain why they had ruled out a diagnosis.(4) Likewise, the majority of clinicians surveyed in three other studies felt that the QbTest results improved the communication of diagnostic decision with the patient.(5-7)

However, the views of parents/ carers were more mixed as to whether the QbTest improved communication. Only 31/68 families in the AQUA sub-study said that the QbTest helped them to understand how the diagnosis was made, and answers were split regarding whether they thought the results of the test were difficult to understand. Families who received a diagnosis of ADHD were more likely to view the QbTest as useful for understanding how the diagnosis was made, than those who were not diagnosed.(4) Similarly, in the Focus ADHD study, only 10/22 patients/ carers surveyed felt that when the clinician talked through the QbTest results with them, it helped them to understand how they reached the diagnosis. The respondents did not have a strong opinion about whether the results were difficult to understand (votes were split and many voted “neither agree/ disagree”), but some respondents noted in free text responses that they did not find the test helpful because the results were not properly explained to them.(6)

In two studies, parents/ carers provided a more positive view on the QbTest for aiding communication, with the majority of survey respondents reporting that the clinician talking through the results helped them to understand how their diagnosis had been made.(5, 7)

##### Understanding of subjective experience

###### Findings from qualitative data

Clinicians reported in focus groups that the test helped them to better understand the young person’s subjective experience.(7) Additionally, one staff member interviewed as part of the FACT RCT reported that the QbTest helped the young person and the staff to better understand the young person’s behaviours.(1)

*“It feels as if it brings another layer into knowing some of the children” - CAMHS professional on the use of the QbTest(7)*

Clinicians and families interviewed in the qualitative sub-study of the AQUA RCT appreciated that the QbTest provided what they regarded as an objective and observable measure of symptoms.(4) This finding was echoed in focus groups with clinicians in Child and Adolescent Mental Health Services (CAMHS),(7) interviews with healthcare staff in the Focus ADHD study,(6) and by one staff member interviewed in the FACT RCT.(1)

*“I think to be able to see something, it’s that black and whiteness of it, to look at it and go yeah I can see that” - Parent on the use of the QbTest(4)*

###### Findings from survey data

Findings from surveys with healthcare professionals were in line with the interview data in suggesting that the QbTest can help staff to better understand the patient’s symptoms. In the AQUA RCT sub-study, all 10 clinicians surveyed felt that the QbTest had helped them to better understand the patient’s ADHD symptoms.(4) Likewise, most healthcare professionals surveyed in the Focus ADHD study agreed that the QbTest results were helpful in understanding their client’s symptoms,(6) as did clinicians surveyed who had used the QbTest in CAMHS.(7).

Findings were more mixed from surveys with patients and carers. In the AQUA RCT qualitative sub-study, only 35/ 73 families surveyed felt that it helped them to understand their child’s symptoms better.(4) Likewise, only eleven out of 22 patients/carers surveyed in the Focus ADHD study felt that the QbTest helped them to understand their symptoms.(6) In a survey of 10 adolescent boys in a young offenders institute who used the QbTest in the FACT RCT, the majority of respondents reported that they neither agreed nor disagreed that the QbTest helped them to understand their ADHD symptoms or changes in their symptoms.(1) Two studies reported more beneficial effects of the QbTest on level of understanding. In one study, 13/15 children/ adolescents reported that the QbTest helped them to understand their symptoms,(7) and in the other study, 41/48 children (and their families) felt that it helped them to understand their symptoms.(5)

#### Barriers to implementation of the QbTest

Themes we identified regarding views around barriers to the implementation of the QbTest included: practical barriers, other barriers, and acceptability to patients/ carers.

##### Practical barriers

###### Space

Findings from qualitative data

Interview data from three studies highlighted that a room is required to be able to administer the QbTest, and sometimes this is hard to arrange, which means the equipment may need to be moved between rooms.(1, 4, 6) Focus groups with clinicians in CAMHS highlighted concerns about managing environmental factors influencing the QbTest.(7)

*“The main [challenges] were just the practical side, like the room space and things. It’s really competitive to get rooms here so making sure it was booked well in advance.” – Healthcare professional on the use of QbTest(6)*

Findings from survey data

None reported.

###### Staffing

Findings from qualitative data

Clinicians in the AQUA sub-study said that use of the QbTest requires someone trained to administer the task and they thought it is best delivered by healthcare assistant, then interpreted by clinician. However, some healthcare professionals noted that it was important to observe the test to assess the validity of the results.(4) Similarly, in focus groups conducted with clinicians in another study, whilst some clinicians felt that hiring an administrator to administer the test would be helpful, others felt that observing a young person complete the QbTest provided extremely valuable information and this superseded the value that a team would receive from a QbTest administrator.(7) Staff interviewed in the Focus ADHD study highlighted issues with training needs and staff capacity,(6) and interviews with clinicians in one other study flagged the need for continued supervision and learning about the test.(7)

*“If you’re not aware of what’s actually happening at that time, then I think it might be difficult… the actual observation, what’s happening during that time, is very important” – Healthcare professional on the use of QbTest(4)*

Findings from survey data

None reported.

###### Technology

Findings from qualitative data

Some clinicians in the AQUA RCT had issues with technology (internet connection, access to printer) and lack of resources.(4) Likewise, focus groups with clinicians reported being intimidated by the technology and noted instances of QbTest reports disappearing, connectivity issues, and components of the test breaking.(7) Staff in the FACT RCT also reported concerns because of equipment and IT system needed,(1) and staff interviewed in the Focus ADHD similarly flagged issues with equipment and Wi-Fi, including challenges with finding a room with a Wi-Fi connection, accessing laptops and sharing passwords.(6)

*“There was a lot of IT [Information Technology] governance issues to get it set up” - Healthcare professional on the use of QbTest(4)*

Findings from survey data

None reported.

##### Other barriers

###### Findings from qualitative data

Funding was mentioned as a resource need in the Focus ADHD study.(6) Additionally, a lack of follow-up was highlighted in the AQUA sub-study. Some families interviewed felt abandoned by the service after diagnosis and those who received medication reported they should have been more closely monitored. Additionally, those who didn’t receive medication were unclear of what options were available.(4) However, it is not clear how this relates to the QbTest as opposed to the general diagnostic process.

*“Like I just feel like maybe my child by the doctors and stuff has been let down a bit by not being seen and just like he said he should have been seen really after the medication and he hasn’t” -Parent of child who used QbTest(4)*

With regards to treatment management, clinicians highlighted that having more appointments can present scheduling issues and requires the young person to take more time out of school. Therefore, they reported a preference to only add additional QbTest appointments when it was perceived to add value.(11)

###### Findings from survey data

None reported.

##### Acceptability to patients and caregivers

###### Findings from qualitative data

Two studies reported qualitative data concerning the acceptability of the QbTest. In the FACT RCT, some of the adolescent boys interviewed reported that they found the QbTest boring or felt exhausted by it and one person felt cross that they had to repeat the test. However, one person did report that they would recommend the test to others (no quotes provided).(1)

In the Focus ADHD study, interviews with healthcare staff highlighted that particular groups struggled to use the test. Some young people experienced sensory discomfort during the QbTest and some individuals with Autism also struggled with having the tight headband around their head. In some instances, the individual could adapt the test (e.g. to wear a hoodie underneath the headband), however these issues did prevent some individuals from completing the test. Staff also reported that some young people (particularly six year olds) struggled with anxiety during the test, due to the test itself and/or being without their caregivers. Additionally, some of the younger children struggled to follow the instructions and some older teenagers disengaged from the test and became disruptive. Further issues were raised about the language used in the assessment (e.g. use of the word “test” made people stressed), the length and repetitive nature of the test, the lack of representation of different ethnicities in the explanation video, and the requirement to choose biological sex before conducting the test.(6)

*“A lot of our young people that come in for both an autism and an ADHD assessment can experience difficulty with the plastic covering of the headband, because it’s quite a sensory thing on the head and that can be quite uncomfortable. It’s quite tight on the forehead and around the head.” – Healthcare professional on the use of QbTest(6)*

###### Findings from survey data

Four studies provided information about the acceptability of the QbTest from surveys to patients/ carers.(1, 5-7) Findings were mixed between studies, with some participants finding the QbTest difficult to complete, and others not having issues with the test.

In a survey of 10 adolescent boys assessed for ADHD in the FACT RCT (based in a young offenders institution), the majority (9/10) of respondents said that they found the QbTest assessment very stressful and that the task took too long. Additionally, eight out of ten respondents agreed that the task was difficult to complete.(1)

In contrast, in a survey of 48 children (and their families) who had used QbTest in CAMHS, the majority of respondents reported that the results were not difficult to understand and did not find the task difficult to complete.(5) Additionally, in a survey to 15 children/ adolescents who had used the QbTest in a study conducted in CAMHS, 67% did not find the task difficult to complete and most (93%) agreed that overall the experience of using the test was helpful. There was no clear consensus in this study on whether respondents found the stool/chair very uncomfortable or whether the QbTest results were difficult to understand.(7)

In the Focus ADHD study, there was no clear consensus on whether the QbTest was difficult to complete (3/22 said it was, 9 neither agree/ disagree, 10 strongly disagree/ disagree).(6) Although, some of the participants surveyed reported issues with the test, including that their child could not sit through the full test, the QbTest machine did not work in their appointment, and that they felt the staff member delivering the test did not know what they were doing.

Two studies provided information from surveys about the acceptability of the QbTest for clinicians.(5, 7) In a survey of 17 clinicians who had used the QbTest in CAMHS, 13/17 clinicians agreed that the QbTest was easy to use. Additionally, all clinicians agreed that the test helps them to visualise and quantify symptoms, it is a great addition to other investigative techniques, and it is helpful to monitor the effects of treatment and to standardise assessment and treatment.(7) Whereas, in another study that involved a survey to clinicians in CAMHS (n=not reported), 30% of respondents found the results difficult to understand.(5)

**QbCheck:** One study provided survey data on the acceptability of the QbCheck, from a short survey given to 125 patients (56 with ADHD; 69 healthy controls) in a diagnostic test accuracy study.(9) The participants reported that they found the test easy to use, including performing the preparations before starting the test, and understanding and following the test rules during the test. The questions were scored on a scale of 0-10 with higher scores indicating higher ease of use, and mean values were all =>8.06. The most common reason for a score less than 8 was that the test took a long time, so it was hard to stay focused.

### Other quantitative data

**Before-after studies**

| **Study** | **Outcome** | **Details** | **Group 1:** Standard ADHD assessment (pre-implementation) | | **Group 2:** QbTest (6-12) or QbTest (12-60) + Standard ADHD assessment (post-implementation) | | **Effect measure – estimate (95% CI)** | **p-value** | **Other reported details** |
| --- | --- | --- | --- | --- | --- | --- | --- | --- | --- |
|  |  |  | **n** | **No. Events** | **n** | **No. Events** |  |  |  |
| **Hall (2016)(2)** | Number of consultations to ADHD diagnosis | Number of consultations until ADHD diagnosis (mean, min, max) | 40 | Mean 3.05 (min 1, max 7) | 40 | Mean 2.18 (min 1; max 4) | Poisson regression incidence rate ratio (95% CI) 0.71 (0.54, 0.94) | P = 0.02 |  |
|  | Reasons for delay in diagnosis | Clinician-reported reasons for delay in diagnosis, in those where =>5 consultations were needed to make a diagnosis (all in pre-QbTest group) | For 4/6 (66.6%) of cases, inconclusive or discrepancy outcomes from clinical rating scales were cited as the primary reason for delay, one case (17.0%) cited complex comorbidities and one (17.0%) clinician reluctance to make a diagnosis.) | | - | - | - | - |  |
|  | Consultation cost | Total cost spent on ADHD assessment for all 40 cases combined | 40 | £13,176 | 40 | £10,636 | Saving = £2,540 | - | Cost of a consultation within the Trust at the time of audit = £108.00. A single QbTest cost the Trust £31.00 (cost of the test as a proportion of the lease fee, and a 30 min nurse-led appointment to conduct the test). |
| **Vogt (2011)(10)** | Diagnoses revised to ADHD+ in those with a diagnosis rejected at initial assessment at 1-year follow-up | - | 19 | 7 | 19 | 0 | - | p=0.0035 |  |
|  | Outcomes of those with ADHD at 1 year follow-up | ADHD diagnosis changed | 27 | 1 | 43 | 1 | - | p=0.24 |  |
|  |  | Continuing on medication |  | 13 |  | 28 |  |  |  |
|  |  | Discontinued medication |  | 9 |  | 9 |  |  |  |
|  |  | Medication trial |  | 22 |  | 38 |  |  |  |
|  |  | **Lost to follow-up** |  | 3 |  | 4 |  |  |  |
| **Sharma (2022)(8)** | Number of contacts to diagnosis | **All participants** | 20 | Mean 2.7 (SD 0.7) | 20 | Mean 2.4 (SD 0.8) | - | p>0.05 | - |
|  | Number of months to diagnosis | All participants | 20 | Mean 6.5 (SD 3) | 20 | Mean 5.5 (SD 1.8) | - | p>0.05 | - |
|  | ADHD confirmed diagnosis rate | All participants | 20 | 90.6% | 20 | 87.5% | - | p>0.05 | - |
|  | Number of months to diagnosis | ADHD cases (those referred for ADHD) | NR | NR | NR | Mean 5.6 (SD 1.7) | - | - | - |
|  | Number of months to diagnosis | Complex cases (those originally referred for non-specific behavioural difficulties or ASD) | NR | NR | NR | Mean 5.5 (SD 2.7) | - | - | - |
| **Humphreys (2018)(5)** | Number of appointments to diagnostic decision | - | 60-90 | Range of 3-8 appts | 60-90 | Reduction compared to control of between (on average) 0.24 and 1.04 appts per child. In two trusts, a diagnosis was often reached at the first contact with paediatrician. | - | - | - |
|  | Number of days to diagnostic decision | - | 60-90 | Average ranged from 161-453 (approx. 5-15 months) | 60-90 | Average ranged from 15-252 (approx. 2w-8.5 months) | - | - | The authors note for this outcome that the post-implementation group is after introduction of the QbTest AND pathway re-design in two sites. |
|  | Number of days from assessment to commencing medications | - | 60-90 | Range 42-179 days | 60-90 | Range 15-96 days |  |  |  |
|  | Release of clinical time required to reach a diagnostic decision | - |  |  |  | Range 20% to 33% reduction |  |  |  |
| **McKenzie (2022)(6)** | Number of clinical appointments | **All sites** | 549 | Mean 3.22 (range 1-50) | 549 | Mean 2.85 (Range 1-32) | Percent change: 11.5% decrease | NR | Data in this study likely affected by COVID-19 for all Group 2 data and comparison between groups 2 and 1 |
|  | Number of days from initial referral to diagnosis | **All sites** | 549 | Mean 452 (Range 15-3276) | 549 | Mean 507 (Range 43-1281) | Percent change: 12.2% increase | p<0.01 |  |
|  | Number of days to reach diagnostic decision | **All sites** | 549 | Mean 117 (Range 0-1570) | 549 | Mean 129 (Range 0-1378) | Percent change: 10.3% increase | NR |  |
|  | Number of school observations utilised |  | 549 | 120 | 549 | 49 | Percent change: 17% decrease |  |  |
|  | Number of ADHD diagnoses |  | 549 | 445 | 549 | 418 | Percent change: 5% decrease |  |  |
|  | Number of clinical appointments | **CAMHS services** | 326 | Mean 4.13 (Range 1-50) | 326 | Mean 3.75 (Range 1-32) | Percent change: 9.2% decrease |  |  |
|  | Number of days from initial referral to diagnosis | **CAMHS services** | 326 | Mean 442 (Range 18-1161) | 326 | Mean 566 (Range 43-1821) | Percent change: 28.1% increase |  |  |
|  | Number of days to reach diagnostic decision | **CAMHS services** | 326 | Mean 119 (Range 0-888) | 326 | Mean 135 (Range 0-1378) | Percent change: 13.4% increase |  |  |
|  | Number of clinical appointments | **Paediatric clinics** | 194 | Mean 2.01 (Range 1-15) | 194 | Mean 1.63 (Range 1-4) | Percent change: 18.9% decrease |  |  |
|  | Number of days from initial referral to diagnosis | **Paediatric clinics** | 194 | Mean 444 (Range 15-3276) | 194 | Mean 367 (Range 1494) | Percent change: 17.3% decrease |  |  |
|  | Number of days to reach diagnostic decision | **Paediatric clinics** | 194 | Mean 130 (Range 0-1570) | 194 | Mean 138 (Range 0-1036) | Percent change: 6.2% decrease |  |  |

**RCTs**

| **Study** | **Outcome** | **Details** | **Group 1:** QbTest + usual care | | **Group 2** Usual care | | **Effect measure – estimate (95% CI), p value** | **p-value** |
| --- | --- | --- | --- | --- | --- | --- | --- | --- |
|  |  |  | **n** | **No. Events** | **n** | **No. Events** |  |  |
| Chitsabesan (2022)(1)  **Small feasibility trial – interpret with caution** | Time to assessment (*n=20 who completed the QbTest)* | Median no. days between randomisation and QbTest | 20 | Median (IQR) = 42 (26-93). Min=1; max=195 | NR | NR | NR | NR |
|  | Impact on clinical decision making | Diagnostic decision made (all decisions were exclusion of ADHD diagnosis) | 30 | 8 | 30 | 6 | NR | NR |
|  | Morbidity | SDQ baseline: Close to average | 30 | 7 | 30 | 5 | NR | NR |
|  |  | SDQ baseline: Slightly raised | 30 | 4 | 30 | 8 | NR | NR |
|  |  | SDQ baseline: High | 30 | 2 | 30 | 5 | NR | NR |
|  |  | SDQ baseline: Very High | 30 | 16 | 30 | 12 | NR | NR |
|  |  | SDQ baseline: Missing | 30 | 1 | 30 | 0 | NR | NR |
|  |  | SDQ 3m: Close to average | 23 | 2 | 24 | 4 | NR | NR |
|  |  | SDQ 3m: Slightly raised | 23 | 0 | 24 | 5 | NR | NR |
|  |  | SDQ 3m: High | 23 | 4 | 24 | 1 | NR | NR |
|  |  | SDQ 3m: Very High | 23 | 7 | 24 | 7 | NR | NR |
|  |  | SDQ 3m: Missing | 23 | 17 | 24 | 13 | NR | NR |
|  |  | SDQ 6m: Close to average | 9 | 2 | 10 | 3 | NR | NR |
|  |  | SDQ 6m: Slightly raised | 9 | 0 | 10 | 4 | NR | NR |
|  |  | SDQ 6m: High | 9 | 0 | 10 | 1 | NR | NR |
|  |  | SDQ 6m: Very High | 9 | 7 | 10 | 1 | NR | NR |
|  |  | SDQ 6m: Missing | 9 | 21 | 10 | 21 | NR | NR |
| Hollis (2018)(3) | Impact on clinical decision making | Diagnostic decision (confirming or excluding ADHD diagnosis) made | 123 | 94 | 127 | 76 | OR 2.43 (1.34-4.39) | p=0.003 |
|  | Diagnostic status | ADHD confirmed | 123 | 69 | 127 | 65 | RRR = 2.14 (1.00-4.59), | p=0.049 |
|  |  | ADHD excluded | 123 | 25 | 127 | 11 |  |  |
|  |  | No decision made (dropped out or discharged from clinic) | 123 | 29 | 127 | 51 |  |  |
|  | Diagnostic confidence: | Possible/ Uncertain | 122 | 16 | 121 | 29 | OR 1.77 (1.09-2.89), | p=0.022 |
|  |  | Probable | 122 | 32 | 121 | 34 |  |  |
|  |  | Definitely | 122 | 74 | 121 | 58 |  |  |
|  | Stability | Stability in diagnosis (any change in diagnosis from first confirmed diagnosis throughout study) | 123 | Kappa (95% CI) = 1 (1-1) | 127 | Kappa (95% CI) = 0.90 (0.7-1) | (χ2(1)=0.01,) | p=0.32 |
|  | Time to diagnostic decision | Number of minutes spent at clinic appointments until diagnosis | 123 | Mean (SD) = 141.97 (53.84)  Observed median survival time (95% CI) = 150 (140-155) | 127 | Mean (SD) = 152.83 (75.88)  Observed median survival time (95% CI) = 165 (150-180) | Time ratio: 0.85 (0.77-0.93), | p=0.001 |
|  |  | Number of days to diagnostic decision | 123 | Mean (SD): 82.54 (49.53)  Observed median survival time (95% CI) = 96 (85-99) | 127 | Mean (SD): 83.94 (58.14)  Observed median survival time (95% CI)= 108 (91-140) | Time ratio: 0.90 (0.73-1.10), | p=0.285 |
|  |  | Number of clinic appointments until diagnosis | 123 | Mean (SD):  All participants: 2.69 (0.85)  Those with diagnostic decision: 2.82  Those who dropped out or were discharged without a diagnosis: 2.28 | 127 | Mean (SD):  All participants: 2.72 (0.91)  Those with diagnostic decision: 2.76  Those who dropped out or were discharged without a diagnosis: 2.67 | NR |  |
|  |  | No. consultations to diagnostic decision (confirming or excluding ADHD diagnosis) by group over six-months, n=250 | 123 | - | 127 | - | HR 1.44 (1.04-2.01) | p=0.029 |
|  |  | No. consultations to diagnostic decision (confirming or excluding ADHD diagnosis) by group over six-months, in n=198 aged 6-12 years (**using QbTest in intervention group)** | NR | - | NR | - | HR 1.84 (1.23 to 2.68), | p=0.001 |
|  |  | No. consultations to diagnostic decision (confirming or excluding ADHD diagnosis) by group over six-months, in n=52 aged >12 years (**using QbTest(12-60) in intervention group)** | NR | - | NR | - | HR 0.82 (0.37-1.80), | p = 0.618 |
|  | Costs | Cost of clinic appointments | 123 | Mean (SD): £87.62 (£40.45) | 127 | Mean (SD): £90.06 (£41.19) | - |  |
| Williams (2021)(11)  **Small feasibility trial – interpret with caution** | Use of interventions e.g. ADHD medication | Change to type or dose of ADHD medication at follow-up 1 (2-4 weeks) | 18 | 10 | 21 | 7 | NR | NR |
|  |  | Change to type or dose of ADHD medication at follow-up 2 (8-12 weeks) | 17 | 7 | 19 | 9 | NR | NR |
|  |  | Medication adherence at follow-up 1: taken medication most/every day | 8 | 8 | 9 | 8 | NR | NR |
|  |  | Medication adherence at follow-up 2: taken medication most/every day | 8 | 7 | 9 | 9 | NR | NR |

# References

1. Prathiba Chitsabesan, Charlotte Lucy Hall, Lesley-Anne Carter, Mindy Reeves, Vaseem Mohammed, Bryony Beresford, et al. Using an objective computer task (QbTest) to aid the identification of attention deficit hyperactivity disorder (ADHD) in the Children and Young People Secure Estate (CYPSE): a feasibility randomised controlled trial. BMJ Open. 2022;12(12):e064951.

2. Hall Charlotte L, Selby Kim, Guo Boliang, Valentine Althea Z, Walker Gemma M, Hollis Chris. Innovations in Practice: an objective measure of attention, impulsivity and activity reduces time to confirm attention deficit/hyperactivity disorder diagnosis in children - a completed audit cycle. Child and adolescent mental health. 2016;21(3):175-8.

3. Hollis C, Hall CL, Guo B, James M, Boadu J, Groom MJ, et al. The impact of a computerised test of attention and activity (QbTest) on diagnostic decision-making in children and young people with suspected attention deficit hyperactivity disorder: single-blind randomised controlled trial. Journal of child psychology and psychiatry, and allied disciplines. 2018;59(12):1298-308.

4. Charlotte L. Hall, Althea Z. Valentine, Gemma M. Walker, Harriet M. Ball, Heather Cogger, David Daley, et al. Study of user experience of an objective test (QbTest) to aid ADHD assessment and medication management: a multi-methods approach. BMC psychiatry. 2017;17(1):66.

5. Catriona Humphreys, Lucy Sitton-Kent. Transforming ADHD Care Across the East Midlands: An evaluation Report. East Midlands Academic Health Network 2018 [Available from: <https://healthinnovation-em.org.uk/component/rsfiles/download-file/files?path=our-work%252Four-innovations%252FTransforming-ADHD-Care%252FFinal_Overall_Evaluation_Report_31May18.pdf&Itemid=1457>

6. Hall SS, McKenzie C, Thomson L, Ingall BR, Groom MJ, McGlennon N, Dines-Allen M, Hall CL. A national evaluation of QbTest to support ADHD assessment: a real-world, mixed methods approach. BMC Health Services Research. 2024 Oct 8;24(1):1201.

7. Pellegrini Seán, Murphy Mike, Lovett E. The QbTest for ADHD assessment: Impact and implementation in Child and Adolescent Mental Health Services. Children & Youth Services Review. 2020;114:n.r.

8. Sharma RW, A. Lacey, S. Spiewakowski, D. IMPLEMENTING QB TESTING FOR ADHD: EVALUATING VALUE IN A DGH SETTING. Archives of Disease in Childhood. 2022;107(Supplement 2):A70.

9. Ulberstad FB, Hans Chavanon, Mira-Lynn Knollmann, Martin Wiley, James Christiansen, Hanna Thorell, Lisa B. Objective measurement of attention deficit hyperactivity disorder symptoms outside the clinic using the QbCheck: Reliability and validity. International journal of methods in psychiatric research. 2020;29(2):e1822.

10. Vogt C, Shameli A. Assessments for attention-deficit hyperactivity disorder: Use of objective measurements. Psychiatrist. 2011;35(10):380-3.

11. Williams L, Hall C. L., Brown S, Guo B, James M, Franceschini M, et al. Optimising medication management in children and young people with ADHD using a computerised test (QbTest): a feasibility randomised controlled trial. Pilot and feasibility studies. 2021;7(1):68.

12. 2014-001488-11. Effects of expectations, medication and placebo during the Quantified Behavior Test in patients with untreated ADHD and Substance Use Disorder 2014 [cited &lt;br&gt;Female: yes&lt;br&gt;Male: yes&lt;br&gt; Interventional clinical trial of medicinal product Controlled: yes Randomised: yes Open: no Single blind: no Double blind: yes Parallel group: no Cross over: yes Other: no If controlled, specify comparator, Other Medicinial Product: no Placebo: yes Other: no Number of treatment arms in the trial: 2]. Available from: <https://www.clinicaltrialsregister.eu/ctr-search/search?query=eudract_number:2014-001488-11>.
