# Supplementary material for: QbTest for ADHD assessment and medication management: a mixed-methods systematic review of impact on clinical outcomes and patient, carer and clinician experiences
Source: BMJ Open. 2025 Apr 17;15(4):e095479. doi: 10.1136/bmjopen-2024-095479 (PMC12007030; doi:10.1136/bmjopen-2024-095479)
Supplement: Supplementary file 2 [file bmjopen-2024-095479.reviewer_comments.pdf]

PEER REVIEW HISTORY

BMJ Open publishes all reviews undertaken for accepted manuscripts. Reviewers are asked to complete a checklist review form and are provided with free text boxes to elaborate on their assessment. These free text comments are reproduced below.

ARTICLE DETAILS

Title (Provisional)

QbTest for ADHD assessment and medication management: a mixed-methods systematic review of impact on clinical outcomes and patient, carer, and clinician experiences

Authors

Tomlinson, Eve; Owen-Smith, Amanda; Benavente, Melissa; Cooper, Chris; Jones, Hayley E.; Ward, Mary; Walker, Josephine; Wang, Hanyu; Lopez Manzano, Catalina; Hank, Dietmar; Welton, Nicky; Leeflang, Mariska; Whiting, Penny

---

VERSION 1 - REVIEW

**Reviewer**	**1**
**Name**	**Hall, Charlotte**
**Affiliation**	**Institute of Mental Health, NIHR CLAHRC-East Midlands, Psychiatry**
**Date**	**29-Dec-2024**
**COI**	**None**

This paper is a systematic review of user outcomes and experiences when using the QbTest to support the assessment of ADHD. This paper presents quantitative and qualitative data on the QbTest.

Overall, the paper is well written. However, I was a little unclear what this systematic review adds to an already extensive amount of reviews on QbTest and would like the authors to address this more clearly, which for me, would also require acknowledging the existing reviews in this field and how this is different.

My specific comments are:

Title: The title could do with improving. It reads like the literature will only look at QbTest for ADHD assessment, but actually it looks at medication management too. Also could the title be modified to make it more clear who the users are so readers are more clear on the aim of the article.

ABSTRACT:

The abstract would benefit from one line stating what QbTest is and QbCheck – I appreciate word limits can make this detail difficult. The abstract mentions QbMini and QbMT without any explanation what these are and how they differ to QbTest and QbCheck – these are far well less known even to readers who may be familiar with QbTest.

Not all acronyms are spelt out in the abstract – including RCT

Study selection needs modifying to make it clear you are also including QbCheck

INTRODUCTION:

- Line 5 – ADHD is not always prevalent throughout the lifespan – although it can be – reword.

- The second paragraph reads very similar to previously published papers on QbTest – could the authors make this more novel? I had the impression I'd read this introduction many times before

- The introduction would also benefit on a reflection of adults vs children/young people as right now the two aren't really differentiated, but for example, asking for teacher reports for adults is rarely feasible/appropriate. And the same for the reference to educational needs

- Given you refer to multiple versions of QbTest, these need to be explained.

- The introduction does not refer to any previous systematic reviews on QbTest such as : https://acamh.onlinelibrary.wiley.com/doi/full/10.1111/jcpp.13901 and https://pmc.ncbi.nlm.nih.gov/articles/PMC10758132/ and https://www.magonlinelibrary.com/doi/full/10.12968/jprp.2024.0004?casa_token=0P1QMkGPVCMAAAAA%3APb5vt9FRyo9-WbY3Bv-jxPTKqkCwCiPRcI2Hqb_7xJa126iCQUpUH82YSbRPT7-0aVB-7a6fUQI and this paper although not a systematic review: https://www.sciencedirect.com/science/article/abs/pii/S1751722223001051?casa_token=TOZqHjGx_I4AAAAA:VwBMp75LlOJ3AteZAakns1SWAR_ToBSS0hfS9Ctdv0J7CdWViz2Xtn94QL7W-VAaWuYWnis

I think my biggest issue with this review is that I feel its not adding much to these existing reviews, some of which are very recent. I feel the authors need to make this much more clear.

AIM:

- The aim feels like it needs more clarity when you say patient outcome and also what this review will achieve over other existing ones.

METHIOD:

- You don't say clearly what you exclude – so for example there are other review papers on QbTest and perspective/opinion papers – were these eligible to be included, i think not, but that doesn't feel clear to me in the methods.

- I also struggled to know which outcomes were included, so for example, a paper that showed a good dose response (e.g. reduction in symptoms after taking MPH) was not included but that is a patient outcome?

- Qbtech does not have a capital T and should be referred to as Qbtech Ltd. – a link to their website would be useful.

- I believe the McKenzie report you refer to is this paper: https://link.springer.com/article/10.1186/s12913-024-11693-7 (different reference)

- I am not sure why you would include one study with no reported results – it doesn't feel to fit with your aim? Or did i misunderstand?

RESULTS AND DISCUSSION

- As above, I think the McKenzie paper may be superseded now

- Also the reference for Hollis for the qual paper is incorrect in your table (ref 38 – is Hall et al)

- I wonder if the RoB assessment didn't account for if the statistical analysis used accounted for missing data? which led to most studies being high RoB? Additionally, sometimes this data didn't appear to be available because the participants were simply not getting a diagnosis within the study time frame, given that (as you state) ADHD diagnosis can be lengthy, it would involve an extensive follow-up (for many many years) - you should refer to the practicality of this.

- You don't say what the important confounders were part from one study where you mention COVID-19.

- I think its worth reflecting if the study authors mention their confounds/limitations in their conclusion or interpretation of results. If they don't then the study would be of high RoB, if this is reflected on in data interpretation – should this really be the same?

- I think it would be interesting to suggest how these RoB could be overcome in future study designs.

- Given NICE have approved QbTest in children and not adults, I feel the results should be structured into these categories within the existing subgroups.

- The review should better acknowledge limitations of the approach taken over and above the lack of RCTs found (although i agree with this is a limitation).

REFERENCING

- formatting of references is inconsistent throughout the reference list

TABLES

- Clear and well presented

**Reviewer**	**2**
**Name**	**Sharma, Ramona**
**Affiliation**	**The University of British Columbia Okanagan, School of Social Work**
**Date**	**27-Jan-2025**
**COI**	**None**

This paper explores patient, carer, and clinician experiences of the QbTest and its impact on ADHD diagnosis and medication management, focusing on outcomes such as time to diagnosis, clinical decision-making, and test acceptability. Findings suggest that the QbTest is generally well-received, can reduce time to diagnosis, improve clinician confidence, and lower costs, though barriers such as staffing, technology, and test length persist, with limited data on its role in medication management.

This manuscript has several strengths, the most notable of which are patient and public involvement, specifically involving ADHD individuals as this is rarely done, as well as pre-registered methods. The manuscript's focus on ADHD diagnosis and medication management using the QbTest is also timely and relevant, especially given increasing reliance on digital tools in clinical settings. The mixed-methods approach is a notable strength, as it integrates quantitative and qualitative data to provide a holistic perspective. Additionally, the clear acknowledgment of gaps in evidence for adult populations and medication management shows a thoughtful approach to framing the research.

However, there are several grey areas that need to be addressed before this paper can be considered for publication. My comments are below, section by section:

Title and abstract:

The title effectively conveys the study's focus on the QbTest as a diagnostic tool for ADHD and highlights the mixed-methods approach. The abstract is well-structured, succinctly outlining the objectives, methodology, results, and conclusions.

However, the title could be made more precise by explicitly mentioning the population focus and methodology. For example, including "children and adolescents" in the title would improve clarity. Additionally, the abstract lacks specific quantitative findings, which would make the results more impactful and informative.

Refine the title to include the demographic focus, e.g., "QbTest for ADHD assessment in children and adolescents: A mixed methods systematic review of clinical and user outcomes."

If possible, in the abstract, include specific numbers to quantify the key findings, such as "reduced diagnostic time by X-Y days" or "improved clinician confidence by X-Y%."

Scope and objectives:

The scope could be more explicitly framed in the introduction. While the manuscript addresses gaps in research, the objectives section would benefit from directly emphasizing these gaps and making the economic implications of the QbTest a more prominent part of the discussion.

Add a sentence in the objectives section highlighting that the study focuses on children and adolescents, with limited data on adults. Additionally, emphasize that future research should explore cost-effectiveness to guide healthcare decision-making.

Methods:

The methodology is robust, adhering to PRISMA-2020 guidelines and registering the protocol in PROSPERO. The comprehensive search strategy across multiple databases demonstrates a commitment to thoroughness. The mixed-methods synthesis using the convergent-integrated approach is a key strength, allowing for nuanced insights.

However, some aspects of the methodology could be clarified. The reliance on single-reviewer data extraction, even with cross-checking, could introduce bias, and this should be acknowledged and addressed. Furthermore, the exclusion of other continuous performance tests (CPTs) like Nesplora or EFSim warrants a more detailed justification. Provide a more detailed explanation for focusing on QbTest over other CPTs, such as its research prominence or NICE recommendation.

"The searches were drawn from our broader Diagnostic Assessment Report

conducted for NICE" - Expand upon this and very briefly situate NICE for readers who are unfamiliar.

"All data items are provided in supplementary material" - Refer to which appendix or file specifically.

"Qualitative data (we excluded question 10 "how valuable is the research?")" - Very briefly explain the rationale behind this.

"There were not sufficient data reported to stratify by subgroups." - Why? What was your criterion? Be more specific with your justification, e.g., provide the n's of subgroups, etc.

Results:

The results section is comprehensive and integrates quantitative and qualitative findings effectively, providing a clear picture of the QbTest's impact on diagnostic processes and user experiences. A notable strength is the exploration of barriers to implementation, such as technological and staffing challenges, which adds practical value to the findings.

However, some results lack quantitative specificity, which reduces their impact. For example, while the reduction in diagnostic time is mentioned, exact figures are absent. Additionally,

subgroup analyses by sex, ethnicity, or comorbidities are not provided, limiting the generalizability of the findings.

If possible, include numerical data for key outcomes, such as the average reduction in diagnostic time (e.g., "reduced by X days") and improvements in clinician confidence (e.g., "increased by Y%"). Acknowledge the lack of subgroup analyses and its implications for understanding how findings might vary across demographics.

“Most studies reported both quantitative and qualitative data” and other similar statements - provide the number or percentage. That said, for # of studies endorsing a finding (e.g., Three studies were RCTs…), would love to see the percentage too.

“We do not include quantitative patient outcome data from the FACT and QUOTA feasibility RCT studies, because, as noted, they were not designed or powered to evaluate impact on outcomes.” - Provide a citation behind the methodological rationale for this.

Headings/subheadings within Results are confusing, particularly “Findings” - maybe change that one to “Thematic Findings”. I would also move Quality Assessment to the end of results; it is more intuitive to read Characteristics of Articles, than Thematic Findings.

Regarding Quality Assessment - provide a brief summary sentence before saying “Quality assessments are provided in Appendix 5”, as this sentence appears dropped. Clarity in this section is mportant because your findings depend on many high-risk-of-bias studies.

Regarding the thematic findings, the language is a little precarious. I think what you are calling “themes” (i.e., views on the test; barriers) are actually the conceptual categories rather than the themes. One can certainly expect that there will be views on the test and barriers - these don’t really thematically explain what the findings are telling us. However, what you are referring to as conceptual categories are in fact the themes, i.e., contribution to ADHD diagnosis, which then broadly fall into the categories of “views” and “barriers”. Please fix this in the Abstract too.

Once again with the headings - I am finding it very difficult to at a glance figure out what the thematic findings are. For example, under “views around helpfulness of the test”, I feel like the “Contribution to ADHD diagnosis” heading is quite broad and vague. I would suggest using subheadings (subthemes) for themes, e.g., for “Contribution to ADHD diagnosis”, you could have the subthemes “Time Until Diagnostic Decision”, “Proportion of People with Diagnostic Decision”, “Clinician Confidence”, etc. In this fashion, I would revise all the existing headings. You did this for the “Barriers” category, so make it consistent.

Define terms like “Contribution to ADHD diagnosis” and “treatment decision making” when you first mention them - what do these concepts entail? A way to do this might be to provide a topic sentence mapping out the contents of the paragraph so the reader knows what to expect at the beginning of each theme, e.g., “we found XYZ regarding contributions to ADHD diagnosis, i.e., time until diagnostic decision, proportion of people with diagnostic decision….” Etc.

To make everything abundantly clear, I would actually recommend providing a table with the columns “category”, “theme”, and “subtheme”, along with the frequency/percentage of endorsement by articles. An example of such a table is Table 2 here:

Sharma RH, Rodberg D, Struik LL Experiences of nicotine users motivated to quit during the COVID-19 pandemic: a secondary qualitative analysis BMJ Open 2023;13:e070906. doi: 10.1136/bmjopen-2022-070906.

This is just my own work off the top of my head, but don't feel limited by this - there are other examples in the literature too. You might have to play around with the format as this is a review and not an empirical study, but I believe such a table would make the findings clearer and more translatable.

Table 2 with quotes is kind of dropped haphazardly. Discuss the quotes/experiences a bit more in the writing, or build the quotes into the narrative. That said, the whole theme of Barriers it not discussed nearly as much as the first theme. I would appreciate a little more discussion around it, if the word limit allows. See the next comment for additional implications on this.

Overall, a broader discussion of patient experiences is needed, as ADHD is historically diagnosed, assessed, and treated to primarily alleviate how the ADHD individual impacts third parties (parents, teachers) rather than address how the ADHD individual feels themselves. Therefore, if you are making a statement like “well received by users” (Abstract), this needs to be backed up with a broader discussion of how the population in question experiences it too, which may be doubly challenging as the population you are focusing on in this paper is children/youth. The only quote you provide on “Acceptability to patients and caregivers” isn’t a patient or caregiver quote at all - provide patient quotes if possible. If you don’t have this data, you can’t make that statement; consider revising to “well-received by clinicians using it”. Consider that throughout the paper, especially since the only thing differentiating your paper from other quantitative reviews on this topic is the mixed-methods nature of it, i.e., inclusion of qualitative experiences.

Discussion:

Use of subheadings e.g., summary of findings, strengths and limitations, future directions, conclusions, etc., could strengthen the discussion.

“The implementation of the QbTest may further impact upon the availability of ADHD medication by exposing unmet need for treatment.” - I agree with what you said prior to this, that medication is not the only line of treatment. What other treatment modalities can the QbTest support? Be specific so that readers understand that ADHD is treated holistically - most papers unilaterally focus on medications which has created a skewed understanding of ADHD among health practitioners, ADHD individuals, and the general public.

I would caution against endorsing the QbTest too much as you do have quite mixed findings with very high-risk-of-bias studies. Language that is tentative and specific, such as “may

improve the ADHD diagnostic process, when used alongside clinical assessment in children and young people" is appropriate. Ensure you don't extend beyond this.

Is there any literature synthesizing evidence (e.g., review) that ultimately did not endorse the QbTest? It would be interesting to compare findings and would allow for a more nuanced discussion.

I would include a "future directions for practice" paragraph where you discuss possible strategies to remediate some of the practical limitations of the QbTest that you outlined, i.e., barriers.

Overall, this paper is well-written and has potential once the above are addressed!

VERSION 1 - AUTHOR RESPONSE

Reviewer 1

Comments to the author

1. **This paper is a systematic review of user outcomes and experiences when using the QbTest to support the assessment of ADHD. This paper presents quantitative and qualitative data on the QbTest. Overall, the paper is well written. However, I was a little unclear what this systematic review adds to an already extensive amount of reviews on QbTest and would like the authors to address this more clearly, which for me, would also require acknowledging the existing reviews in this field and how this is different.**

Thank you for your feedback. We have amended our introduction to more clearly acknowledge the existing systematic reviews in this field and to outline what our review adds to the literature. Previous systematic reviews have summarised quantitative data, primarily concerning the accuracy of the QbTest. Our review is the first systematic review to synthesise qualitative as well as quantitative data concerning the QbTest and it improves on previous reviews in this area by involving individuals with lived experience of ADHD in the review team.

Title

2. **The title could do with improving. It reads like the literature will only look at QbTest for ADHD assessment, but actually it looks at medication management too. Also could the title be modified to make it more clear who the users are so readers are more clear on the aim of the article.**

Thank you for highlighting this. In line with your suggestion, we have amended the title to: "QbTest for ADHD diagnosis and medication management: a mixed-methods systematic review of impact on clinical outcomes and patient, carer, and clinician experiences".

Abstract

3. **The abstract would benefit from one line stating what QbTest is and QbCheck – I appreciate word limits can make this detail difficult. The abstract mentions QbMini and QbMT without any explanation what these are and how they differ to QbTest and QbCheck – these are far well less known even to readers who may be familiar with QbTest.**

Thank you. We have added more detail to the abstract about the different versions of the QbTest, in the 'study selection' section: "Studies, of any design, that evaluated any version of the QbTest **(QbMini <6 years, QbTest 6-12 or 12-60 years, QbCheck for remote assessment via webcam, or QbMT smartphone version)**, for ADHD diagnosis and/or medication management…".

4. Not all acronyms are spelt out in the abstract – including RCT

Thank you for spotting this – we have now spelled out the acronym "RCT". For ease of identification and readability (word count), we will continue to use the common brand names for the databases.

5. Study selection needs modifying to make it clear you are also including QbCheck

Thank you. We have done this – please see our reply to your comment no3.

Introduction

6. Line 5 – ADHD is not always prevalent throughout the lifespan – although it can be – reword.

We have amended this sentence to say "Attention deficit hyperactivity disorder (ADHD) is a common neurodevelopmental condition that can be prevalent throughout the lifespan".

7. The second paragraph reads very similar to previously published papers on QbTest – could the authors make this more novel? I had the impression I'd read this introduction many times before

Thank you. It is challenging to write this in a different way as it is describing the referral/ diagnostic process, but we have tried to do this, as follows:

"Individuals with suspected ADHD will often be referred by a primary care professional to a specialist in secondary care for further assessment. Referral pathways vary, but for children, referral will often come from a General Practitioner (GP) or education professional to a paediatrician or child and adolescent mental health services. For adults, referral will commonly come from a GP or psychiatric service to a mental health specialist. However, long waiting lists mean that it can be years before ADHD assessment takes place.(5, 6) ADHD diagnosis typically involves a clinician gathering information from multiple sources, including behavioural observations, patient history, interviews, questionnaires, and subjective reports from informants, such as caregivers and teachers for children and young people, or partners/ spouses and family members for adults.(1) The diagnostic process can be challenging and time-consuming for a number of reasons. Informants often provide contradictory perspectives which can cause uncertainty. Additionally, ADHD often co-occurs with other conditions, such as autism spectrum disorders, learning disabilities, and mood disorders, which can exhibit similar symptoms to ADHD, making it difficult to distinguish between conditions.(1) The predominant ADHD symptoms also vary between individuals, which can lead to further inconsistency in diagnosis.(7)

Following ADHD diagnosis, management of ADHD often requires a holistic approach that addresses psychological, behavioural, and occupational (for adults) or educational (for children/ young people) needs. If medication is initiated, careful dose titration and medication management is required to achieve optimal treatment whilst limiting side effects.(8) This also relies on the clinician gathering subjective reports from informants about whether the treatment is working."

8. The introduction would also benefit on a reflection of adults vs children/young people as right now the two aren't really differentiated, but for example, asking for teacher reports for adults is rarely feasible/appropriate. And the same for the reference to educational needs

Thank you for your comment. We have amended the introduction to distinguish more between children/ young people and adults – please see the amended text in our response to your comment no7.

9. **Given you refer to multiple versions of QbTest, these need to be explained.**

Thank you. We have now explained the QbTest versions in the introduction: "Multiple versions of the QbTest exist(13): QbMini (for children aged <5), QbTest 6-12 (for children aged 6-12 years), QbTest 12-60 (for people aged 12-60 years), QbCheck (remote version conducted via webcam), and QbMT (smartphone version in development)."

10. **The introduction does not refer to any previous systematic reviews on QbTest such as : https://acamh.onlinelibrary.wiley.com/doi/full/10.1111/jcpp.13901 and https://pmc.ncbi.nlm.nih.gov/articles/PMC10758132/ and https://www.magonlinelibrary.com/doi/full/10.12968/jprp.2024.0004?casa_token=0P1QMkGPVCMAAAAA%3APb5vt9FRyo9-WbY3Bv-jxPTKqkCwCiPRcI2Hqb_7xJa126iCQUpUH82YSbRPT7-0aVB-7a6fUQI and this paper although not a systematic review: https://www.sciencedirect.com/science/article/abs/pii/S1751722223001051?casa_token=TOZqHjGx_I4AAAAA:VwBMp75LlOJ3AteZAakns1SWAR_ToBSS0hfS9Ctdv0J7CdWViz2Xtn94QL7W-VAaWuYWnis. I think my biggest issue with this review is that I feel its not adding much to these existing reviews, some of which are very recent. I feel the authors need to make this much more clear.**

Thank you for your comment. We have added a paragraph to the introduction in which we refer to previous systematic reviews on the QbTest and outline what our review adds to the literature. This reads as follows: "Previous systematic reviews of the QbTest have suggested that, when used in support of clinical assessment, the test may improve the efficiency of the ADHD diagnostic process(12, 13) and may aid in treatment monitoring.(14) These reviews have summarised quantitative data only and primarily focus on the accuracy of the QbTest. The present mixed-methods review aims to explore patient, carer, and clinician experiences of the QbTest and its impact on patient outcomes for ADHD diagnosis and medication management. This is the first systematic review to integrate qualitative and quantitative data concerning the QbTest and it improves on previous reviews in this area by involving individuals with lived experience of ADHD in the review team."

Aim

11. **The aim feels like it needs more clarity when you say patient outcome and also what this review will achieve over other existing ones.**

Thank you. As noted in our response to your comment no10, we have now made clear in the introduction what this review will achieve over existing ones. Patient outcomes can be challenging to define concisely within the aim, but these outcomes are listed within the eligibility criteria section of the manuscript.

Method

12. **You don't say clearly what you exclude – so for example there are other review papers on QbTest and perspective/opinion papers – were these eligible to be included, i think not, but that doesn't feel clear to me in the methods.**

We included primary studies in this review. We also included conference abstracts. We have made this clearer in the "eligibility criteria" section of the methods. We have phrased our eligibility criteria so that any study that meets these criteria was eligible for inclusion. We do not feel it necessary to specify the exclusion criteria as these will be the opposite to what is stated in this section.

13. **I also struggled to know which outcomes were included, so for example, a paper that showed a good dose response (e.g. reduction in symptoms after taking MPH) was not included but that is a patient outcome?**

We outline which outcomes were included in the review in the “eligibility criteria” section of the methods, as follows: “We included studies of any design that provided quantitative or qualitative data on any of the following outcomes: time to assessment/diagnostic decision, use of services, impact on clinical decision-making, healthcare professionals confidence in assessment, use of interventions, morbidity, mortality, health related quality of life, cost, ease of use, acceptability to patients, clinicians and clinicians, and patient, carer and clinician experience of the test.” We included all studies that we identified that met our eligibility criteria.

14. Qbtech does not have a capital T and should be referred to as Qbtech Ltd. – a link to their website would be useful.

Thank you for flagging this. We have amended this and added a reference to the Qbtech website.

15. I believe the McKenzie report you refer to is this paper: https://link.springer.com/article/10.1186/s12913-024-11693-7 (different reference)

Thank you for highlighting this report, which was published after the date that we conducted our searches. We have added this report to our review and updated the McKenzie citation to refer to this report.

16. I am not sure why you would include one study with no reported results – it doesn't feel to fit with your aim? Or did i misunderstand?

We identified one ongoing study which aims to evaluate the feasibility of the QbMT (smartphone version of the QbTest). There was limited detail available for this study and no reported results, but the aim of the study appeared relevant to this review, so we included it for completeness. This is in line with guidance on systematic review conduct, which states that information about relevant ongoing studies should be included in the review.[1]

[1] Lefebvre C, Glanville J, Briscoe S, Featherstone R, Littlewood A, Metzendorf M-I, Noel-Storr A, Paynter R, Rader T, Thomas J, Wieland LS. Chapter 4: Searching for and selecting studies [last updated September 2024]. In: Higgins JPT, Thomas J, Chandler J, Cumpston M, Li T, Page MJ, Welch VA (editors). *Cochrane Handbook for Systematic Reviews of Interventions* version 6.5. Cochrane, 2024.

Results/ discussion

17. As above, I think the McKenzie paper may be superseded now

Thank you - please see our reply to your comment no15.

18. Also the reference for Hollis for the qual paper is incorrect in your table (ref 38 – is Hall et al)

We include two citations next to “Hollis (2018)” in this table (listed below). This is because one report presents the quantitative results of the AQUA trial (Hollis, 2018) and the other reports on qualitative data from the AQUA trial (Hall, 2017). We have used “Hollis (2018)” as the overall identifier here as we had to select one for the AQUA trial in this table, but throughout the review we cite these papers individually, where relevant.

- Hollis C, Hall CL, Guo B, James M, Boadu J, Groom MJ, et al. The impact of a computerised test of attention and activity (QbTest) on diagnostic decision-making in children and young people with suspected attention deficit hyperactivity disorder: single-blind randomised controlled trial. Journal of child psychology and psychiatry, and allied disciplines. 2018;59(12):1298-308.
- Charlotte L. Hall, Althea Z. Valentine, Gemma M. Walker, Harriet M. Ball, Heather Cogger, David Daley, et al. Study of user experience of an objective test (QbTest) to aid ADHD assessment and medication management: a multi-methods approach. BMC psychiatry. 2017;17(1):66.

19. I wonder if the RoB assessment didn't account for if the statistical analysis used accounted for missing data? which led to most studies being high RoB? Additionally, sometimes this data

didn’t appear to be available because the participants were simply not getting a diagnosis within the study time frame, given that (as you state) ADHD diagnosis can be lengthy, it would involve an extensive follow-up (for many many years) - you should refer to the practicality of this.

Thank you. We agree that if a study had appropriately accounted for missing data within the statistical analysis, then this would lead to less concern for missing data regarding the risk of bias. However, this was not the case in the studies included in this review. Additionally, regarding your point about practicality, in a risk of bias assessment we must judge the risk of bias in the study, not whether the study authors have done the best they can do in the situation. If there are missing data in a study, then whatever the reasons are for this, there is still a potential for bias. This is particularly problematic when the patients with missing data are different to those without missing data. This would be the case with lengthy ADHD diagnosis – as those without a diagnosis are likely to be patients in whom diagnosis was more challenging.

20. **You don’t say what the important confounders were part from one study where you mention COVID-19.**

Thank you for identifying this. We now state the confounders in the quality assessment section of the methods. We also now state these in the quality assessment section of the results: “All five implementation studies were judged at serious risk of bias. In four of these, this was because important confounders (i.e. age at the point of seeking ADHD referral, sex, comorbidities, nature and severity of symptoms at presentation, socioeconomic status, and ethnicity) were not controlled for (Appendix 5).(35, 36, 39, 40) This included the Focus ADHD study, for which an additional important potential confounder was the COVID-19 pandemic.”

21. **I think its worth reflecting if the study authors mention their confounds/limitations in their conclusion or interpretation of results. If they don't then the study would be of high RoB, if this is reflected on in data interpretation – should this really be the same?**

Thank you for your comment. We are assessing bias in an estimate, not bias in the conclusion. If confounding has not been appropriately controlled for in a study, either by design or through using a statistical analysis that adjusts for the confounding factor(s), then there is a potential for confounding of the effect of the intervention, and this will lead to high risk of bias. Whether an author has reflected on their confounders/ limitations in their conclusion or interpretation of results is not relevant to the risk of bias assessment.

22. **I think it would be interesting to suggest how these RoB could be overcome in future study designs.**

The only way to overcome confounding in future study designs is to use a randomized controlled trial (RCT). We emphasise the need for well-designed RCTs in the “suggestions for future research” section of the discussion.

23. **Given NICE have approved QbTest in children and not adults, I feel the results should be structured into these categories within the existing subgroups.**

Thank you for your comment. We have decided not to restructure the results into sections for children and adults, because, as we state in the results, only one study (the QbCheck study) enrolled adults. All other studies evaluated the QbTest (6-12 or 12-60) in children and adolescents.

24. **The review should better acknowledge limitations of the approach taken over and above the lack of RCTs found (although i agree with this is a limitation).**

We don’t feel there are any further limitations to our approach, so we have focused on the limitations to the evidence base.

Referencing

25. Formatting of references is inconsistent throughout the reference list.

Thank you. We have amended the formatting of the references.

Tables

26. Clear and well presented.

Thank you.

Reviewer 2

Comments to the author

1. **This paper explores patient, carer, and clinician experiences of the QbTest and its impact on ADHD diagnosis and medication management, focusing on outcomes such as time to diagnosis, clinical decision-making, and test acceptability. Findings suggest that the QbTest is generally well-received, can reduce time to diagnosis, improve clinician confidence, and lower costs, though barriers such as staffing, technology, and test length persist, with limited data on its role in medication management. This manuscript has several strengths, the most notable of which are patient and public involvement, specifically involving ADHD individuals as this is rarely done, as well as pre-registered methods. The manuscript's focus on ADHD diagnosis and medication management using the QbTest is also timely and relevant, especially given increasing reliance on digital tools in clinical settings. The mixed-methods approach is a notable strength, as it integrates quantitative and qualitative data to provide a holistic perspective. Additionally, the clear acknowledgment of gaps in evidence for adult populations and medication management shows a thoughtful approach to framing the research. However, there are several grey areas that need to be addressed before this paper can be considered for publication. My comments are below, section by section.**

Thank you for your helpful feedback. We have addressed your comments, in turn, below.

Title

2. **The title effectively conveys the study's focus on the QbTest as a diagnostic tool for ADHD and highlights the mixed-methods approach. However, the title could be made more precise by explicitly mentioning the population focus and methodology. For example, including "children and adolescents" in the title would improve clarity. Refine the title to include the demographic focus, e.g., "QbTest for ADHD assessment in children and adolescents: A mixed methods systematic review of clinical and user outcomes.**

Thank you. We have not included "children and adolescents" in the title because the eligibility criteria for our review specified that we would include studies in people of any age. Although only one of the included studies enrolled adults, this is a finding of the review.

Abstract

3. **The abstract is well-structured, succinctly outlining the objectives, methodology, results, and conclusions. The abstract lacks specific quantitative findings, which would make the results more impactful and informative. If possible, in the abstract, include specific numbers to quantify the key findings, such as "reduced diagnostic time by X-Y days" or "improved clinician confidence by X-Y%."**

Thank you for your comment. We do not present quantitative data in the abstract because we followed the convergent-integrated approach to mixed-methods synthesis[1], in which we "qualitised" the extracted quantitative data (i.e. we converted it into textual descriptions to facilitate integration with the qualitative data). Due to this, we have not added quantitative findings.

[1]JBI Manual for Evidence Synthesis. 8.2 Concepts and considerations for mixed methods systematic reviews 2024 [Available from: https://jbi-global-wiki.refined.site/space/MANUAL/355829239/8.2+Concepts+and+considerations+for+mixed+methods+systematic+reviews.

Scope and objectives

4. The scope could be more explicitly framed in the introduction. While the manuscript addresses gaps in research, the objectives section would benefit from directly emphasizing these gaps and making the economic implications of the QbTest a more prominent part of the discussion.

Thank you. We consider that the objectives section should state the objective for the review, whereas emphasizing the gaps in the research belongs more in the results and discussion sections. We have therefore covered gaps in the research in those sections.

5. Add a sentence in the objectives section highlighting that the study focuses on children and adolescents, with limited data on adults.

Thank you. This is covered within the results and discussion, rather than the objectives. We believe this is appropriate because it is a finding of our review.

6. Additionally, emphasize that future research should explore cost-effectiveness to guide healthcare decision-making.

Thank you for your comment. As noted above, suggestions for future research are typically presented in the discussion rather than the objectives and we feel this works better for this review. In the discussion, we have emphasized that further research is required to explore the cost-effectiveness of the QbTest: "Further costs data are also needed. A recent review found that the use of the QbTest in addition to clinical assessment is likely to be cost effective, however it was restricted by data limitations.(18) Therefore, future studies should also conduct economic analyses, with long-term cost data, that includes costs for education and healthcare settings.".

Method

7. The methodology is robust, adhering to PRISMA-2020 guidelines and registering the protocol in PROSPERO. The comprehensive search strategy across multiple databases demonstrates a commitment to thoroughness. The mixed-methods synthesis using the convergent-integrated approach is a key strength, allowing for nuanced insights. However, some aspects of the methodology could be clarified. The reliance on single-reviewer data extraction, even with cross-checking, could introduce bias, and this should be acknowledged and addressed.

Thank you. Conducting single data extraction and risk of bias assessment with rigorous checking by a second reviewer is in line with recommendations by the ROBIS tool[1]. We consider this to be robust methodology and we use it for all of our reviews.

[1]Whiting P, Savović J, Higgins JP, Caldwell DM, Reeves BC, Shea B, Davies P, Kleijnen J, Churchill R. ROBIS: a new tool to assess risk of bias in systematic reviews was developed. Journal of clinical epidemiology. 2016 Jan 1;69:225-34.

8. Furthermore, the exclusion of other continuous performance tests (CPTs) like Nesplora or EFSim warrants a more detailed justification. Provide a more detailed explanation for focusing on QbTest over other CPTs, such as its research prominence or NICE recommendation.

Thank you for highlighting this. We have added a sentence to the beginning of the methods section to justify the focus on the QbTest: "We focus on the QbTest because it is the CPT with motion tracker that is currently most commonly used in practice." This is also emphasised in the introduction.

9. **"The searches were drawn from our broader Diagnostic Assessment Report conducted for NICE" - Expand upon this and very briefly situate NICE for readers who are unfamiliar.**

Thank you. We have amended this sentence to cite the broader Diagnostic Assessment Report and to situate NICE for readers: "This review formed part of a Diagnostic Assessment Report that had a broader scope and was conducted to inform guidance by NICE: an organisation that produces evidence-based guidance on new health technologies for the National Health Service in England and Wales.(18)"

10. **"All data items are provided in supplementary material" - Refer to which appendix or file specifically.**

Thank you for highlighting this. We have done this, as follows: "All data items are provided in supplementary material (appendix 5)."

11. **"Qualitative data (we excluded question 10 "how valuable is the research?")" - Very briefly explain the rationale behind this.**

Thank you. The CASP checklist is a general appraisal checklist but we deemed it to be the best available tool to use to assess the risk of bias in the qualitative studies included in this review. We excluded the final item of the tool ("how valuable is the research?") because we felt it is more of a critical appraisal judgement than an assessment of the risk of bias in the study. We have added this justification to the relevant text under quality assessment: "and an amended version of the CASP checklist for studies that contributed qualitative data (we excluded question 10 "how valuable is the research?" as this is a critical appraisal judgement rather than an assessment of risk of bias)."

12. **"There were not sufficient data reported to stratify by subgroups." - Why? What was your criterion? Be more specific with your justification, e.g., provide the n's of subgroups, etc.**

Thank you for your comment. There were no data stratified by the subgroups pre-specified in our protocol, so we were unable to undertake subgroup analyses. We have amended this sentence in the data synthesis section to make this clearer: "We had planned to conduct subgroup analyses, where data were available, to determine whether results differed according to age, sex, ethnicity, people with mental health, behavioural and neurodevelopmental conditions, people with developmental trauma, people in the Youth Justice System or Adult Criminal Justice System, and looked-after children. However, no data were available stratified by these subgroups."

Results

13. **The results section is comprehensive and integrates quantitative and qualitative findings effectively, providing a clear picture of the QbTest's impact on diagnostic processes and user experiences. A notable strength is the exploration of barriers to implementation, such as technological and staffing challenges, which adds practical value to the findings. However, some results lack quantitative specificity, which reduces their impact. For example, while the reduction in diagnostic time is mentioned, exact figures are absent.**

Thank you. Please see our reply to your comment no3, which notes that we used the convergent-integrated approach to mixed-methods synthesis, in which we "qualitised" the extracted quantitative data, and therefore we do not report quantitative data in the thematic findings. However, the quantitative data are presented in full in appendix 5.

14. **Additionally, subgroup analyses by sex, ethnicity, or comorbidities are not provided, limiting the generalizability of the findings.**

Thank you. As noted in our response to your comment no12, the included studies did not stratify data by these key subgroups, so we were unable to conduct subgroup analyses. We agree that this is

a limitation of the available evidence and we highlight this in the discussion: "Additionally, most studies were conducted in children (predominantly white males in the studies that reported sex and/or ethnicity) within the UK. It is therefore not clear whether results would be similar in adults, in other countries, or in subgroups, such as people with comorbidities or females who tend to mask symptoms.(40)"; "Future RCTs should be powered to explore the impact of the QbTest in subgroups of patients including age, sex, ethnicity, and people with comorbidities".

15. **If possible, include numerical data for key outcomes, such as the average reduction in diagnostic time (e.g., "reduced by X days") and improvements in clinician confidence (e.g., "increased by Y%"). Acknowledge the lack of subgroup analyses and its implications for understanding how findings might vary across demographics.**

Thank you for your comment. Please see our reply to your comment no3 concerning our use of the convergent-integrated approach to mixed-methods synthesis, in which we "qualitised" the extracted quantitative data, and therefore do not include numerical data in the thematic findings. However, all quantitative data are presented in appendix 5.

We have added to the results section that the included studies did not stratify by key subgroups: "Findings should also be interpreted within the context of the study characteristics outlined in **Error! Reference source not found.**. Nine of the 10 included studies children and young people and studies did not stratify by key subgroups (e.g. age, sex, ethnicity, comorbidities)." We discuss the potential implications of this for understanding how findings might vary across demographics in the discussion.

16. **"Most studies reported both quantitative and qualitative data" and other similar statements - provide the number or percentage. That said, for # of studies endorsing a finding (e.g., Three studies were RCTs…), would love to see the percentage too.**

Thank you. We have amended the sentence beginning "most studies reported both quantitative and qualitative data" to be more specific, as suggested: "As outlined in **Error! Reference source not found.**, six studies reported both quantitative and qualitative data;(30, 32-37) the other four studies reported quantitative data. Seven studies enrolled participants with suspected ADHD,(30, 32-36, 38, 39) two enrolled participants with an ADHD diagnosis,(37, 40) and one study (survey of patients from a diagnostic test accuracy study who had used the QbCheck) enrolled 59 participants with ADHD and 69 "healthy" controls.(31)." We have not added percentages here, because there are only ten studies in total, so we feel it may not provide much added benefit to the reader.

17. **"We do not include quantitative patient outcome data from the FACT and QUOTA feasibility RCT studies, because, as noted, they were not designed or powered to evaluate impact on outcomes." - Provide a citation behind the methodological rationale for this.**

Thank you. We have added a citation to a paper that describes feasibility studies[1], i.e. studies conducted before a main study to explore whether the main study can be done (and so not to evaluate impact on outcomes).

[1]Eldridge SM, Lancaster GA, Campbell MJ, Thabane L, Hopewell S, Coleman CL, Bond CM. Defining feasibility and pilot studies in preparation for randomised controlled trials: development of a conceptual framework. PloS one. 2016 Mar 15;11(3):e0150205.

18. **Headings/subheadings within Results are confusing, particularly "Findings" - maybe change that one to "Thematic Findings". I would also move Quality Assessment to the end of results; it is more intuitive to read Characteristics of Articles, than Thematic Findings.**

Thank you. We have changed the heading "Findings" to say "Thematic findings". We have kept quality assessment before the thematic findings because we think that it is important for the reader to see these first, so they can interpret the thematic findings in light of the risk of bias assessments.

This is particularly important as most studies were judged at high risk of bias. We state this at the beginning of the thematic findings: "Findings should be interpreted with caution due to the aforementioned concerns with risk of bias."

19. **Regarding Quality Assessment - provide a brief summary sentence before saying "Quality assessments are provided in Appendix 5", as this sentence appears dropped. Clarity in this section is important because your findings depend on many high-risk-of-bias studies.**

Thank you. We have removed this sentence and instead refer to appendix 5 within each of the quality assessment sub-sections.

20. **Regarding the thematic findings, the language is a little precarious. I think what you are calling "themes" (i.e., views on the test; barriers) are actually the conceptual categories rather than the themes. One can certainly expect that there will be views on the test and barriers - these don't really thematically explain what the findings are telling us. However, what you are referring to as conceptual categories are in fact the themes, i.e., contribution to ADHD diagnosis, which then broadly fall into the categories of "views" and "barriers". Please fix this in the Abstract too.**

Thank you for your comment. We agree that "contribution to ADHD diagnosis" and "barriers to implementation of the test" are our conceptual categories, constructed around the themes that we identified from the data. This is correctly stated in the "data synthesis" section, but we recognise that in some parts of the manuscript this was not as clear. We have therefore amended the abstract (second paragraph of the results section) to say: "We organized themes emerging from the qualitative synthesis into two broad conceptual categories: views around the helpfulness of the QbTest (contribution to ADHD diagnosis, treatment decision making, communication with caregivers) and barriers to QbTest implementation (practical barriers and acceptability of the test to patients and caregivers)."

We have also amended the first sentence under the heading "thematic findings" to say: "We organized themes emerging from the qualitative synthesis into two broad conceptual categories: 1) views around the helpfulness of the QbTest (contribution to ADHD diagnosis, treatment decision making, and communication with caregivers), and 2) barriers to the implementation of the QbTest (practical barriers and the acceptability of the QbTest to patients and caregivers)."

21. **Once again with the headings - I am finding it very difficult to at a glance figure out what the thematic findings are. For example, under "views around helpfulness of the test", I feel like the "Contribution to ADHD diagnosis" heading is quite broad and vague. I would suggest using subheadings (subthemes) for themes, e.g., for "Contribution to ADHD diagnosis", you could have the subthemes "Time Until Diagnostic Decision", "Proportion of People with Diagnostic Decision", "Clinician Confidence", etc. In this fashion, I would revise all the existing headings. You did this for the "Barriers" category, so make it consistent.**

Thank you. In response to this comment and your comment no23, we have drawn out the sub-themes in a new table (Table 2) to provide a clear overview of the themes. We have also added topic sentences, as you suggest in comment no22, to each theme to outline what they cover. We are keen not to add sub-headings to the narrative summary as we feel it will detract from the flow of the narrative.

22. **Define terms like "Contribution to ADHD diagnosis" and "treatment decision making" when you first mention them - what do these concepts entail? A way to do this might be to provide a topic sentence mapping out the contents of the paragraph so the reader knows what to expect at the beginning of each theme, e.g., "we found XYZ regarding contributions to ADHD**

diagnosis, i.e., time until diagnostic decision, proportion of people with diagnostic decision…." Etc.

Thank you. As suggested, we have added a topic sentence to the beginning of each theme, to help the reader to know what to expect for each theme.

23. **To make everything abundantly clear, I would actually recommend providing a table with the columns "category", "theme", and "subtheme", along with the frequency/percentage of endorsement by articles. An example of such a table is Table 2 here: Sharma RH, Rodberg D, Struik LL Experiences of nicotine users motivated to quit during the COVID-19 pandemic: a secondary qualitative analysis BMJ Open 2023;13:e070906. doi: 10.1136/bmjopen-2022-070906. This is just my own work off the top of my head, but don't feel limited by this - there are other examples in the literature too. You might have to play around with the format as this is a review and not an empirical study, but I believe such a table would make the findings clearer and more translatable.**

Thank you for this suggestion. We have added the below table (Table 2) to provide an overview of the thematic structure and the number of studies providing data for each theme.

**Conceptual category**	**Theme**	**Subtheme**	**No. studies providing qualitative data**	**No. studies providing quantitative data**
Views around the helpfulness of the QbTest	Contribution to ADHD diagnosis	Time to diagnostic decision	4	5
		Proportion of patients with a diagnostic decision	0	2
		Clinician confidence in the diagnostic decision	3	1
		Diagnosis in complex cases	3	1
		Cost	0	2
	Treatment decision making	No sub-themes identified	5	3
	Communication with caregivers	No sub-themes identified	3	4
Barriers to the implementation of the QbTest	Practical barriers	Scheduling	1	0
		Space	4	0
		Staffing	4	0
		Technology	4	0
	Acceptability of the QbTest to patients and caregivers	No sub-themes identified	3	4

24. **Table 2 with quotes is kind of dropped haphazardly. Discuss the quotes/experiences a bit more in the writing, or build the quotes into the narrative.**

Thank you. We have removed the quotes table and we now include illustrative quotes throughout the narrative. We highlight this at the end of the third paragraph under the heading "thematic findings" to say: "Illustrative quotes are included throughout and all extracted data are provided in Appendix 5".

25. **That said, the whole theme of Barriers it not discussed nearly as much as the first theme. I would appreciate a little more discussion around it, if the word limit allows. See the next comment for additional implications on this.**

Thank you. We have added more detail, as follows: “This theme focuses on practical barriers to QbTest implementation, including scheduling, space, staffing and technology. Qualitative evidence (five studies) reported barriers to QbTest implementation.(28, 34-36, 38) One study highlighted potential scheduling issues caused by the need for more appointments for medication management.(35) Four studies reported concerns with room requirements for conducting the QbTest. For example, it was reported that a room is required to be able to administer the QbTest, and sometimes this is hard to arrange, which means the equipment may need to be moved between rooms.(28, 34, 38) Focus groups with clinicians in CAMHS also highlighted concerns about managing environmental factors influencing the QbTest, as the test needs to be conducted in a quiet environment.(36)

“The main [challenges] were just the practical side, like the room space and things. It’s really competitive to get rooms here so making sure it was booked well in advance.”(34) – Healthcare professional

Four studies highlighted staffing as a potential barrier, as they noted that a trained staff member must be available to conduct the QbTest.(28, 34, 36, 38) Additionally, four studies reported technology as a barrier, including issues with accessing laptops, sharing passwords, internet connection, and printer access.(28, 34, 36, 38)

“There was a lot of IT [Information Technology] governance issues to get it set up”(38)- Healthcare professional””

26. **Overall, a broader discussion of patient experiences is needed, as ADHD is historically diagnosed, assessed, and treated to primarily alleviate how the ADHD individual impacts third parties (parents, teachers) rather than address how the ADHD individual feels themselves. Therefore, if you are making a statement like “well received by users” (Abstract), this needs to be backed up with a broader discussion of how the population in question experiences it too, which may be doubly challenging as the population you are focusing on in this paper is children/youth. The only quote you provide on “Acceptability to patients and caregivers” isn’t a patient or caregiver quote at all - provide patient quotes if possible. If you don’t have this data, you can’t make that statement; consider revising to “well-received by clinicians using it”. Consider that throughout the paper, especially since the only thing differentiating your paper from other quantitative reviews on this topic is the mixed-methods nature of it, i.e., inclusion of qualitative experiences.**

Thank you for your comment. Throughout our thematic findings, we provide evidence on the patient and carer experience of using the QbTest, where this has been reported by included studies. More quotes appeared to be published in the reports by staff than patients and we highlight in our discussion that further studies are needed that measure patient outcomes and user experience of the test. We have amended this to make it clearer: “Studies should measure patient outcomes and patient, carer, and clinician experience of using the test. They should prioritise outcomes that were shown to be important to patients in the qualitative evaluations (e.g. communication, understanding and acceptance of diagnostic decision, acceptability of the test to patients).”

Where we stated in the abstract that “the QbTest appeared to be generally well received by users”, we were referring to the overall picture given by the thematic findings, rather than only focusing on the “acceptability” theme. We do feel this to be true, as the available evidence from patients and carers did highlight positive views on the potential impact of the test (e.g. some families felt it could improve time to diagnosis and that seeing the QbTest results made them feel more confident that ADHD medication would help). We have therefore decided to keep this statement in the abstract, but

we have amended it to attribute the reported barriers to clinicians and patients, respectively, so that it is clear that some patients did highlight that they found the test long and repetitive: "The QbTest appeared to be generally well received by clinicians, patients and carers. However, barriers to test implementation were reported. Clinicians cited staffing, room requirements and issues with technology, and patients highlighted the test length and repetitive nature."

Regarding the quote for the theme "acceptability to patients and caregivers", unfortunately, the studies that interviewed patients and/or caregivers on the acceptability of the QbTest did not provide any quotes from patients, so this is why we only have one quote from staff for this theme. We have amended this to make it clearer by stating "(no quotes published)" where we discuss qualitative findings from patients and "patients were not interviewed about their perception of the test in this study" where we discuss the results of the Focus ADHD study.

Discussion

27. **Use of subheadings e.g., summary of findings, strengths and limitations, future directions, conclusions, etc., could strengthen the discussion.**

Thank you. We have added subheadings to the discussion, as suggested.

28. **"The implementation of the QbTest may further impact upon the availability of ADHD medication by exposing unmet need for treatment." - I agree with what you said prior to this, that medication is not the only line of treatment. What other treatment modalities can the QbTest support? Be specific so that readers understand that ADHD is treated holistically - most papers unilaterally focus on medications which has created a skewed understanding of ADHD among health practitioners, ADHD individuals, and the general public.**

Thank you. We have amended this text to highlight other treatment modalities: "Whilst medication is not the only treatment option for those with ADHD, it is worth noting that shortages of ADHD medication have been reported, due to increased global demand and issues with manufacturing.(42) The implementation of the QbTest may further impact upon the availability of ADHD medication and other ADHD management strategies (e.g. behavioural interventions, educational and psychosocial support) by exposing unmet need for treatment."

29. **I would caution against endorsing the QbTest too much as you do have quite mixed findings with very high-risk-of-bias studies. Language that is tentative and specific, such as "may improve the ADHD diagnostic process, when used alongside clinical assessment in children and young people" is appropriate. Ensure you don't extend beyond this.**

Thank you. We believe that we clearly portray the limitations of the evidence and use appropriate phrasing so as not to over-endorse the QbTest. We highlight concerns with risk of bias in the discussion, stating in the first paragraph that "Many of the included studies were judged at high risk of bias, due to issues with missing outcome data, confounding, or insufficient reporting, so results should be interpreted with caution." Additionally, throughout the discussion, we use phrasing "may improve" and in agreement with your suggestion, our conclusion states "the available evidence suggests that the QbTest may improve the ADHD diagnostic process, when used alongside clinical assessment in children and young people".

We noticed that in the abstract we had stated the QbTest "can be a useful addition…". We have amended this to align with our conclusion in the discussion, by changing it to say: "The available

evidence suggests the QbTest may be a useful addition to ADHD assessment in children and young people."

30. Is there any literature synthesizing evidence (e.g., review) that ultimately did not endorse the QbTest? It would be interesting to compare findings and would allow for a more nuanced discussion.

We have not come across any systematic reviews of this nature in our searches.

31. I would include a "future directions for practice" paragraph where you discuss possible strategies to remediate some of the practical limitations of the QbTest that you outlined, i.e., barriers.

Thank you. We discuss this in the "implications" paragraph, where we say: "Clinicians require infrastructure to be in place before the QbTest can be implemented, for example a quiet room with a computer, time to be able to administer the test, and training to support the effective completion of the test and communication of test results. The positioning of the QbTest on the diagnostic pathway also needs to be carefully considered and agreed, to reduce variation in the potential impact of the test across healthcare services." We are conscious not to lengthen the manuscript further as we have already added quite a lot of words with the quotes etc and adding more paragraphs to the discussion would exceed the limit given by BMJ Open.

32. Overall, this paper is well-written and has potential once the above are addressed!
Thank you again for your helpful feedback.

VERSION 2 - REVIEW

**Reviewer**	**1**
**Name**	**Hall, Charlotte**
**Affiliation**	**Institute of Mental Health, NIHR CLAHRC-East Midlands, Psychiatry**
**Date**	**17-Mar-2025**
**COI**	

I have no further comments. Thank you for addressing all comments clearly.

**Reviewer**	**2**
**Name**	**Sharma, Ramona**
**Affiliation**	**The University of British Columbia Okanagan, School of Social Work**
**Date**	**08-Mar-2025**
**COI**	

Thank you for the comprehensive responses, justifications, and/or edits you have provided with respect to each comment.

In my opinion, the authors have revised the paper sufficiently and this paper can now proceed to publication.
